# Comorbidities of Psoriasis - Exploring the Links by Network Approach

**DOI:** 10.1371/journal.pone.0149175

**Published:** 2016-03-11

**Authors:** Sudharsana Sundarrajan, Mohanapriya Arumugam

**Affiliations:** Division of Bioinformatics, School of Biosciences and Technology, Vellore Institute of Technology University, Vellore, Tamil Nadu, India; Harbin Medical University, CHINA

## Abstract

Increasing epidemiological studies in patients with psoriasis report the frequent occurrence of one or more associated disorders. Psoriasis is associated with multiple comorbidities including autoimmune disease, neurological disorders, cardiometabolic diseases and inflammatory-bowel disease. An integrated system biology approach is utilized to decipher the molecular alliance of psoriasis with its comorbidities. An unbiased integrative network medicine methodology is adopted for the investigation of diseasome, biological process and pathways of five most common psoriasis associated comorbidities. A significant overlap was observed between genes acting in similar direction in psoriasis and its comorbidities proving the mandatory occurrence of either one of its comorbidities. The biological processes involved in inflammatory response and cell signaling formed a common basis between psoriasis and its associated comorbidities. The pathway analysis revealed the presence of few common pathways such as angiogenesis and few uncommon pathways which includes CCKR signaling map and gonadotrophin-realising hormone receptor pathway overlapping in all the comorbidities. The work shed light on few common genes and pathways that were previously overlooked. These fruitful targets may serve as a starting point for diagnosis and/or treatment of psoriasis comorbidities. The current research provides an evidence for the existence of shared component hypothesis between psoriasis and its comorbidities.

## Introduction

Psoriasis is a chronic immune mediated skin disease. The disease presents itself with well circumscribed, red or silvery scaly plaques [1].The patho-physiology of psoriasis is characterized by increased production of T lymphocytes and up regulation of type 1 T helper cells [2].Though numerous scientific communities contribute to psoriasis research around the world, the etiology of the disease still remains unidentified. The evolving evidences suggest psoriasis as a complex disorder coordinated by the interplay between multiple genes [3].

The occurrence of one or more disorders in association with a particular disease has recently gained interest in various medicinal divisions including dermatology [4–5]. Multiple observational studies demonstrate the associations between psoriasis and several comorbidities [6–7]. Psoriasis shares common immunological features with many complex disorders such as cardiovascular disease, diabetes, obesity, depression and inflammatory arthritis [8]. The concurrence of the disease with other disorders such as Chron’s and Alzheimer’s disease are also observed [9]. However, the patho-mechanism connecting these systemic comorbidities with psoriasis remains to be determined. A systematic exploration of the shared component hypothesis existing between the co-morbidities can shed light about the molecular connections aiding the prevention, early diagnosis and treatment of psoriasis [10]. Network medicine a branch of systems biology provides a systematic platform to investigate the molecular intricacy of a disease aiding the identification of new molecular associations among apparently diverse clinical manifestations [11–12].

The current work aims to decipher the shared component hypothesis between psoriasis and its five associated common comorbidities which includes myocardial infarction (MI), type II diabetes (T2DM), obesity, rheumatoid arthritis (RA) and Alzheimer’s disease (AD). The psoriasis associated comorbidities would be associated at the molecular level by common genes, proteins, biological processes and pathways. The network medicine approach has been utilized to construct individual diseasomes. The interactomes are explored to identify the biological processes and pathways linking psoriasis with its comorbidities.

## Materials and Methods

### Gene expression data

Gene expression raw data (CEL files) were downloaded from NCBI GEO database [13]. A single dataset for each disease category was used for the analysis. For each disease, the dataset was selected only if it enclosed a minimum of five samples in both disease and control category. The number of samples and their respective platform details are reported in **Table 1**.

**Table 1:** Gene expression datasets used for the analysis.

Accession ID	Disease	Number of samples	Contributor
GSE4757	Alzheimer’s disease	20	Barth AS *et al*.,
GSE25724	Type II diabetes	13	Dominguez V *et al*.,
GSE3585	Myocardial infarction	12	Barth AS *et al*.,
GSE9624	Obesity	11	Aguilera CM *et al*.,
GSE48780	Rheumatoid arthritis	83	Dennis G Jr *et al*.,
GSE13355	Psoriasis	180	Gudjonsson JE *et al*.,

### Microarray gene expression processing

The microarray expression sets were processed individually in a similar way to remove bias. The raw files from different studies were processed using the bioconductor packages in R [14–15]. The pre-processing of the microarray data involves background adjustments, quantile normalization and summarization using GeneChip Robust Multiarray Averaging (GCRMA). Probes with multiple entries were restricted to single entry by taking mean of their expressions. The probes without any gene affiliations were removed from further analysis. Student’s unpaired T-test was performed to identify genes that were differentially expressed between the diseased and normal samples. A threshold of at least 1 fold change and a p-value less than 0.5 were chosen as criteria for selecting the genes. The data sample for RA had entries only for the diseased category, hence normal samples isolated from synovial tissues of normal donors (GSE1919) was used for comparison.

### Diseasome construction

The association between psoriasis and its comorbidities was termed as “diseasome”. The two diseases were linked if they share the variations in similar set of genes. Proteins encoded by each differentially expressed gene were identified for the construction of the diseasome. The human proteome interactome was obtained based on the interactions reported by the HPRD server [16].The strength of the association between two diseases in the diseasome was assessed using molecular comorbidity index (MCI). MCI is defined as;

$${\text{MCI}}_{\text{Dis}1,\text{Dis}2}=\frac{\left|({\text{Proteins}}_{\text{Dis}1}\cap {\text{Proteins}}_{\text{Dis}2})\cup ({\text{Proteins}}_{\text{Dis}1\to \text{Dis}2})\cup ({\text{Proteins}}_{\text{Dis}2\to \text{Dis}1})\right|}{\left|({\text{Proteins}}_{\text{Dis}1}\cup {\text{Protein}}_{\text{Dis}2})\right|}$$

Proteins_Dis1_ Proteins and Protein_Dis2_ Proteinswere the proteins associated with disease1 and disease2 respectively. Proteins_Dis1→Dis2_ Proteinswere the proteins associated with disease1 that show interactions with the proteins associated with disease2 (vice versa for Proteins_Dis2→Dis1_ Proteins). ∩ was the intersection operator denoting the number of common proteins between the diseases and ∪ operatordenote the total number of proteins participating in both the disease categories. The sets represented within the vertical bars indicate their cardinality.

#### Functional analysis of the comorbidity proteins

To explore the significance of biological functions of the differentially expressed proteins in each disease [17] a functional enrichment analysis was carried out using DAVID server [18] and PANTHER classification system [19]. The biological processes and pathways shared by the diseases with psoriasis was assessed using Jaccard coefficient, which is defined as:

$${\text{Jaccard coefficient}1}_{\text{Dis}1,\text{Dis}2}=\frac{\left|{\text{BP}}_{\text{Dis}1}\cap {\text{BP}}_{\text{Dis}2}\right|}{\left|{\text{BP}}_{\text{Dis}1}\cup {\text{BP}}_{\text{Dis}2}\right|}$$

$${\text{Jaccard coefficient}2}_{\text{Dis}1,\text{Dis}2}=\frac{\left|{\text{Pathways}}_{\text{Dis}1}\cap {\text{Pathways}}_{\text{Dis}2}\right|}{\left|{\text{Pathways}}_{\text{Dis}1}\cup {\text{Pathways}}_{\text{Dis}2}\right|}$$

The Jaccard coefficient measures the degree of similarity between the psoriasis comorbidities. Dis1 and Dis2 correspond to psoriasis and its comorbidities. Biological process (BP) of Dis1 and Dis2 represents the biological processes contributed by psoriasis and its individual comorbidity respectively, whereas pathways of Dis1 and Dis2 are the biological pathways in which the proteins associated with the comorbidities participate. The calculated measures were visualized as heat-maps constructed using gitools [20].

## Results

The differential expression analysis of psoriasis microarray data resulted in 507 differentially expressed genes (DEG) (113-upregulated; 394-downregulated), on submitting the differentially expressed genes of psoriasis transcriptome to DAVID server, around fourteen major disease categories were enriched (**Table 2**). Each disease category had a minimum of one disease and a maximum of four disease classes. Five diseases based on their common co-occurrence with psoriasis were selected to study their relationship with psoriasis. The comorbidities associated with psoriasis chosen for the study were T2DM, obesity, MI, AD and RA. The association between psoriasis and the five major comorbidities were verified against the published literatures [21–25].

**Table 2:** Disease categories associated with psoriasis based on the genes involved.

Disease class	Disease	# genes	Genes involved
Musculoskeletal Diseases	RA	16	PLAT, MMP9, TLR2, GGH, PTPN22, CXCR2, MMP1, MMP12, TYMS, IL12RB1, CCR5, CD274, IL1B, SERPINA1, FCGR3B, SELE
Neoplasms	Esophageal cancer	4	TYMS, IL8, CXCR2, MMP1
Neoplasms	Stomach Neoplasms	5	TYMS, IL8, CXCR2, MMP1
Neoplasms	Leiomyoma	3	IL12RB1, IL8, IL1B
Neoplasms	Oral cancer	5	IL8, MMP9, TGFA, CYP2E1, MMP1
Virus Diseases	Hepatitis C	10	IFI27, IL12RB1, CCR5, LDLR, IL19, IL1B, OAS1, CXCR2, MX1, IL20
Virus Diseases	Hepatitis B	6	CCR5, OAS3, OAS1, OAS2, MX1, STAT1
Otorhinolaryngologic Diseases	Hearing loss/deafness	5	PLAT, SLC26A4, GJB6, ESPN, GJB2
Bacterial Infections and Mycoses	Tuberculosis	9	IL12RB1, IL8, TLR2, PTPN22, IL1B, CXCR2, SERPINA1, CYP2E1, STAT1
Nervous System Diseases	Subarachnoid hemorrhage	5	MMP9, SERPINA3, IL1B, MMP12, MMP1
Nervous System Diseases	Multiple sclerosis	15	IL8, MMP9, CCR1, APOC1, PTPN22, CXCR2, OAS1, IL7R, CXCL10, CCR5, IL1B, CD24, MX1, FCGR3B, SELE
Nervous System Diseases	AD	3	C3,CD14, DNM3
Digestive System Diseases	Pancreatitis, chronic	5	IL8, PRSS2, PRSS3, SERPINA3, IL1B
Stomatognathic Diseases	Periodontitis	9	PLAT, CCR5, S100A8, MMP9, TLR2, IL1B, SERPINA1, FCGR3B, MMP1
Respiratory Tract Diseases	COPD	7	IL8, MMP9, SERPINA3, IL1B, SERPINA1, MMP12, MMP1
Respiratory Tract Diseases	Asthma	15	CYP2J2, MMP9, TLR2, CXCR2, EHF, IL12RB2, CCR5, CXCR4, FCGR1B, SERPINA3, FUT3, IL1B, SERPINA1, FUT2, SELE
Cardiovascular Diseases	Atherosclerosis	13	PLAT, F12, CYP2J2, LDLR, CCR5, SELL, MMP9, APOC1, TLR2, IL1B, FCGR3B, SELE, MMP1
Cardiovascular Diseases	MI	3	JAK2, CD14, CCL1
Cardiovascular Diseases	Coronary artery disease	7	PLAT, CCR5, LDLR, MMP9, IL1B, SELE, MMP12
Digestive System Diseases	Cirrhosis; pancreatitis	3	IL8, IL1B, CYP2E1
Skin and Connective Tissue Diseases	Systemic lupus erythematosus	9	CCR5, IL8, CFB, TLR2, PTPN22, IL1B, CXCR2, FCGR3B, SELE
Hemic and Lymphatic Diseases	Sarcoidosis; tuberculosis	3	IL12RB2, IL12RB1, MMP1
Nutritional and Metabolic Diseases	T2DM	2	IL12RB2, IL12RB1
Nutritional and Metabolic Diseases	Obesity	5	CXCL1, DPN, NAIP, PAPPA, IL24

^#^Number of genes enriched in each disease category; Diseases are classified based on the MeSH disease hierarchy

### Linking psoriasis with its comorbidities

**Fig 1:**
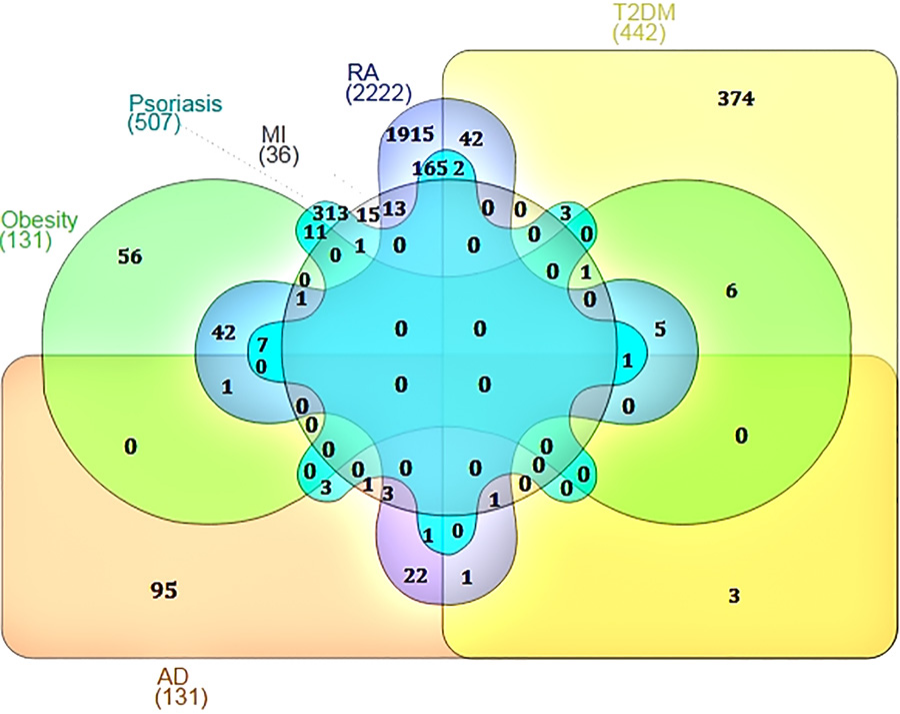
Venn diagram showing the number of overlapping genes between psoriasis and its comorbidities.

Independent meta-analysis was carried out for psoriasis and its co-morbidity using their respective microarray expression data. The aim of the meta-analysis was to identify the genes which were significantly upregulated and down regulated in diseased condition when compared with the normal samples [26–27]. The number of genes differentially expressed and associated with five psoriasis comorbidities ranged from 36 (MI) to 2222 (RA) (**S1 Table**). We constructed a psoriasis specific interactome and utilized the knowledge obtained from the interactome to mine out the commonality between psoriasis and its comorbidities (**Fig 1**). The psoriasis diseasome constructed using the knowledge obtained above revealed that the comorbidities were connected with psoriasis through shared genes or proteins which interact to form the cellular interactome.

**Fig 2:**
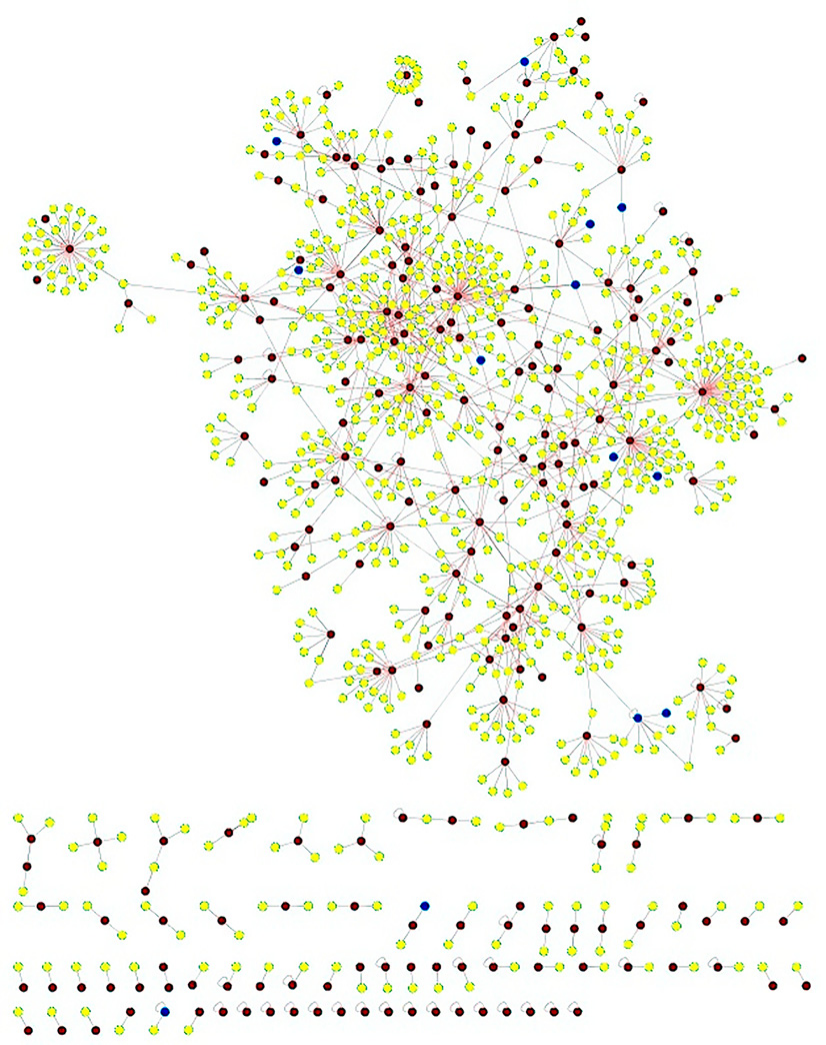
Psoriasis diseaseome along with proteins common in AD. Yellow nodes—proteins involved in psoriasis, red nodes—common proteins shared by AD and psoriasis and blue nodes—indirect interactions.

**Fig 3:**
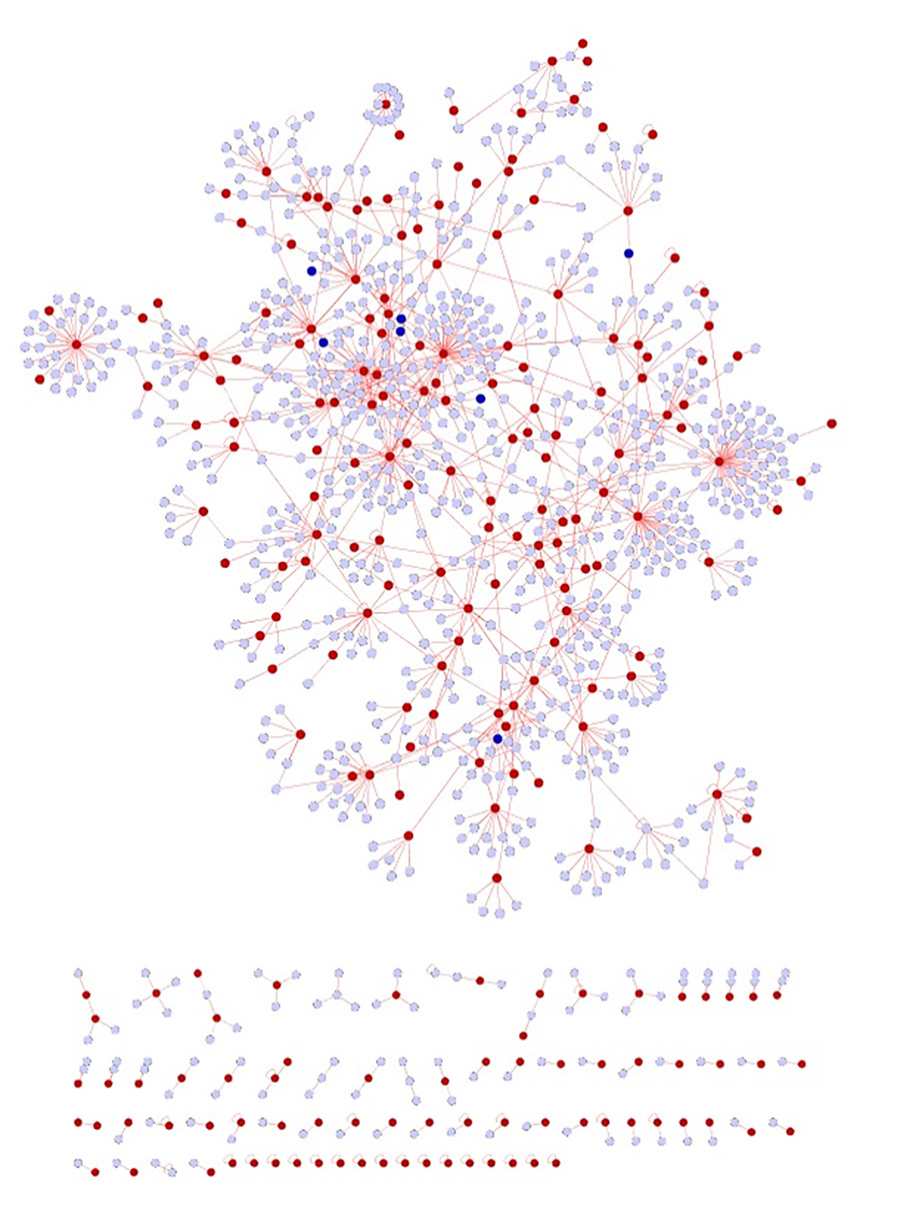
Psoriasis diseasome along with MI proteins. Violet nodes—proteins in psoriasis, red nodes—common proteins shared by MI and psoriasis and blue nodes—indirect interactions.

**Fig 4:**
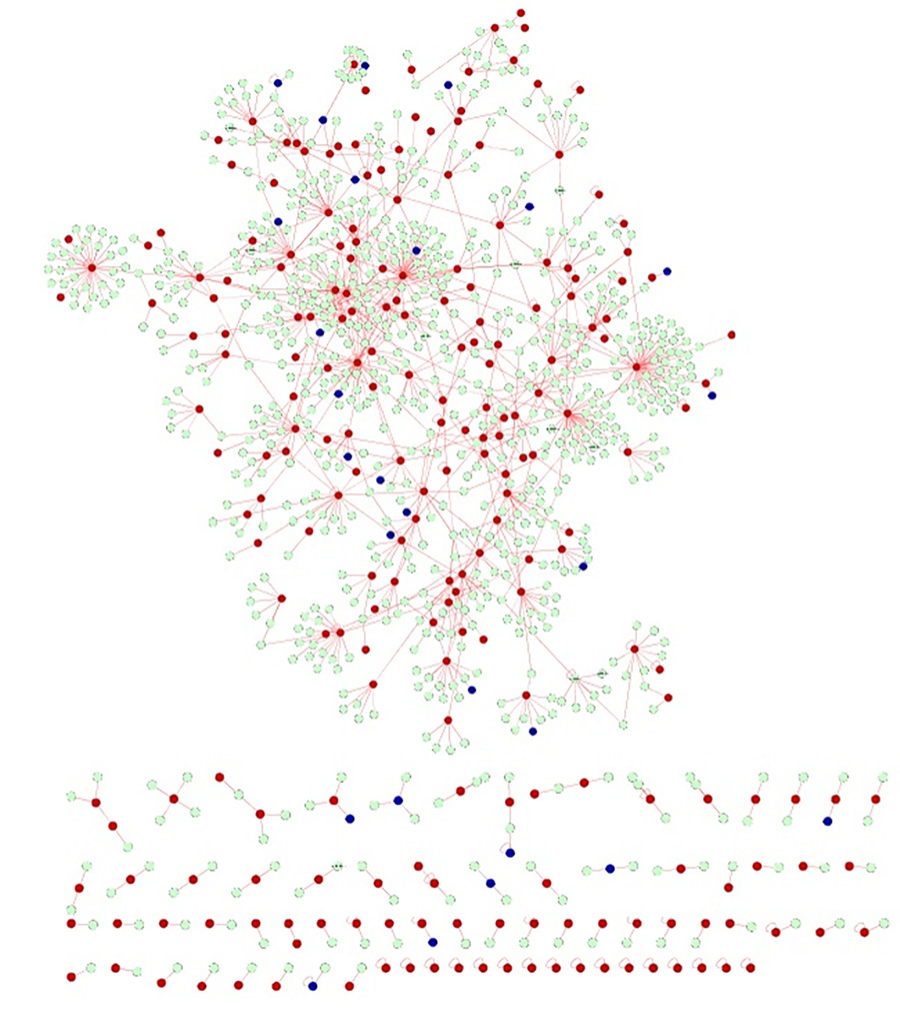
Psoriasis diseaseome highlighting the proteins involved in T2DM. Green nodes—proteins involvement in psoriasis, red nodes—common proteins shared by T2DM and psoriasis and blue nodes—indirect interactions.

**Fig 5:**
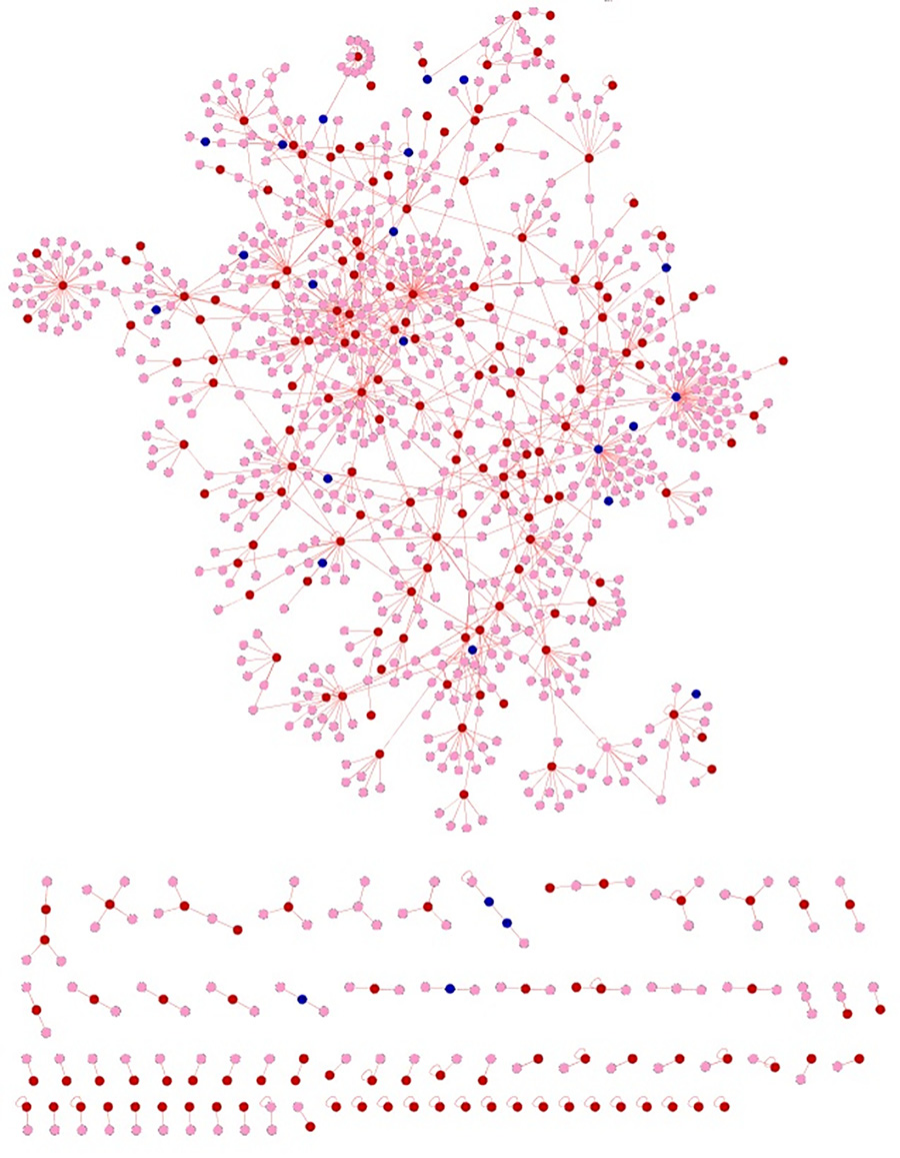
Psoriasis diseaseome enclosing the protein contributors from obesity. Pink nodes—proteins involved in psoriasis, red nodes—common proteins shared by obesity and psoriasis and blue nodes indicate indirect interactions.

**Fig 6:**
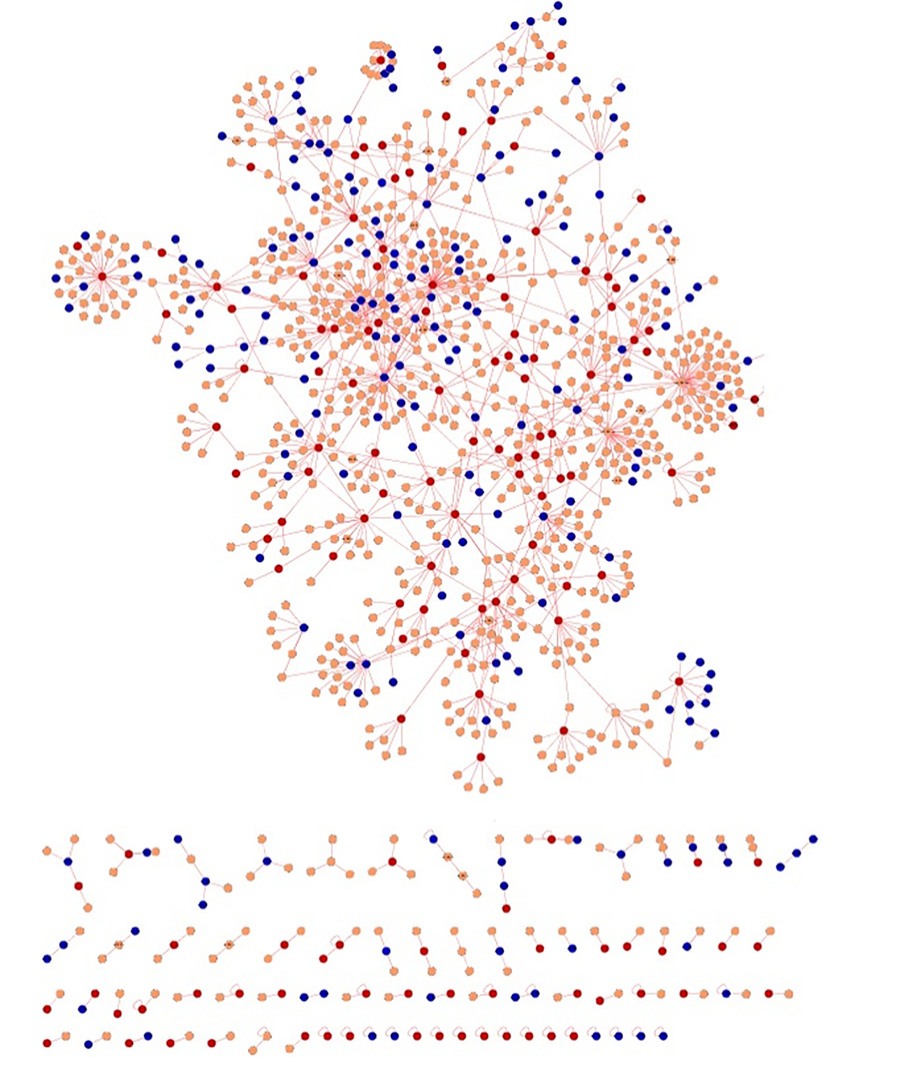
Psoriasis diseaseome including proteins from RA. Brown nodes—proteins involvement in psoriasis, red nodes—common proteins shared by RA and psoriasis and blue nodes—indirect interactions.

**Fig 7:**
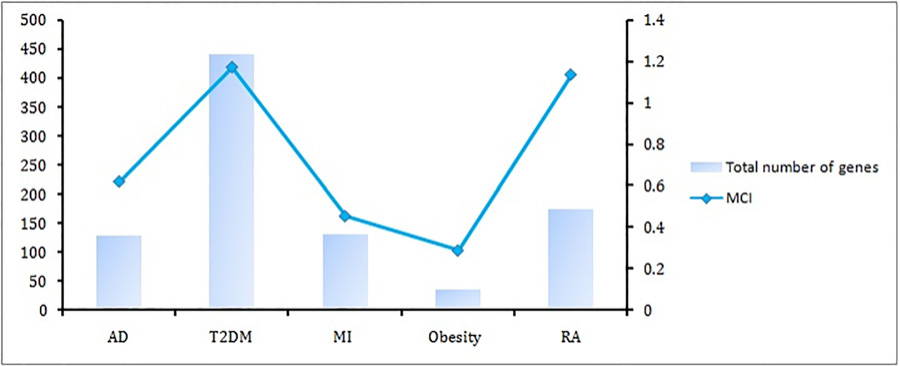
Molecular comorbidity index (MCI). Number of proteins shared between psoriasis and its comorbidities. MCI shows the strength of association between psoriasis and its comorbidities. The association becomes stronger with the increasing number of shared genes and high MCI.

The commonalities between the diseases were assessed based on their interaction patterns. The number of shared genes or proteins in the psoriasis diseasome ranged from 31 (MI) to 312 (T2DM) (**Figs 2–5**). AD and psoriasis were directly linked through 13 proteins, while the association also occur indirectly through the interaction of 84 proteins. In case of T2DM 26 proteins directly connected both the diseases and 210 proteins contribute to their interaction via partnering with other proteins. MI and psoriasis were connected directly through 7 proteins and indirectly through 49 proteins. Similarly obesity and psoriasis were connected directly through 24 proteins and indirectly through 11 proteins. RA showed highest percentage of connections. They were connected directly through 201 proteins and indirectly through 386 proteins (**Fig 6**). The Molecular Comorbidity Index (MCI) which shows the strength of association between psoriasis and its comorbidities was also studied for the better understanding. T2DM leaded the MCI list followed by RA, AD, MI and obesity (**Fig 7**). To assess the role of direct and indirect contributors to the diseases we further studied the differential expression patterns of the linker proteins.

### Direction of expression of the common genes/proteins and linker genes/proteins

**Fig 8:**
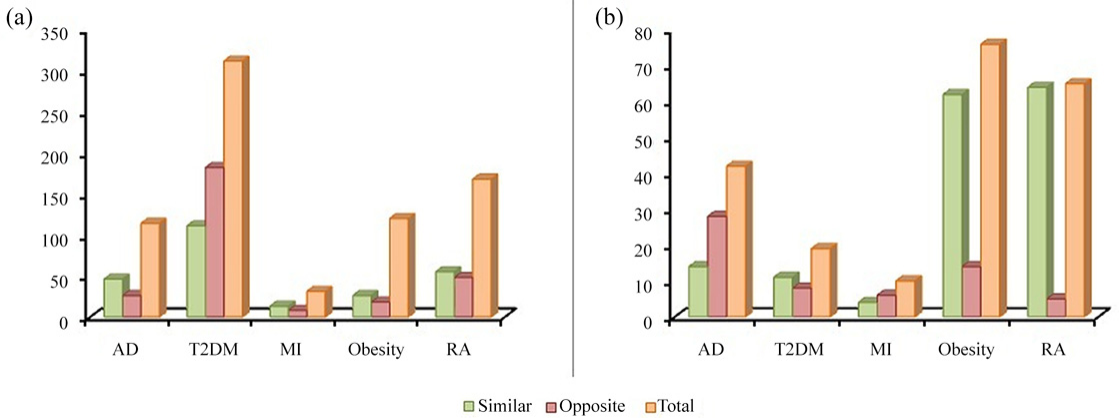
Number of protein regulated in similar and opposite direction. (a) Proteins upregulated (b) proteins downregulated.

The deregulation of common set of genes either in similar or opposite direction can be an underlying cause for the comorbidities. To generate the molecular interpretation of the comorbidities, we compared the direction of dysregulation of the genes shared by psoriasis and its comorbidities with the help of meta-analysis (**Fig 8**). The DEGs of the comorbidities were compared with DEGs of psoriasis individually. We noted significant overlaps between the DEGs upregulated in psoriasis and those downregulated in its comorbidities. Similarly DEGs downregulated in psoriasis were observed to be overlapping with the DEGs upregulated with psoriasis associated comorbidities. Considerable overlap was also observed between DEGs deregulated in same direction between psoriasis and its comorbidities. The DEGs deregulated in the same direction can be treated as putative signatures of the comorbidities while the DEGs deregulated in the opposite direction can contribute to inverse comorbidities. We noted a maximal hit of DEGs deregulated in both the direction was from T2DM followed by RA, obesity, AD and MI. Highest number of genes upregulated in the similar direction was reported in T2DM followed by RA, AD, obesity and MI. Similarly the highest number of down-regulated genes in the same direction was seen in RA followed by obesity, AD, T2DM and MI. The highest number of DEG upregulated in opposite direction followed the same pattern of DEGs upregulated in similar direction while the highest number of DEGs down-regulated in opposite direction was observed in AD followed by obesity, T2DM, MI and RA. When the overall DEG count was compared the genes expressed in similar directions were high contributing to the positive comorbidity.

### Exploring the biological processes shared by the proteins in psoriasis diseasome

**Fig 9:**
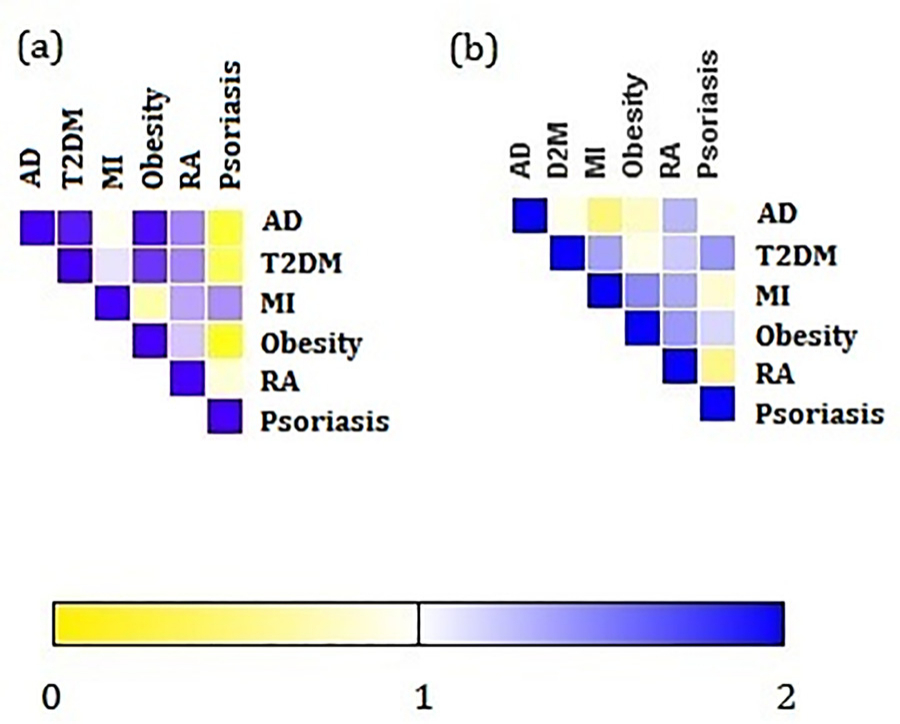
Similarity between psoriasis and its comorbidities. Each cell is colored according to the Jaccard correlation coefficient which represents the similarity between the comorbidities considering, (a) biological process and (b) biological pathways. The MI shares the highest JC in biological process with psoriasis and T2DM shares the highest JC in pathway annotation

To identify the biological functions shared by the psoriasis and its comorbidities, a functional enrichment analysis was performed based on the BP. RA shared the highest number of overlapping BP with psoriasis. The list followed the order of T2DM, obesity, AD and MI starting from highest to lowest overlapping processes (**S2 Table**). Defence response, immune response and inflammatory response were enriched in all the comorbidities. Response to wounding showed a similar pattern of expression in almost all the comorbidities except T2DM. Proteins contributing to cell-cell signaling were upregulated in AD, T2DM and RA. Around five biological processes were shared by obesity and RA with connection to psoriasis. To investigate the degree of similarity between psoriasis and its comorbidities, Jaccard coefficient was computed for all the comorbidities (**Fig 9A**). The highest JC value attributed to obesity followed by T2DM, RA, MI and AD. These observations highlights the critically impaired BP between psoriasis and it comorbidities were from immune mediated processes.

### Overlap of identified biological pathways in the psoriasis diseasome

We further investigated the pathways that were common between the psoriasis and its associated comorbidities (**S3 Table**). Highest number of pathway overlap was observed with RA followed by T2DM, obesity, AD and MI. Similar to the BP, the degree of similarity in the pathways between psoriasis and its comorbidities was also assessed using Jaccard coefficient (**Fig 9B**). Psoriasis shared the highest number of pathways with obesity followed by RA, MI, T2DM and AD. We noted eight pathways were overlapping with all the comorbidities. Angiogenesis, CCKR signaling map, Gonadotrophin-realising hormone receptor pathway, heterotrimeric G-protein signaling pathway-Gi alpha and Gs alpha mediated pathway, heterotrimeric G-protein signaling pathway-Gq alpha and Go alpha mediated pathway, inflammation mediated by chemokines and cytokine signaling pathway, integrin signaling pathway and Wnt signaling pathway. Toll like receptor signaling pathway was shared by AD, T2DM, MI and RA. Seven pathways involved in blood coagulation, Cadherin signaling, FGF signaling, Ionotropic glutamate receptor pathway, Metabotropic glutamate receptor group III pathway, Muscarinic acetylcholine receptor 1 and 3 signaling pathway, and Nicotinic acetylcholine receptor signaling pathway were enriched in AD,T2DM, obesity and RA. PDGF signaling pathway was found to be overlapping with T2DM, MI, obesity and RA. It was observed that the biological pathways involved in the comorbid diseases were not only shared by psoriasis but also by the other diseases which are almost unrelated to each other except few. These observations shed lights over the existing associations between the psoriasis with its multi-morbidities at the pathway level.

## Discussion

The availability of enormous amount of information from various communities of science makes it essential to appropriately mine out and connect them in a systematic network which allows the creation of new hypothesis. This approach has a probable way to bring about new hypothesis that are not self-evident. In the current work, we adopted an integrative approach by combining the traditional gene expression analysis with the information derived from different databases with the help of bioinformatics.

The network medicine based investigation of five psoriasis comorbidities presented in this work reveals the existence of common genes/ proteins, biological process and pathways. In total, these observations highlight the shared component hypothesis of the psoriasis diseasome leading to the discovery of precise molecular connections between psoriasis and its comorbid diseases. The comorbidities occurring with psoriasis have a major impact in the nature of the disease. Yet the precise mechanisms were not evident. To our knowledge, this is the first study utilizing comprehensive and systematic bioinformatics strategy to investigate the shared component hypothesis [28] as a pathogenic mechanism of psoriasis comorbidities.

The proposed network protocol not only provides a global analysis of the proteins but also presents a detailed view on specific proteins and their association with the comorbidity under the study. We noted the appearance of apolipoprotein E (ApoE) in psoriasis and AD. Lipid metabolism is the common term connecting both the disease. ApoE polymorphisms were shown to be associated with both the diseases disrupting the lipid metabolism. When the direction of regulation was considered the gene was downregulated in both the cases as established by the previous reports [29–30]. An elevated level of MCP-1 (CCL2) is observed between psoriasis and MI. The recruitment of inflammatory mediators by MCP-1 formed a common link connecting both the diseases [31]. Leptin, an important regulator of mass of adipose tissue is found to be downregulated in obesity and psoriasis. Many polymorphisms were reported in leptin in both the disease. The gene alters the endothelial cell morphology and causes epithelial hyperplasia in psoriatic patients [32] while in obesity the alteration in the gene lack the control of metabolic regulation of adipocytes [33]. Early growth respons-1 (Egr-1) is upregulated in T2DM and psoriasis. Egr-1 mediated Egr-1/GGPPS/Erk1/2 pathway is one of the pathways involved in insulin resistance under hyperinsulinism condition observed in T2DM [34]. In psoriasis the protein mediates the expression of psoriasin induced by IL-17A via ERK-Egr-1 pathway. Egr-1 is a crucial modulator in Th17-mediated immune response in epidermal keratinocytes [35]. Upregulation and downregulation of various chemokines (CCL4, CCL18, CCL27, CCR1, CCR5, CCR7, CXCL1, CXCL9, CXCL10, CXCL13 and CXCR4) involved in the recruitment of leukocytes and angiogenesis were observed to occur in both psoriasis and RA linking the immune mediated diseases [36–37].

The findings of our work reveal that the psoriasis comorbidities are related at the molecular level that may contribute to their co-occurrences. In this context, the BP annotation identified several process of inflammation such as defence response and immune response which were enriched in all the comorbidities. The pathway annotation also revealed the presence of inflammatory pathways such as inflammation mediated by chemokines and cytokine signaling pathway, integrin signaling pathway and Wnt signaling pathway. The search for inflammatory links was carried out for all the diseases against published literatures.

The production of amyloid-β peptides can activate the innate immune response and evoke AD. Pattern recognition receptor (PRR) such as C1q is involved in complement cascade activation in the brain. The PPRs were also involved in the induction of pro-inflammatory signaling pathways in AD [38]. Various immune related proteins were produced by adipocytes. Increased expression of peroxisome proliferator activated receptor (PPARγ) was observed in adipose tissue and they are found to be involved in macrophage function [39].Furthermore an adipocyte protein leptin upregulated in obesity and T2DMcan stimulate the activity of macrophage and neutrophil colony-forming cells. The leptin deficiency also imparts impaired lymphoid tissue development. PRRs play major role in inflammation in obesity and T2DM [40]. The ischemic MI was found to activate pleiotropic inflammatory mediators. IL-8 and C5s were released during the injury playing a major role in neutrophil recruitment. Neutrophil infiltrations, activation of complement and cytokine cascades were reported by many researchers as inflammatory reactions in MI [31]. Local production of cytokines accounts for systemic clinical manifestation of RA. The cytokines were the products of IL-1, IL-6, colony stimulating factor (CSF-1), GM-CSF and MCP-1. MCP-1 is chemotactic and plays a role in the recruitment of inflammatory leukocytes into the inflamed joints [36, 41]. The occurrence of BP and pathways related to inflammation might form a basic molecular link connecting psoriasis and its comorbidities.

## Conclusion

Psoriasis and its associated comorbidities highlight psoriasis as a paradigmatic disorder. The comprehensive network based approach investigated the shared component hypothesis existing between psoriasis with its associated co-morbidities. The findings suggest that the most prevalent psoriasis comorbidities were interlinked through common molecular connections which were evidenced by their shared biological functions and pathways. Significant overlap was observed in the biological process and pathways involved in inflammation in most of the comorbidities establishing a link between them. The work identified few associations in the biological processes and molecular pathways which were previously overlooked in majority of the diseases. The current research also shed light on few multimeric novel targets and pathways which can be targeted to offer diagnosis and/or cure for psoriasis along with its associated co-morbidities.

## Supporting Information

S1 Table Differentially expressed genes in psoriasis and its associated comorbidities. (DOCX)

S2 Table Biological process involved in each disease category. The common biological processes between psoriasis and its comorbidities were highlighted.
(DOCX)

S3 Table Biological pathways involved in each disease category. The common biological pathways between psoriasis and its comorbidities are highlighted.
(DOCX)

## References

[1] Perera GK, Di Meglio P, Nestle FO. Psoriasis. Annu Rev Pathol. 2012;7: 385–422. 10.1146/annurev-pathol-011811-132448 22054142

[2] Liu Y, Krueger JG, Bowcock AM. Psoriasis: genetic associations and immune system changes. Genes Immun. 2006;8: 1–12. 10.1038/sj.gene.6364351 17093502

[3] Sticherling M. Mechanisms of psoriasis. Drug Discovery Today: Disease Mechanisms. 2005;2: 275–281. 10.1016/j.ddmec.2005.05.019

[4] Nalls MA, Saad M, Noyce AJ, Keller MF, Schrag A, Bestwick JP, et al Genetic comorbidities in Parkinson’s disease. Hum Mol Genet. 2014;23: 831–841. 10.1093/hmg/ddt465 24057672 PMC3888265

[5] Melamed RD, Emmett KJ, Madubata C, Rzhetsky A, Rabadan R. Genetic similarity between cancers and comorbid Mendelian diseases identifies candidate driver genes. Nat Commun. 2015;6: 7033 10.1038/ncomms8033 25926297 PMC4416231

[6] Gottlieb AB, Chao C, Dann F. Psoriasis comorbidities. J Dermatolog Treat. 2008;19: 5–21. 10.1080/09546630701364768 18273720

[7] Christophers E. Comorbidities in psoriasis. Clin Dermatol. 2007;25: 529–534. 10.1016/j.clindermatol.2007.08.006 18021889

[8] Aurangabadkar S. Comorbidities in psoriasis. Indian Journal of Dermatology, Venereology, and Leprology. 2013;79: 10 10.4103/0378-6323.115506 23974690

[9] Kimball AB, Gladman D, Gelfand JM, Gordon K, Horn EJ, Korman NJ, et al National Psoriasis Foundation clinical consensus on psoriasis comorbidities and recommendations for screening. J Am Acad Dermatol. 2008;58: 1031–1042. 10.1016/j.jaad.2008.01.006 18313171 PMC3716382

[10] Goh K-I, Cusick ME, Valle D, Childs B, Vidal M, Barabási A-L. The human disease network. Proc Natl Acad Sci U S A. 2007;104: 8685–8690. 10.1073/pnas.0701361104 17502601 PMC1885563

[11] Menche J, Sharma A, Kitsak M, Ghiassian SD, Vidal M, Loscalzo J, et al Uncovering disease-disease relationships through the incomplete interactome. Science. 2015;347: 1257601 10.1126/science.1257601 25700523 PMC4435741

[12] Barabási A-L, Gulbahce N, Loscalzo J. Network Medicine: A Network-based Approach to Human Disease. Nat Rev Genet. 2011;12: 56–68. 10.1038/nrg2918 21164525 PMC3140052

[13] Edgar R, Domrachev M, Lash AE. Gene Expression Omnibus: NCBI gene expression and hybridization array data repository. Nucleic Acids Res. 2002;30: 207–210. 11752295 10.1093/nar/30.1.207 PMC99122

[14] Gautier L, Cope L, Bolstad BM, Irizarry RA. affy—analysis of AffymetrixGeneChip data at the probe level. Bioinformatics. 2004;20: 307–315. 10.1093/bioinformatics/btg405 14960456

[15] Ritchie ME, Phipson B, Wu D, Hu Y, Law CW, Shi W, et al limma powers differential expression analyses for RNA-sequencing and microarray studies. Nucl Acids Res. 2015; gkv007 10.1093/nar/gkv007 PMC4402510 25605792

[16] Peri S, Navarro JD, Kristiansen TZ, Amanchy R, Surendranath V, Muthusamy B, et al Human protein reference database as a discovery resource for proteomics. Nucleic Acids Res. 2004;32: D497–501. 10.1093/nar/gkh070 14681466 PMC308804

[17] Pavlidis P, Qin J, Arango V, Mann JJ, Sibille E. Using the gene ontology for microarray data mining: a comparison of methods and application to age effects in human prefrontal cortex. Neurochem Res. 2004;29: 1213–1222. 15176478 10.1023/b:nere.0000023608.29741.45

[18] Huang DW, Sherman BT, Tan Q, Kir J, Liu D, Bryant D, et al DAVID Bioinformatics Resources: expanded annotation database and novel algorithms to better extract biology from large gene lists. Nucleic Acids Res. 2007;35: W169–W175. 10.1093/nar/gkm415 17576678 PMC1933169

[19] Thomas PD, Kejariwal A, Guo N, Mi H, Campbell MJ, Muruganujan A, et al Applications for protein sequence-function evolution data: mRNA/protein expression analysis and coding SNP scoring tools. Nucleic Acids Res. 2006;34: W645–650. 16912992 10.1093/nar/gkl229 PMC1538848

[20] Perez-Llamas C, Lopez-Bigas N. Gitools: Analysis and Visualisation of Genomic Data Using Interactive Heat-Maps. PLoS ONE. 2011;6: e19541 10.1371/journal.pone.0019541 21602921 PMC3094337

[21] Wolf N, Quaranta M, Prescott NJ, Allen M, Smith R, Burden AD, et al Psoriasis is associated with pleiotropic susceptibility loci identified in type II diabetes and Crohn disease. J Med Genet. 2008;45: 114–116. 10.1136/jmg.2007.053595 17993580

[22] McGowan JW, Pearce DJ, Chen J, Richmond D, Balkrishnan R, Feldman SR. The skinny on psoriasis and obesity. Arch Dermatol. 2005;141: 1601–1602. 10.1001/archderm.141.12.1601 16365269

[23] Gelfand JM, Neimann AL, Shin DB, Wang X, Margolis DJ, Troxel AB. Risk of myocardial infarction in patients with psoriasis. JAMA. 2006;296: 1735–1741. 10.1001/jama.296.14.1735 17032986

[24] Bechtel M, Sanders C, Bechtel A. Neurological Complications of Biologic Therapy in Psoriasis. J Clin Aesthet Dermatol. 2009;2: 27–32. 20725577 PMC2923940

[25] Gribble M de G. Rheumatoid Arthritis and Psoriasis. Ann Rheum Dis. 1955;14: 198–207. 14388599 10.1136/ard.14.2.198 PMC1006801

[26] Sitras V, Fenton C, Acharya G. Gene expression profile in cardiovascular disease and preeclampsia: a meta-analysis of the transcriptome based on raw data from human studies deposited in Gene Expression Omnibus. Placenta. 2015;36: 170–178. 10.1016/j.placenta.2014.11.017 25555499

[27] Nazir N, Siddiqui K, Al-Qasim S, Al-Naqeb D. Meta-analysis of diabetic nephropathy associated genetic variants in inflammation and angiogenesis involved in different biochemical pathways. BMC Medical Genetics. 2014;15: 103 10.1186/s12881-014-0103-8 25280384 PMC4411872

[28] Park J, Lee D-S, Christakis NA, Barabási A-L. The impact of cellular networks on disease comorbidity. Mol Syst Biol. 2009;5: 262 10.1038/msb.2009.16 19357641 PMC2683720

[29] Kim J, Basak JM, Holtzman DM. The role of apolipoprotein E in Alzheimer’s disease. Neuron. 2009;63: 287–303. 10.1016/j.neuron.2009.06.026 19679070 PMC3044446

[30] Al Harthi F, Huraib GB, Zouman A, Arfin M, Tariq M, Al-Asmari A. Apolipoprotein E Gene Polymorphism and Serum Lipid Profile in Saudi Patients with Psoriasis. Disease Markers. 2014;2014: e239645 10.1155/2014/239645 PMC3981009 24782577

[31] Kölliker Frers RA, Bisoendial RJ, Montoya SF, Kerzkerg E, Castilla R, Tak PP, et al Psoriasis and cardiovascular risk: Immune-mediated crosstalk between metabolic, vascular and autoimmune inflammation. IJC Metabolic & Endocrine. 2015;6: 43–54. 10.1016/j.ijcme.2015.01.005

[32] Karpouzis A, Tripsianis G, Gatzidou E, Veletza S. Assessment of Leptin Gene Polymorphism rs2060713 in Psoriasis Vulgaris. ISRN Dermatol. 2014;2014: 845272 10.1155/2014/845272 24600521 PMC3926229

[33] Paracchini V, Pedotti P, Taioli E. Genetics of leptin and obesity: a HuGE review. Am J Epidemiol. 2005;162: 101–114. 10.1093/aje/kwi174 15972940

[34] Shen N, Yu X, Pan F-Y, Gao X, Xue B, Li C-J. An early response transcription factor, Egr-1, enhances insulin resistance in type 2 diabetes with chronic hyperinsulinism. J Biol Chem. 2011;286: 14508–14515. 10.1074/jbc.M110.190165 21321112 PMC3077649

[35] Jeong SH, Kim HJ, Jang Y, Ryu WI, Lee H, Kim JH, et al Egr-1 is a key regulator of IL-17A-induced psoriasin upregulation in psoriasis. Exp Dermatol. 2014;23: 890–895. 10.1111/exd.12554 25256120

[36] McInnes IB, Schett G. Cytokines in the pathogenesis of rheumatoid arthritis. Nat Rev Immunol. 2007;7: 429–442. 10.1038/nri2094 17525752

[37] Firestein GS. Evolving concepts of rheumatoid arthritis. Nature. 2003;423: 356–361. 10.1038/nature01661 12748655

[38] Salminen A, Ojala J, Kauppinen A, Kaarniranta K, and Suuronen T. Inflammation in Alzheimer’s disease: Amyloid-β oligomers trigger innate immunity defence via pattern recognition receptors. Progress in Neurobiology. 2009;87: 181–194. 10.1016/j.pneurobio.2009.01.001 19388207

[39] Martí A, Marcos A, Martínez JA. Obesity and immune function relationships. Obes Rev. 2001;2: 131–140. 12119664 10.1046/j.1467-789x.2001.00025.x

[40] Pickup JC. Inflammation and activated innate immunity in the pathogenesis of type 2 diabetes. Diabetes Care. 2004;27: 813–823. 14988310 10.2337/diacare.27.3.813

[41] Koch AE, Kunkel SL, Harlow LA, Mazarakis DD, Haines GK, Burdick MD, et al Macrophage inflammatory protein-1 alpha. A novel chemotactic cytokine for macrophages in rheumatoid arthritis. J Clin Invest. 1994;93: 921–928. 8132778 10.1172/JCI117097 PMC293992

