# Supplementary material for: Comorbidities of Psoriasis - Exploring the Links by Network Approach
Source: PLoS One. 2016 Mar 11;11(3):e0149175. doi: 10.1371/journal.pone.0149175 (PMC4788348; doi:10.1371/journal.pone.0149175)
Supplement: S1 Table — (DOCX) [file pone.0149175.s001.docx]

**S1 Table** : Differentially expressed genes in psoriasis and its associated comorbidities.

| **AD** | | **MI** | | **T2DM** | | **Obesity** | | **RA** | | **Psoriasis** | |
| --- | --- | --- | --- | --- | --- | --- | --- | --- | --- | --- | --- |
| **Gene symbol** | **logFC** | **Gene symbol** | **logFC** | **Gene symbol** | **logFC** | **Gene symbol** | **logFC** | **Gene symbol** | **logFC** | **Gene symbol** | **logFC** |
| ALOX5 | -1.947 | NPPB | 6.248752 | CRISP3 | -3.14409 | LYZ | -4.17442 | PRG4 | -10.3305 | S100A7A | -10.7425 |
| ACTA2 | -1.8331 | CFH | 3.411499 | MAST4 | -2.67679 | PI15 | -3.44664 | MMP3 | -9.64773 | SERPINB4 | -10.5334 |
| FOXC1 | -1.70501 | ID4 | 3.141249 | IFITM1 | -2.60848 | SLC39A8 | -3.32385 | MMP1 | -8.37388 | SPRR2C | -9.52433 |
| MYL9 | -1.60787 | NPPA | 3.006958 | FOSB | -2.49901 | C1S | -3.32329 | SPP1 | -8.17134 | TCN1 | -9.2898 |
| TAGLN | -1.49922 | RPS4Y1 | 2.98071 | KDM4B | -2.47495 | SERPINA3 | -3.26889 | CRTAC1 | -7.75333 | PI3 | -8.75926 |
| CD14 | -1.36791 | PHLDA1 | 2.958078 | ATHL1 | -2.45607 | VCAN | -3.20636 | RGS1 | -7.70803 | S100A12 | -8.62156 |
| LMOD1 | -1.35233 | NOV | 2.780812 | CYP3A5 | -2.43442 | LBP | -3.20191 | FOSB | -7.65479 | AKR1B10 | -7.98754 |
| COX6A1 | -1.34368 | JAK2 | 2.704826 | IRF7 | -2.36291 | RGS4 | -3.17539 | CXCL2 | -7.34659 | TMPRSS11D | -7.04008 |
| APOE | -1.32243 | OMD | 2.690551 | PPFIBP1 | -2.35514 | LEP | -3.12071 | SOCS3 | -7.20367 | ADAMDEC1 | -6.736 |
| NPY | -1.32181 | EIF1AY | 2.587054 | TCIRG1 | -2.33748 | NAMPT | -3.00424 | C5AR1 | -7.19754 | IL8 | -6.71344 |
| CSRP2 | -1.28264 | TNC | 2.575948 | CX3CL1 | -2.24443 | RARRES1 | -2.99988 | IGHM | -7.12062 | LTF | -6.69916 |
| HLA-DPA1 | -1.28258 | FRZB | 2.57295 | SF3A2 | -2.2174 | HP | -2.97778 | SFRP4 | -7.02151 | CHI3L2 | -6.67465 |
| HBE1 | -1.25888 | SULF1 | 2.529257 | AKAP13 | -2.2059 | PTGIS | -2.94323 | SCG2 | -6.96258 | IGFL1 | -6.44061 |
| C1QB | -1.2443 | COMP | 2.507557 | ISG15 | -2.18626 | SPON1 | -2.93714 | CXCL8 | -6.81902 | IL36G | -6.34832 |
| ADIRF | -1.16288 | DDX3Y | 2.505364 | PMEPA1 | -2.16112 | OSMR | -2.92521 | HAS1 | -6.65843 | S100A9 | -6.28933 |
| DDX3Y | -1.11982 | CTGF | 2.464582 | SOX4 | -2.13943 | SEC61A1 | -2.88584 | TREM1 | -6.59947 | OASL | -6.27007 |
| FGF1 | -1.10331 | LOX | 2.402269 | LTB | -2.11698 | STIP1 | -2.88287 | THBS4 | -6.44589 | SERPINB3 | -6.21927 |
| OTOS | -1.09382 | KLHL3 | 2.38571 | POLR1B | -2.11398 | CHI3L1 | -2.81135 | MSR1 | -6.39761 | CCL20 | -6.01455 |
| C3 | 1.093295 | AEBP1 | 2.366408 | TOB2 | -2.10913 | KIAA0101 | -2.79008 | CFI | -6.32129 | KYNU | -5.9857 |
| VEZF1 | -1.09045 | CCL11 | 2.267698 | SECTM1 | -2.09031 | EIF5A | -2.77724 | MS4A7 | -6.2866 | ATP12A | -5.83438 |
| MOG | -1.07649 | LTBP2 | 2.248345 | TMC5 | -2.03586 | PTX3 | -2.76887 | PLA2G2A | -6.21331 | LCN2 | -5.71362 |
| ID4 | -1.07606 | RGS4 | 2.243396 | EGR1 | -2.02762 | ORM1 | -2.75078 | VIM | -6.03256 | IL19 | -5.58712 |
| LDB3 | -1.07213 | CCL2 | 2.234344 | SLPI | -2.02511 | CR1 | -2.73135 | CEMIP | -6.02253 | RHCG | -5.56163 |
| WLS | -1.06874 | ACE2 | 2.153977 | SFN | -2.01331 | PLA2G2A | -2.73058 | CHI3L2 | -5.95897 | C10orf99 | -5.48209 |
| MYH11 | -1.06516 | KDM5D | 2.063099 | UTP18 | 2.001022 | CFB | -2.68991 | FCGR1B | -5.95058 | KLK6 | -5.43676 |
| TPM2 | -1.06466 | COL1A1 | 2.049921 | RSL24D1 | 2.003245 | NEURL2 | -2.65376 | SERPINA1 | -5.85371 | GZMB | -5.3402 |
| EVI2A | -1.06057 | CD14 | -2.11909 | RASA1 | 2.003273 | ENO1 | -2.63653 | ZIC1 | -5.75168 | CXCL1 | -5.326 |
| MAFF | -1.05246 | C1orf105 | -2.1848 | PPP1CC | 2.003427 | CA12 | -2.63482 | C1QB | -5.72729 | MMP12 | -5.26428 |
| MOBP | -1.04678 | B3GALT2 | -2.23195 | ASUN | 2.003888 | ALDH1A3 | -2.62542 | CXCL13 | -5.71715 | HPSE | -5.26339 |
| MECOM | -1.04504 | ATF3 | -2.26837 | METTL5 | 2.004478 | ADAMTS9 | -2.57499 | XIST | -5.66859 | OAS2 | -5.11163 |
| AQP1 | -1.0401 | FCN3 | -2.40709 | WIPI1 | 2.004925 | CXCL6 | -2.56899 | SERPINE1 | -5.57819 | KRT16 | -5.07504 |
| CRTAC1 | -1.03213 | XIST | -2.57883 | PDHX | 2.0052 | HSP90AB1 | -2.56263 | SIX1 | -5.54505 | CCL18 | -5.06433 |
| LGALS1 | -1.03123 | ETNPPL | -3.2609 | ATR | 2.005592 | SLPI | -2.55374 | CCL18 | -5.52806 | CXCL13 | -4.91874 |
| LZIC | -1.02841 | S100A8 | -3.45087 | GSTA4 | 2.00616 | RRM2 | -2.54702 | CSN1S1 | -5.49997 | LCE3D | -4.87284 |
| AEBP1 | -1.02836 | CORIN | -3.77035 | ASNA1 | 2.010233 | TFRC | -2.52368 | BCAT1 | -5.47891 | S100A7 | -4.67805 |
| ANTXR1 | -1.02529 | RARRES1 | -4.46603 | RHOBTB3 | 2.013433 | CTNNA1 | -2.522 | IGKC | -5.44251 | RSAD2 | -4.63128 |
| AQP4 | -1.02396 |  |  | DHCR7 | 2.016718 | ZWINT | -2.51852 | COMP | -5.43126 | KLK13 | -4.59178 |
| SERPINB1 | -1.02078 |  |  | CHGA | 2.017489 | PLAU | -2.48435 | MARCO | -5.43003 | PRSS27 | -4.58688 |
| BGN | -1.00773 |  |  | RHOQ | 2.017523 | SCD | -2.48215 | ANXA1 | -5.37269 | CXCL10 | -4.57995 |
| LIX1 | -1.0074 |  |  | CXorf57 | 2.019131 | GREM1 | -2.46243 | TGM2 | -5.35068 | CHAC1 | -4.56113 |
| ITPRIPL2 | -1.00505 |  |  | EIF4E | 2.019673 | HLA-DQB1 | -2.45444 | FCGR3B | -5.33736 | EPSTI1 | -4.46172 |
| ATP6V0E1 | -1.0038 |  |  | ATP2A2 | 2.020238 | IL24 | -2.45231 | TIMD4 | -5.33032 | CHRNA9 | -4.43229 |
| EZR | -1.00248 |  |  | ANKRD46 | 2.020944 | FAM20A | -2.45149 | ST8SIA4 | -5.32392 | ARSF | -4.397 |
| UNC80 | 1.000219 |  |  | TFB2M | 2.0215 | UBA6 | -2.43344 | SLC16A3 | -5.28243 | TYMP | -4.36819 |
| OXCT1 | 1.00045 |  |  | COPB2 | 2.022741 | ATP6V1A | -2.40704 | CFB | -5.27849 | IL36A | -4.35238 |
| RAPGEF2 | 1.00151 |  |  | TTC19 | 2.024539 | PAPPA | -2.39724 | IGLC1 | -5.27062 | FUT2 | -4.33635 |
| CSMD3 | 1.003027 |  |  | NME1 | 2.025343 | NAIP | -2.39529 | TNFRSF11B | -5.25 | UPP1 | -4.32979 |
| FZD7 | 1.003726 |  |  | C6orf211 | 2.025654 | PDPN | -2.3825 | CD69 | -5.19839 | HYAL4 | -4.30743 |
| HS6ST3 | 1.005414 |  |  | SWAP70 | 2.027094 | CXCL1 | -2.37701 | SLC2A3 | -5.16018 | PRSS53 | -4.30498 |
| ZNF529 | 1.006596 |  |  | JOSD1 | 2.027742 | CCL20 | -2.37155 | OGN | -5.11911 | ZC3H12A | -4.30165 |
| LMBRD2 | 1.014296 |  |  | IPO7 | 2.029543 | PLTP | -2.36512 | TMEM196 | -5.09514 | RTP4 | -4.28694 |
| ACTN1 | 1.015824 |  |  | MCFD2 | 2.031154 | DNAJC3 | -2.35805 | GPR64 | -5.08986 | MPZL2 | -4.22239 |
| SCN8A | 1.016177 |  |  | APLP2 | 2.032017 | CCL18 | -2.35556 | EPHA3 | -5.04557 | PGBD5 | -4.21881 |
| PPFIBP1 | 1.017269 |  |  | ZNHIT6 | 2.032042 | P4HB | -2.34423 | CTSS | -5.04194 | CXCL9 | -4.18693 |
| BBS7 | 1.01776 |  |  | MFF | 2.033671 | CFI | -2.33219 | MS4A4A | -5.02177 | FOXE1 | -4.18622 |
| KIAA1549L | 1.018553 |  |  | NEUROD1 | 2.034285 | CCL23 | -2.30177 | LILRB2 | -5.01241 | SLC6A14 | -4.15891 |
| TLN1 | 1.018869 |  |  | STAM | 2.035326 | SERPINA1 | -2.27175 | KYNU | -4.9968 | CXCR4 | -4.12843 |
| COL4A3BP | 1.019178 |  |  | ELF1 | 2.036571 | WT1 | -2.23358 | LILRB4 | -4.99481 | KCNJ15 | -4.07458 |
| CACNA2D1 | 1.024864 |  |  | SRSF7 | 2.037797 | LMAN1 | -2.23187 | SNED1 | -4.95341 | IFI27 | -4.06686 |
| FAM126B | 1.027111 |  |  | BTBD3 | 2.039418 | CCL19 | -2.23039 | ARSB | -4.92254 | LMNB2 | -4.065 |
| ABCC5 | 1.027305 |  |  | RHEB | 2.039801 | FAS | -2.23031 | PTPRD | -4.91729 | HS3ST3A1 | -4.02617 |
| FAM150B | 1.029068 |  |  | CMAS | 2.03988 | ADAM9 | -2.22838 | FPR3 | -4.91585 | TREX2 | -3.94226 |
| PPL | 1.030296 |  |  | RAB14 | 2.040114 | CADM3 | -2.21961 | SCRG1 | -4.91521 | DLGAP5 | -3.92608 |
| CTXN3 | 1.034499 |  |  | SAR1A | 2.041424 | ANLN | -2.2018 | PTPN22 | -4.90746 | CCNA2 | -3.89397 |
| HMGCR | 1.035745 |  |  | SUMO1 | 2.043244 | BCAT1 | -2.18755 | TMEFF2 | -4.90526 | CDH26 | -3.88486 |
| ENC1 | 1.037434 |  |  | GPRASP1 | 2.045153 | C7 | -2.15378 | TFEC | -4.90411 | FCHSD1 | -3.85576 |
| HS6ST2 | 1.041397 |  |  | COA1 | 2.047409 | LYVE1 | -2.15168 | HTRA4 | -4.86715 | CCNB1 | -3.84871 |
| NUP133 | 1.043956 |  |  | FZD5 | 2.048973 | PI3 | -2.14227 | CXCL1 | -4.85146 | S100A8 | -3.8003 |
| SERPINI1 | 1.044825 |  |  | H2AFZ | 2.049379 | TNFSF14 | -2.13632 | FPR1 | -4.82367 | APOBEC3A | -3.77564 |
| NCOA2 | 1.047691 |  |  | RAB2A | 2.049394 | TIMP1 | -2.13495 | VCAM1 | -4.81324 | MXD1 | -3.77068 |
| TM2D3 | 1.049293 |  |  | ASF1A | 2.050586 | CLIC4 | -2.12716 | FNDC1 | -4.80012 | SERPINA1 | -3.74258 |
| RFX3 | 1.050037 |  |  | TAF9 | 2.053809 | SNAP23 | -2.11346 | TLR8 | -4.76548 | KLK9 | -3.72653 |
| PPP3CA | 1.050938 |  |  | RCN2 | 2.054548 | CAB39 | -2.09702 | CXCL3 | -4.76296 | DEPDC1B | -3.70676 |
| PCDH8 | 1.051179 |  |  | CDK4 | 2.054827 | CYP1B1 | -2.09439 | CYBB | -4.75786 | KLHL18 | -3.70242 |
| ADAM22 | 1.05257 |  |  | CD200 | 2.054957 | SHC1 | -2.07391 | TLR4 | -4.72785 | SPC25 | -3.67606 |
| XIST | 1.05688 |  |  | ADK | 2.056634 | TMED2 | -2.06826 | C1QA | -4.72064 | SAMD9 | -3.67384 |
| NLGN4X | 1.058413 |  |  | BCL2L2 | 2.058385 | CALU | -2.0468 | GFPT2 | -4.6936 | SLAMF7 | -3.63196 |
| SIPA1L1 | 1.061027 |  |  | HSPE1 | 2.060718 | COL12A1 | -2.03425 | ZNF385B | -4.69157 | SERPINB13 | -3.62387 |
| SFPQ | 1.063882 |  |  | H2AFV | 2.066158 | PPIF | -2.02919 | CXCR4 | -4.68306 | SH3PXD2A-AS1 | -3.61351 |
| TUNAR | 1.064364 |  |  | SLC30A1 | 2.066404 | LAPTM4B | -2.02732 | FNDC3B | -4.67786 | KLRB1 | -3.60314 |
| VPS13A | 1.07659 |  |  | C11orf58 | 2.067808 | SRSF1 | -2.02679 | NAMPT | -4.67223 | CYP7B1 | -3.5895 |
| GRIN2A | 1.077059 |  |  | MAP1B | 2.068806 | KRT18 | -2.02226 | SLC16A4 | -4.64841 | INA | -3.57468 |
| HBS1L | 1.077736 |  |  | GSPT1 | 2.069133 | TDO2 | -2.00291 | THEMIS2 | -4.62976 | SLC26A9 | -3.57175 |
| ATP6V1C1 | 1.080222 |  |  | SF3B1 | 2.07002 | PROK2 | -2.00035 | MAB21L2 | -4.61258 | SPRR2G | -3.57123 |
| TMEM163 | 1.083088 |  |  | CDC40 | 2.070022 | CASQ2 | 2.006626 | CLIC5 | -4.60873 | BCL2A1 | -3.5678 |
| TMEM108 | 1.088219 |  |  | ID4 | 2.076017 | ITIH5 | 2.01684 | CTSZ | -4.56846 | NAMPT | -3.56115 |
| TMEM158 | 1.107629 |  |  | PPP2CB | 2.081267 | CA4 | 2.020862 | LBP | -4.564 | PRKCQ | -3.53834 |
| NRXN1 | 1.110129 |  |  | SYNCRIP | 2.081663 | CAB39L | 2.024205 | SULF1 | -4.55264 | OAS1 | -3.52295 |
| PAK6 | 1.119361 |  |  | FNDC3A | 2.0827 | LIMCH1 | 2.025224 | KLHL6 | -4.52505 | UBE2T | -3.48571 |
| MFSD4 | 1.122856 |  |  | DPP8 | 2.084953 | NUCKS1 | 2.057132 | SLCO2B1 | -4.51418 | CCDC19 | -3.48229 |
| ATAD2B | 1.131011 |  |  | HPRT1 | 2.085444 | PPP1R14A | 2.060346 | APOL1 | -4.50286 | ZBED2 | -3.44855 |
| SYNDIG1L | 1.133725 |  |  | POP4 | 2.086573 | WISP2 | 2.060669 | CR1 | -4.49607 | MMP1 | -3.44216 |
| GRIA2 | 1.137122 |  |  | ENDOD1 | 2.087971 | NTRK2 | 2.08046 | Mar-01 | -4.49308 | GJB2 | -3.43959 |
| RASGRP1 | 1.141962 |  |  | USO1 | 2.089195 | FAM26F | 2.090949 | LAIR1 | -4.45781 | CNNM4 | -3.43891 |
| SEMA5A | 1.14617 |  |  | CTR9 | 2.091441 | CDKN2B | 2.097745 | RARRES1 | -4.44087 | SELL | -3.41559 |
| LINC01128 | 1.151095 |  |  | NPTN | 2.091932 | HERC6 | 2.114321 | TLR7 | -4.43939 | CHAC2 | -3.39599 |
| CHL1 | 1.155237 |  |  | PLS1 | 2.092523 | PCDH9 | 2.116959 | VSIG4 | -4.38004 | SPRR3 | -3.37805 |
| DNM3 | 1.157765 |  |  | FAM8A1 | 2.093903 | BTNL9 | 2.123477 | CCL4 | -4.37368 | GALNT6 | -3.37519 |
| CDK5R1 | 1.176043 |  |  | CAPZA2 | 2.094779 | LOC100288911 | 2.128236 | TNFAIP6 | -4.35086 | TGM1 | -3.35339 |
| RALGAPA2 | 1.187978 |  |  | SNW1 | 2.101667 | NPY1R | 2.132538 | IFI44L | -4.34575 | SAMSN1 | -3.33896 |
| LPPR4 | 1.188887 |  |  | CLK1 | 2.102762 | C2orf40 | 2.141631 | CCL8 | -4.33892 | GK | -3.33481 |
| AACS | 1.194994 |  |  | INTS12 | 2.103543 | ZBTB16 | 2.14258 | IGLJ3 | -4.33614 | DSC2 | -3.31539 |
| LPCAT4 | 1.198312 |  |  | TSPAN12 | 2.103986 | EGR1 | 2.14398 | LOC100132891 | -4.31951 | GBP6 | -3.30255 |
| ADRA2A | 1.210224 |  |  | AP3B1 | 2.104502 | GBP1 | 2.146062 | CCR1 | -4.31179 | LAIR2 | -3.30241 |
| ZNF204P | 1.224804 |  |  | MAP4K3 | 2.105003 | MYOC | 2.14845 | SLC38A6 | -4.30546 | SOX7 | -3.29851 |
| CARTPT | 1.232681 |  |  | FBXO9 | 2.105694 | DEFB132 | 2.174429 | ADAM12 | -4.29749 | GPX2 | -3.26357 |
| LZTS1 | 1.23869 |  |  | FBXO3 | 2.106028 | OR51E1 | 2.209495 | GAP43 | -4.25848 | MELK | -3.24673 |
| AKAP5 | 1.267248 |  |  | SDHC | 2.108342 | LOC100653086 | 2.214167 | DOK3 | -4.25703 | FUT3 | -3.22623 |
| NTNG1 | 1.276676 |  |  | KLHL41 | 2.115406 | GSTT1 | 2.223777 | PCSK1 | -4.2487 | HSD17B2 | -3.20148 |
| MCOLN3 | 1.285268 |  |  | RAB21 | 2.116179 | CMPK2 | 2.235041 | FGF10 | -4.24841 | OR7E12P | -3.1999 |
| CNTN5 | 1.289157 |  |  | AASDHPPT | 2.117395 | NTM | 2.243121 | COL8A1 | -4.24279 | CEP55 | -3.19853 |
| FABP7 | 1.296809 |  |  | VPS13C | 2.118254 | DPT | 2.247879 | CD163 | -4.24123 | KIF20A | -3.18993 |
| PDP1 | 1.309026 |  |  | ZNF226 | 2.118864 | PDK4 | 2.256797 | FOS | -4.21198 | SLC26A4 | -3.15801 |
| ROCK2 | 1.319485 |  |  | SEL1L | 2.119113 | CRNDE | 2.286725 | BCL2A1 | -4.20526 | TNIP3 | -3.14722 |
| LMO4 | 1.329433 |  |  | RPLP0 | 2.120118 | SIM1 | 2.289079 | SELL | -4.19868 | NETO2 | -3.13277 |
| PCDH20 | 1.330194 |  |  | DNAJC6 | 2.123435 | ADAMTS18 | 2.31628 | EIF1 | -4.19402 | DENND1A | -3.121 |
| CA12 | 1.333826 |  |  | ENPP4 | 2.124076 | ALDH4A1 | 2.424443 | SAMSN1 | -4.18956 | ISG15 | -3.11114 |
| ETNK1 | 1.364191 |  |  | TOM1L1 | 2.125776 | GLDN | 2.433009 | CXCL9 | -4.18175 | VMP1 | -3.0974 |
| GABRB3 | 1.374347 |  |  | DNAJC10 | 2.126541 | RSAD2 | 2.475149 | C3AR1 | -4.17461 | CD24 | -3.08707 |
| CNKSR2 | 1.388647 |  |  | CRK | 2.127944 | BHMT2 | 2.514031 | EGR1 | -4.15993 | PLA2G2F | -3.06673 |
| CASD1 | 1.394244 |  |  | TMEM50A | 2.12826 | CCDC3 | 2.601039 | CTSLP8 | -4.13517 | ZIC1 | -3.05363 |
| ANO3 | 1.394985 |  |  | MTX2 | 2.129549 | MTURN | 2.629696 | IL6 | -4.13442 | WNT5A | -3.0531 |
| TGFBI | 1.404832 |  |  | IFNGR1 | 2.129749 | SCUBE2 | 2.701101 | CYP1B1 | -4.1326 | TTC39A | -3.04998 |
| CBLN4 | 1.458998 |  |  | SSBP1 | 2.130002 | XAF1 | 2.802134 | KCNE4 | -4.09799 | GK3P | -3.00936 |
| SLC2A13 | 1.46323 |  |  | PGM3 | 2.130805 | FOSB | 2.919299 | FAP | -4.09581 | KIF2C | -3.00331 |
| EGR4 | 1.468107 |  |  | HSPA14 | 2.134214 | NQO1 | 2.934432 | RGS16 | -4.08757 | APOE | -3.00133 |
| COL5A2 | 1.48419 |  |  | XPOT | 2.134525 | SFRP1 | 3.028611 | C1QC | -4.07642 | ENTPD7 | -3.00055 |
| PLK2 | 1.552852 |  |  | SCGB2A1 | 2.136143 | IDO1 | 3.038919 | DMXL2 | -4.0738 | SPC24 | -2.99939 |
| CDH8 | 1.707472 |  |  | GLRX3 | 2.137238 | NPR3 | 3.21817 | GPR183 | -4.04978 | RGS1 | -2.99894 |
| OPRK1 | 1.773406 |  |  | ZZZ3 | 2.138429 | TNMD | 3.591914 | C2 | -4.04661 | CFB | -2.9807 |
| CUX2 | 2.023351 |  |  | ADSS | 2.140378 | CYP4B1 | 3.695585 | IGLV1-44 | -4.04068 | RRM2 | -2.96941 |
|  |  |  |  | HS2ST1 | 2.141093 |  |  | DLX4 | -4.03805 | CD163L1 | -2.95158 |
|  |  |  |  | EMC2 | 2.142901 |  |  | CD84 | -4.03015 | CLEC7A | -2.9193 |
|  |  |  |  | AADAC | 2.149664 |  |  | NOX4 | -4.02713 | KIF23 | -2.91703 |
|  |  |  |  | IARS | 2.15032 |  |  | OAS2 | -4.02649 | CCL22 | -2.91372 |
|  |  |  |  | TOX3 | 2.153403 |  |  | IGHD | -4.02273 | GDPD3 | -2.90815 |
|  |  |  |  | TFPI | 2.153471 |  |  | SPIDR | -4.01872 | TGM3 | -2.90643 |
|  |  |  |  | ZFAND1 | 2.153605 |  |  | COL14A1 | -3.98537 | CSF2RA | -2.90174 |
|  |  |  |  | STRAP | 2.159061 |  |  | FCGR2A | -3.98142 | CCRN4L | -2.89524 |
|  |  |  |  | OAZ1 | 2.159188 |  |  | LINC01094 | -3.96624 | ST6GALNAC1 | -2.89508 |
|  |  |  |  | UBE2E3 | 2.15995 |  |  | ANKRD28 | -3.96313 | OAS3 | -2.88996 |
|  |  |  |  | PRDX4 | 2.163742 |  |  | EPYC | -3.96199 | SLC5A1 | -2.88292 |
|  |  |  |  | NBEA | 2.163833 |  |  | CXCL10 | -3.95687 | BUB1 | -2.88002 |
|  |  |  |  | CAPRIN1 | 2.164783 |  |  | HK3 | -3.95344 | KIAA0101 | -2.86723 |
|  |  |  |  | NKX2-2 | 2.164862 |  |  | EBF2 | -3.93454 | PDZK1IP1 | -2.86204 |
|  |  |  |  | RANBP2 | 2.165012 |  |  | IGLV@ | -3.92858 | FCGR1B | -2.86022 |
|  |  |  |  | TM9SF2 | 2.168158 |  |  | SRGN | -3.92492 | RAB27A | -2.8601 |
|  |  |  |  | LAPTM4B | 2.168296 |  |  | LILRB5 | -3.92062 | TYMS | -2.86006 |
|  |  |  |  | NAMPT | 2.168759 |  |  | PLAUR | -3.91838 | EHF | -2.85869 |
|  |  |  |  | ARPC5 | 2.169176 |  |  | NDP | -3.90988 | PARPBP | -2.85661 |
|  |  |  |  | SH3GL2 | 2.170415 |  |  | PNMA2 | -3.88537 | GM2A | -2.85275 |
|  |  |  |  | DERL1 | 2.17108 |  |  | VMP1 | -3.87365 | MCM10 | -2.83606 |
|  |  |  |  | MLLT11 | 2.171652 |  |  | POU2AF1 | -3.87206 | MAD2L1 | -2.83042 |
|  |  |  |  | ENOPH1 | 2.171751 |  |  | WISP1 | -3.86807 | EPHB2 | -2.82243 |
|  |  |  |  | M6PR | 2.171958 |  |  | FAM20A | -3.84904 | IL12RB1 | -2.82111 |
|  |  |  |  | KIDINS220 | 2.176501 |  |  | ZFHX4 | -3.81328 | AIM2 | -2.81191 |
|  |  |  |  | HNRNPA0 | 2.176504 |  |  | IGF1 | -3.78346 | GJB6 | -2.81092 |
|  |  |  |  | TXNDC9 | 2.178417 |  |  | CCNL1 | -3.77711 | IFI6 | -2.80581 |
|  |  |  |  | PCBP1 | 2.178511 |  |  | ITGB2 | -3.77684 | CD274 | -2.80531 |
|  |  |  |  | RNF138 | 2.179193 |  |  | NCEH1 | -3.77531 | FAIM3 | -2.79723 |
|  |  |  |  | GUCY1B3 | 2.180521 |  |  | OLR1 | -3.77042 | CDKN3 | -2.79569 |
|  |  |  |  | FAM208A | 2.182126 |  |  | HBEGF | -3.76473 | POLR3G | -2.79226 |
|  |  |  |  | HLTF | 2.184414 |  |  | KIAA1462 | -3.75018 | CASP5 | -2.78529 |
|  |  |  |  | PSMC3 | 2.184922 |  |  | CXCL6 | -3.74743 | PTTG3P | -2.7758 |
|  |  |  |  | FUCA1 | 2.185125 |  |  | DLEU2 | -3.74402 | SPRR1B | -2.77259 |
|  |  |  |  | PPP3CA | 2.187057 |  |  | NID2 | -3.73811 | KRT6A | -2.76963 |
|  |  |  |  | CACNA1D | 2.187772 |  |  | PMP22 | -3.71006 | IL1B | -2.7575 |
|  |  |  |  | ETV1 | 2.187941 |  |  | NR4A2 | -3.70506 | TK1 | -2.75578 |
|  |  |  |  | TGFBR2 | 2.194459 |  |  | EPSTI1 | -3.69192 | GZMA | -2.7529 |
|  |  |  |  | PIK3R4 | 2.199691 |  |  | SIGLEC1 | -3.68475 | GDA | -2.75279 |
|  |  |  |  | CDC16 | 2.203319 |  |  | SMOC1 | -3.68229 | TPRXL | -2.75116 |
|  |  |  |  | GCH1 | 2.203983 |  |  | CD28 | -3.67683 | SERPINB1 | -2.74651 |
|  |  |  |  | SDC2 | 2.205984 |  |  | FKBP10 | -3.67303 | C1orf43 | -2.74359 |
|  |  |  |  | SERPINI1 | 2.206751 |  |  | ST3GAL1 | -3.66842 | VNN1 | -2.74182 |
|  |  |  |  | BEX1 | 2.207097 |  |  | SLC2A5 | -3.65722 | SELE | -2.73968 |
|  |  |  |  | SH3BGRL | 2.207584 |  |  | FCGR2C | -3.64805 | CRABP2 | -2.72495 |
|  |  |  |  | CCNG1 | 2.214542 |  |  | PDE10A | -3.6471 | MX1 | -2.71989 |
|  |  |  |  | ABI1 | 2.214654 |  |  | TUBB2B | -3.64468 | CCL4 | -2.71923 |
|  |  |  |  | SPTSSA | 2.219654 |  |  | RNF213 | -3.64169 | C12orf56 | -2.71311 |
|  |  |  |  | C14orf132 | 2.219862 |  |  | BMS1P20 | -3.64038 | LAMP3 | -2.70811 |
|  |  |  |  | DCK | 2.22051 |  |  | EMP1 | -3.63875 | UHRF1 | -2.70744 |
|  |  |  |  | HSPA13 | 2.222535 |  |  | ANKRD44 | -3.6299 | ESYT3 | -2.69999 |
|  |  |  |  | PRKD3 | 2.225835 |  |  | CEBPD | -3.61462 | DEPDC1 | -2.69439 |
|  |  |  |  | NRP1 | 2.23363 |  |  | NT5E | -3.6085 | PNP | -2.69405 |
|  |  |  |  | ACVR1 | 2.237877 |  |  | RASSF4 | -3.59544 | PARP9 | -2.69207 |
|  |  |  |  | PTP4A1 | 2.238983 |  |  | PLOD2 | -3.59353 | CXCR2 | -2.68606 |
|  |  |  |  | NECAP1 | 2.239036 |  |  | ADAMDEC1 | -3.58752 | CDC20 | -2.68502 |
|  |  |  |  | PGRMC2 | 2.240681 |  |  | KIAA0226L | -3.58668 | TTC9 | -2.68391 |
|  |  |  |  | PAPOLA | 2.241546 |  |  | ADAMTS1 | -3.58368 | TTK | -2.67291 |
|  |  |  |  | PAPSS1 | 2.242134 |  |  | SELE | -3.57646 | MMP9 | -2.67213 |
|  |  |  |  | IMPAD1 | 2.242655 |  |  | LY96 | -3.56657 | FOSL1 | -2.66838 |
|  |  |  |  | POLE3 | 2.242753 |  |  | BGN | -3.55989 | HJURP | -2.66507 |
|  |  |  |  | SLMO2 | 2.243725 |  |  | FKBP11 | -3.55776 | IDO1 | -2.65996 |
|  |  |  |  | TASP1 | 2.244888 |  |  | ANGPT1 | -3.55674 | RDH16 | -2.65968 |
|  |  |  |  | BMP5 | 2.246053 |  |  | ALDH1A3 | -3.5526 | NCAPH | -2.65888 |
|  |  |  |  | PJA2 | 2.247237 |  |  | MT1M | -3.55044 | LRP8 | -2.65742 |
|  |  |  |  | WDR61 | 2.247558 |  |  | PLEK | -3.54569 | PRKCQ-AS1 | -2.65446 |
|  |  |  |  | HSPD1 | 2.248415 |  |  | SLC11A1 | -3.54396 | IL36RN | -2.64949 |
|  |  |  |  | TRMT11 | 2.249954 |  |  | LINC01139 | -3.52995 | IL7R | -2.64462 |
|  |  |  |  | FBXL5 | 2.250116 |  |  | P4HA3 | -3.5277 | LCK | -2.6444 |
|  |  |  |  | KLHDC2 | 2.250284 |  |  | LILRB1 | -3.52697 | SPAG5 | -2.64153 |
|  |  |  |  | SPCS2 | 2.25354 |  |  | TBX5 | -3.51808 | CMPK2 | -2.63844 |
|  |  |  |  | LAMP2 | 2.254376 |  |  | STEAP1 | -3.51664 | NEK2 | -2.6346 |
|  |  |  |  | SLC9A6 | 2.255549 |  |  | FCGR2B | -3.51651 | KIF14 | -2.6325 |
|  |  |  |  | ACPP | 2.257448 |  |  | IL13RA2 | -3.51013 | LIPG | -2.62999 |
|  |  |  |  | IGF2 | 2.258724 |  |  | CD14 | -3.50337 | P2RY2 | -2.62611 |
|  |  |  |  | TMEM126B | 2.262424 |  |  | IGSF6 | -3.50168 | ALDH1A3 | -2.62523 |
|  |  |  |  | G3BP2 | 2.269002 |  |  | CALCRL | -3.49 | SHCBP1 | -2.62231 |
|  |  |  |  | NAE1 | 2.272526 |  |  | TNC | -3.48557 | SLC16A1 | -2.62075 |
|  |  |  |  | VLDLR | 2.273894 |  |  | FILIP1 | -3.48345 | CDK5R1 | -2.61239 |
|  |  |  |  | ETFA | 2.275847 |  |  | EPB41L3 | -3.47815 | PBK | -2.608 |
|  |  |  |  | INA | 2.280163 |  |  | REV3L | -3.45738 | AURKB | -2.60614 |
|  |  |  |  | PAM | 2.282833 |  |  | ND6 | -3.45239 | CDC6 | -2.60418 |
|  |  |  |  | TTR | 2.283521 |  |  | HGF | -3.44906 | PGLYRP4 | -2.59804 |
|  |  |  |  | C5orf28 | 2.284667 |  |  | MMP19 | -3.44239 | PKMYT1 | -2.59427 |
|  |  |  |  | SLC39A6 | 2.285518 |  |  | C1orf162 | -3.43695 | PCP4L1 | -2.58794 |
|  |  |  |  | GPR64 | 2.286408 |  |  | DOK2 | -3.43608 | ARL11 | -2.57909 |
|  |  |  |  | PSMD6 | 2.28666 |  |  | ARRB2 | -3.43132 | SLC25A10 | -2.57463 |
|  |  |  |  | RNF13 | 2.287897 |  |  | ANKRD36BP2 | -3.42878 | IFI44L | -2.5721 |
|  |  |  |  | ARPC2 | 2.290409 |  |  | CRISPLD1 | -3.42674 | ALOX12B | -2.56242 |
|  |  |  |  | USP1 | 2.299631 |  |  | BEND6 | -3.42558 | KLK10 | -2.55201 |
|  |  |  |  | SNX2 | 2.300057 |  |  | HAVCR2 | -3.42554 | LEP | -2.54856 |
|  |  |  |  | SQLE | 2.301316 |  |  | CDO1 | -3.42534 | TMPRSS4 | -2.54797 |
|  |  |  |  | GCG | 2.302022 |  |  | WIPF1 | -3.4151 | CDCA2 | -2.54753 |
|  |  |  |  | SEC63 | 2.302298 |  |  | SNX10 | -3.39357 | CKS2 | -2.54011 |
|  |  |  |  | IDS | 2.306559 |  |  | KCNJ15 | -3.39164 | TGFA | -2.53544 |
|  |  |  |  | PGD | 2.30848 |  |  | SAMD9L | -3.39048 | SMOX | -2.53473 |
|  |  |  |  | TSPYL5 | 2.310759 |  |  | ENPP1 | -3.39002 | IFIT3 | -2.52702 |
|  |  |  |  | CAT | 2.311433 |  |  | IGHG1 | -3.38457 | SMPD3 | -2.52568 |
|  |  |  |  | SDHB | 2.311743 |  |  | FMO3 | -3.38292 | IRF7 | -2.52546 |
|  |  |  |  | EMC7 | 2.313381 |  |  | ANGPTL2 | -3.37422 | CENPL | -2.52235 |
|  |  |  |  | GOLGA7 | 2.319217 |  |  | BST2 | -3.37253 | IL20 | -2.51975 |
|  |  |  |  | ATF1 | 2.319672 |  |  | SLC7A7 | -3.3633 | GPR65 | -2.51912 |
|  |  |  |  | NME7 | 2.322486 |  |  | BNC2 | -3.35928 | TMEM45B | -2.51126 |
|  |  |  |  | YES1 | 2.32315 |  |  | S1PR3 | -3.3579 | NFKBIZ | -2.50947 |
|  |  |  |  | RABGGTB | 2.33081 |  |  | SIX3 | -3.35158 | ISG20 | -2.50732 |
|  |  |  |  | ACTR2 | 2.332416 |  |  | NNMT | -3.33474 | DSG3 | -2.50532 |
|  |  |  |  | TPRKB | 2.335622 |  |  | PLXDC1 | -3.33215 | GINS3 | -2.49696 |
|  |  |  |  | TMED10 | 2.336578 |  |  | B4GALT1 | -3.32765 | NCAPG | -2.48515 |
|  |  |  |  | NRCAM | 2.3373 |  |  | GLIS3 | -3.32288 | TRIP13 | -2.4824 |
|  |  |  |  | LIMCH1 | 2.341214 |  |  | ACKR3 | -3.31842 | DDX58 | -2.47775 |
|  |  |  |  | NEFM | 2.350285 |  |  | PDE1A | -3.31425 | KRT6B | -2.47683 |
|  |  |  |  | ATP6V1A | 2.35033 |  |  | FAM49A | -3.31162 | SPRR1A | -2.47681 |
|  |  |  |  | EPB41L3 | 2.354552 |  |  | BTBD19 | -3.30437 | SLC7A5 | -2.46679 |
|  |  |  |  | SCD | 2.356227 |  |  | SIRPB2 | -3.29653 | PRSS2 | -2.46556 |
|  |  |  |  | VDAC3 | 2.357949 |  |  | MS4A1 | -3.29124 | SH2D1A | -2.46129 |
|  |  |  |  | PLCB4 | 2.360881 |  |  | GOLM1 | -3.29031 | LOC100996579 | -2.45278 |
|  |  |  |  | CNBP | 2.364603 |  |  | PLAU | -3.27877 | STXBP2 | -2.44824 |
|  |  |  |  | SNX7 | 2.366722 |  |  | ASPN | -3.27524 | AURKA | -2.44065 |
|  |  |  |  | INPP5F | 2.369713 |  |  | FYB | -3.27323 | ABCG4 | -2.43912 |
|  |  |  |  | CPD | 2.371264 |  |  | RSAD2 | -3.2688 | HAPLN3 | -2.43888 |
|  |  |  |  | ZNF277 | 2.376188 |  |  | TYROBP | -3.26546 | NUF2 | -2.43353 |
|  |  |  |  | ECI2 | 2.381396 |  |  | ZMYM5 | -3.26506 | CHEK1 | -2.43156 |
|  |  |  |  | GLT8D1 | 2.386319 |  |  | APBB1IP | -3.25577 | SPTLC2 | -2.42324 |
|  |  |  |  | WNT4 | 2.387724 |  |  | HEG1 | -3.25407 | FAM83A | -2.42222 |
|  |  |  |  | BTG3 | 2.392218 |  |  | IFI44 | -3.25352 | A2ML1 | -2.41497 |
|  |  |  |  | HMGN4 | 2.398829 |  |  | STAB1 | -3.25285 | ASPM | -2.41488 |
|  |  |  |  | SC5D | 2.399135 |  |  | HP | -3.25165 | BAK1 | -2.39666 |
|  |  |  |  | DESI2 | 2.41007 |  |  | TM6SF1 | -3.25026 | GBP5 | -2.38266 |
|  |  |  |  | CNIH1 | 2.412357 |  |  | ERAP2 | -3.24413 | LOC100506013 | -2.38127 |
|  |  |  |  | KCTD3 | 2.414639 |  |  | CILP2 | -3.22658 | CACNB4 | -2.37736 |
|  |  |  |  | PTGES3 | 2.418227 |  |  | OAS1 | -3.22519 | LOC100506100 | -2.36167 |
|  |  |  |  | TMEM14B | 2.421188 |  |  | PILRA | -3.21947 | LOC100131262 | -2.36142 |
|  |  |  |  | ATP6V1B2 | 2.424571 |  |  | RP2 | -3.2145 | HMMR | -2.35668 |
|  |  |  |  | COX11 | 2.428276 |  |  | COL4A1 | -3.21351 | WNT10A | -2.355 |
|  |  |  |  | GC | 2.4304 |  |  | VAT1L | -3.2105 | F12 | -2.35447 |
|  |  |  |  | PLAGL1 | 2.430472 |  |  | ZEB2 | -3.20848 | FUT1 | -2.35445 |
|  |  |  |  | DDX3X | 2.431113 |  |  | EPHB2 | -3.20028 | AASS | -2.35383 |
|  |  |  |  | DDOST | 2.432224 |  |  | SFMBT2 | -3.19584 | PRSS22 | -2.3529 |
|  |  |  |  | INSM1 | 2.43347 |  |  | SLFN11 | -3.19216 | ADAM19 | -2.35257 |
|  |  |  |  | DHRS2 | 2.434331 |  |  | CYR61 | -3.18463 | PTPRCAP | -2.35017 |
|  |  |  |  | UHRF1BP1L | 2.435139 |  |  | ITGAM | -3.17606 | LOC100505702 | -2.34931 |
|  |  |  |  | ABCC8 | 2.435811 |  |  | CLU | -3.17462 | HAL | -2.34445 |
|  |  |  |  | CHUK | 2.436506 |  |  | MS4A6A | -3.16828 | MKI67 | -2.34421 |
|  |  |  |  | ME2 | 2.44196 |  |  | MGP | -3.16105 | AEN | -2.34269 |
|  |  |  |  | IL13RA2 | 2.447926 |  |  | CCL5 | -3.16019 | FCN1 | -2.34199 |
|  |  |  |  | ORC3 | 2.452351 |  |  | PDPN | -3.15333 | CCR7 | -2.3417 |
|  |  |  |  | C3orf14 | 2.456561 |  |  | PLTP | -3.15025 | NDC80 | -2.33332 |
|  |  |  |  | CCDC47 | 2.457581 |  |  | CPLX1 | -3.14265 | PLA2G3 | -2.32862 |
|  |  |  |  | HADHA | 2.459226 |  |  | PELI1 | -3.13563 | SLC35E4 | -2.32741 |
|  |  |  |  | HIGD1A | 2.461718 |  |  | RCSD1 | -3.13266 | FAM3D | -2.32573 |
|  |  |  |  | UGP2 | 2.46571 |  |  | GZMA | -3.13033 | LYZ | -2.32339 |
|  |  |  |  | HAT1 | 2.468503 |  |  | NCKAP1L | -3.12639 | TNFRSF21 | -2.32236 |
|  |  |  |  | ST18 | 2.470036 |  |  | FCER1G | -3.12617 | PLBD1 | -2.31248 |
|  |  |  |  | PDCD10 | 2.472194 |  |  | PTX3 | -3.12244 | IFIT1 | -2.31202 |
|  |  |  |  | SEC11A | 2.479352 |  |  | FCN1 | -3.12196 | FOXRED2 | -2.30872 |
|  |  |  |  | WBP11 | 2.480229 |  |  | LCP2 | -3.11532 | CD2 | -2.30855 |
|  |  |  |  | EIF5 | 2.484033 |  |  | AGT | -3.11178 | CARD6 | -2.30811 |
|  |  |  |  | BEX4 | 2.485436 |  |  | GLT8D2 | -3.10021 | LDLR | -2.305 |
|  |  |  |  | EEF1E1 | 2.485448 |  |  | LGMN | -3.09687 | TLE3 | -2.30481 |
|  |  |  |  | RDX | 2.489345 |  |  | PDE4DIP | -3.09569 | CTPS1 | -2.3023 |
|  |  |  |  | SKIV2L2 | 2.490855 |  |  | GJA5 | -3.09566 | BUB1B | -2.30148 |
|  |  |  |  | PSMA3 | 2.495415 |  |  | PENK | -3.09296 | CNFN | -2.30074 |
|  |  |  |  | SLC35G2 | 2.495626 |  |  | PCSK5 | -3.08442 | KIF4A | -2.29858 |
|  |  |  |  | PRUNE2 | 2.508075 |  |  | PRSS23 | -3.08305 | CHST11 | -2.29644 |
|  |  |  |  | PTPRN2 | 2.508274 |  |  | COTL1 | -3.08068 | PPIF | -2.29623 |
|  |  |  |  | GALC | 2.511154 |  |  | KIAA1644 | -3.08041 | COL6A6 | -2.28978 |
|  |  |  |  | IPO5 | 2.518259 |  |  | DPT | -3.07814 | DCUN1D3 | -2.28774 |
|  |  |  |  | CDK7 | 2.519232 |  |  | MILR1 | -3.07798 | SGOL2 | -2.28601 |
|  |  |  |  | TUSC3 | 2.52023 |  |  | PTPRO | -3.07001 | ENAH | -2.28315 |
|  |  |  |  | USP16 | 2.521243 |  |  | SERPINB9 | -3.06717 | UBE2C | -2.27936 |
|  |  |  |  | VAMP7 | 2.523088 |  |  | SELPLG | -3.0663 | OVOL1 | -2.27499 |
|  |  |  |  | PGK1 | 2.527132 |  |  | ALOX5 | -3.06518 | TANGO6 | -2.27423 |
|  |  |  |  | PSAP | 2.529968 |  |  | HMCN1 | -3.06435 | SPCS3 | -2.26708 |
|  |  |  |  | ROBO1 | 2.530726 |  |  | OLFML2B | -3.06031 | NLRP2 | -2.26452 |
|  |  |  |  | PRDX3 | 2.530739 |  |  | AIM2 | -3.05342 | ATP11B | -2.25765 |
|  |  |  |  | PIKFYVE | 2.541202 |  |  | IGK | -3.04957 | DHRS9 | -2.2546 |
|  |  |  |  | SORL1 | 2.542904 |  |  | ADAM28 | -3.04779 | ASF1B | -2.25407 |
|  |  |  |  | BET1 | 2.546268 |  |  | TLR2 | -3.0468 | EXO1 | -2.25332 |
|  |  |  |  | CANX | 2.550224 |  |  | EVI2A | -3.04419 | DSCR10 | -2.25059 |
|  |  |  |  | SLC17A6 | 2.550631 |  |  | ACSL4 | -3.03191 | CDH3 | -2.24231 |
|  |  |  |  | CPNE3 | 2.55147 |  |  | CLEC12A | -3.0294 | CCNE2 | -2.24093 |
|  |  |  |  | SRP72 | 2.556489 |  |  | TNFSF15 | -3.00909 | CD47 | -2.23953 |
|  |  |  |  | PAPSS2 | 2.562638 |  |  | FN1 | -3.00081 | C12orf5 | -2.23394 |
|  |  |  |  | NUDT9 | 2.56871 |  |  | CYBA | -2.98619 | E2F8 | -2.23025 |
|  |  |  |  | TMEM135 | 2.572357 |  |  | CD86 | -2.98321 | SKA1 | -2.22971 |
|  |  |  |  | PSMD10 | 2.574243 |  |  | PTPRC | -2.97963 | CXCR6 | -2.22826 |
|  |  |  |  | CBFB | 2.577125 |  |  | SRPX2 | -2.97875 | HBEGF | -2.22448 |
|  |  |  |  | SLC25A32 | 2.578931 |  |  | FOLR2 | -2.97446 | MTFR2 | -2.22401 |
|  |  |  |  | GPX3 | 2.583322 |  |  | ARHGAP28 | -2.97315 | SLC38A5 | -2.22395 |
|  |  |  |  | DMXL1 | 2.585729 |  |  | C16orf54 | -2.96898 | PDSS1 | -2.22243 |
|  |  |  |  | METAP2 | 2.586734 |  |  | ACAN | -2.96881 | HK2 | -2.21395 |
|  |  |  |  | TRIM2 | 2.59992 |  |  | TREM2 | -2.96745 | SEL1L3 | -2.21348 |
|  |  |  |  | IMPA1 | 2.604124 |  |  | CP | -2.95694 | STAT1 | -2.20961 |
|  |  |  |  | UBE3B | 2.613657 |  |  | TYMP | -2.94903 | MPHOSPH6 | -2.20933 |
|  |  |  |  | AHCY | 2.617423 |  |  | CREB5 | -2.94752 | PLAT | -2.209 |
|  |  |  |  | COPB1 | 2.622132 |  |  | LHFPL2 | -2.94224 | ULBP2 | -2.20604 |
|  |  |  |  | ZNF207 | 2.626686 |  |  | DOK5 | -2.94154 | SERPINA3 | -2.19237 |
|  |  |  |  | DLD | 2.630547 |  |  | KLF6 | -2.93121 | SLC7A1 | -2.18927 |
|  |  |  |  | KIF5C | 2.631423 |  |  | TMEM176B | -2.93074 | TRG-AS1 | -2.18207 |
|  |  |  |  | PFKM | 2.634283 |  |  | LRP1 | -2.9277 | USP6NL-IT1 | -2.17535 |
|  |  |  |  | CLCN3 | 2.637293 |  |  | PCDH9 | -2.9269 | ARG1 | -2.17155 |
|  |  |  |  | TDP2 | 2.637659 |  |  | VCAN | -2.91847 | PARP14 | -2.15858 |
|  |  |  |  | RUFY3 | 2.637768 |  |  | SEMA3E | -2.91842 | APOL1 | -2.15527 |
|  |  |  |  | NEBL | 2.63819 |  |  | PLCE1 | -2.91307 | PRDM1 | -2.15355 |
|  |  |  |  | SLC25A12 | 2.642695 |  |  | KCNT2 | -2.90931 | IL12RB2 | -2.15292 |
|  |  |  |  | MRPL39 | 2.645932 |  |  | ADAMTSL4 | -2.90521 | TMC5 | -2.15186 |
|  |  |  |  | TMEM165 | 2.652017 |  |  | PIK3R5 | -2.90458 | KIAA1217 | -2.15048 |
|  |  |  |  | CXCL10 | 2.660003 |  |  | ARHGEF40 | -2.90185 | FGFBP1 | -2.14974 |
|  |  |  |  | AZIN1 | 2.660609 |  |  | MYLIP | -2.89798 | FCGR3B | -2.14022 |
|  |  |  |  | OSTM1 | 2.666183 |  |  | SEC24D | -2.89606 | SFT2D2 | -2.13728 |
|  |  |  |  | AMZ2 | 2.667117 |  |  | CD79A | -2.89066 | GGH | -2.13677 |
|  |  |  |  | SEC23B | 2.687126 |  |  | CD47 | -2.8898 | TOP2A | -2.13485 |
|  |  |  |  | ILF2 | 2.695353 |  |  | BANK1 | -2.8811 | ITGAL | -2.13466 |
|  |  |  |  | FAP | 2.697493 |  |  | PGF | -2.87922 | AFAP1L2 | -2.13137 |
|  |  |  |  | AKAP11 | 2.699181 |  |  | GAA | -2.87636 | CLCN5 | -2.12854 |
|  |  |  |  | TCEA1 | 2.704898 |  |  | JUN | -2.87331 | HMGB3P1 | -2.12552 |
|  |  |  |  | OAT | 2.707861 |  |  | FOXP1 | -2.87231 | CYP2E1 | -2.11364 |
|  |  |  |  | GYG1 | 2.709541 |  |  | PAPSS2 | -2.87203 | ADAP2 | -2.1111 |
|  |  |  |  | TOMM20 | 2.720349 |  |  | SCAF4 | -2.87135 | RGS18 | -2.10919 |
|  |  |  |  | RNF6 | 2.724109 |  |  | NAIP | -2.8605 | PRSS3 | -2.10498 |
|  |  |  |  | DCTN6 | 2.724142 |  |  | FAM20C | -2.85746 | TPX2 | -2.10497 |
|  |  |  |  | TMEM251 | 2.732779 |  |  | MGC24103 | -2.85328 | ATP10B | -2.1047 |
|  |  |  |  | TFRC | 2.733548 |  |  | SELP | -2.85281 | PTPN22 | -2.09654 |
|  |  |  |  | HMGCR | 2.733685 |  |  | MIR99AHG | -2.85047 | WFDC12 | -2.09555 |
|  |  |  |  | STMN2 | 2.740311 |  |  | GALNT10 | -2.84563 | MINA | -2.0933 |
|  |  |  |  | SERINC1 | 2.744212 |  |  | LRRFIP1 | -2.84461 | ACPP | -2.09184 |
|  |  |  |  | PRPS1 | 2.7597 |  |  | GPR34 | -2.84448 | PLAC8 | -2.09136 |
|  |  |  |  | SUCLG2 | 2.763107 |  |  | CD53 | -2.84132 | C9orf169 | -2.08831 |
|  |  |  |  | DNAJB9 | 2.763719 |  |  | TNFSF11 | -2.84043 | TPBG | -2.08686 |
|  |  |  |  | TMEM70 | 2.765856 |  |  | SLC39A14 | -2.83845 | C17orf96 | -2.08363 |
|  |  |  |  | TMOD1 | 2.783253 |  |  | MRC2 | -2.83039 | NAPSB | -2.08344 |
|  |  |  |  | TOR1AIP1 | 2.789396 |  |  | DOCK4 | -2.82662 | NDST1 | -2.0833 |
|  |  |  |  | ENTPD3 | 2.791457 |  |  | HTR2A | -2.82369 | TMEM184A | -2.08277 |
|  |  |  |  | PPP1R7 | 2.797549 |  |  | SIX2 | -2.81294 | POLE2 | -2.08046 |
|  |  |  |  | XRCC5 | 2.801911 |  |  | MICB | -2.81133 | FOXM1 | -2.07862 |
|  |  |  |  | CRYZ | 2.805062 |  |  | INHBA | -2.81011 | CENPE | -2.06887 |
|  |  |  |  | DHRS7 | 2.809782 |  |  | DZIP1 | -2.80894 | CCR1 | -2.06724 |
|  |  |  |  | LIPA | 2.82473 |  |  | ZNF595 | -2.806 | SLAMF8 | -2.06493 |
|  |  |  |  | SCG5 | 2.826827 |  |  | ALDH3B1 | -2.80459 | RDH10 | -2.06 |
|  |  |  |  | ABAT | 2.834732 |  |  | LYN | -2.80428 | RQCD1 | -2.05969 |
|  |  |  |  | PDIA6 | 2.845225 |  |  | TMEM106A | -2.79878 | TMEM86A | -2.05776 |
|  |  |  |  | ZNHIT3 | 2.84573 |  |  | GLUL | -2.79102 | FGFR1OP | -2.05411 |
|  |  |  |  | SGCE | 2.84582 |  |  | TIMP1 | -2.77992 | TMEM165 | -2.0535 |
|  |  |  |  | ATRX | 2.847925 |  |  | FAM46C | -2.77805 | CENPN | -2.04872 |
|  |  |  |  | SYBU | 2.87013 |  |  | CRLF1 | -2.77331 | APOBEC3B | -2.04431 |
|  |  |  |  | CHGB | 2.884222 |  |  | PTPRG | -2.77129 | C11orf82 | -2.03839 |
|  |  |  |  | ACSL1 | 2.88942 |  |  | FGF18 | -2.77068 | CLDN17 | -2.0358 |
|  |  |  |  | HSP90AB1 | 2.89049 |  |  | GPR1 | -2.76934 | BATF | -2.03552 |
|  |  |  |  | YBX1 | 2.896595 |  |  | NR4A1 | -2.76685 | S100P | -2.03316 |
|  |  |  |  | GNAS | 2.906148 |  |  | LOXL2 | -2.76632 | CCR5 | -2.03103 |
|  |  |  |  | UQCRC2 | 2.911679 |  |  | CBLB | -2.76545 | CCNE1 | -2.02916 |
|  |  |  |  | ISCA1 | 2.914845 |  |  | MZB1 | -2.76301 | SULT1E1 | -2.02773 |
|  |  |  |  | ITFG1 | 2.915031 |  |  | SEL1L3 | -2.75138 | TRANK1 | -2.0245 |
|  |  |  |  | LOC728392 | 2.945221 |  |  | B3GALNT1 | -2.74447 | MX2 | -2.02097 |
|  |  |  |  | SSX2IP | 2.953991 |  |  | HCK | -2.73877 | E2F7 | -2.0208 |
|  |  |  |  | ANKMY2 | 2.97449 |  |  | CMKLR1 | -2.72807 | TREM1 | -2.02044 |
|  |  |  |  | RRAGD | 2.990667 |  |  | CTGF | -2.72764 | IKBKE | -2.01712 |
|  |  |  |  | SH3GLB1 | 2.992735 |  |  | ENPP4 | -2.7269 | TLR2 | -2.01667 |
|  |  |  |  | EIF5A | 3.000454 |  |  | POU2F2 | -2.72578 | ESPL1 | -2.01058 |
|  |  |  |  | GOLGA5 | 3.005824 |  |  | RNF166 | -2.7223 | SLC16A6 | -2.00846 |
|  |  |  |  | ELOVL5 | 3.012465 |  |  | IRAK3 | -2.71021 | GZMK | -2.00503 |
|  |  |  |  | TMX4 | 3.014641 |  |  | ADAMTSL3 | -2.70803 | S100A2 | -2.00252 |
|  |  |  |  | ASPH | 3.016246 |  |  | GBP4 | -2.70786 | LGALS3BP | -2.00216 |
|  |  |  |  | SLC25A46 | 3.020981 |  |  | RSPO3 | -2.70116 | CMYA5 | 2.000976 |
|  |  |  |  | RBM3 | 3.034014 |  |  | DOCK8 | -2.69896 | GLI2 | 2.005647 |
|  |  |  |  | MFN1 | 3.062664 |  |  | KLF7 | -2.69674 | SUN1 | 2.01467 |
|  |  |  |  | SNAP25 | 3.070186 |  |  | PARVG | -2.69521 | NPTX2 | 2.014986 |
|  |  |  |  | QPCT | 3.071483 |  |  | CLEC7A | -2.69466 | TRIM52 | 2.019316 |
|  |  |  |  | EAPP | 3.09268 |  |  | SH2B3 | -2.6916 | NPTX1 | 2.025135 |
|  |  |  |  | TPD52 | 3.126249 |  |  | MEDAG | -2.68987 | AK7 | 2.026629 |
|  |  |  |  | CD99 | 3.148523 |  |  | MYO1F | -2.68835 | LINC00273 | 2.041378 |
|  |  |  |  | HOPX | 3.171656 |  |  | NKX3-2 | -2.68773 | TNNC1 | 2.057614 |
|  |  |  |  | CXCL11 | 3.178921 |  |  | DUSP6 | -2.68726 | LAMB4 | 2.063413 |
|  |  |  |  | SET | 3.197242 |  |  | COLEC12 | -2.68578 | AQP9 | 2.064208 |
|  |  |  |  | ACP1 | 3.204516 |  |  | VAV1 | -2.68575 | TNN | 2.066029 |
|  |  |  |  | SCP2 | 3.20497 |  |  | SLAMF7 | -2.68362 | GREM2 | 2.066329 |
|  |  |  |  | DHX15 | 3.22792 |  |  | IFI27 | -2.6833 | EGR1 | 2.071394 |
|  |  |  |  | EID1 | 3.241642 |  |  | TMTC1 | -2.68275 | SGCG | 2.071483 |
|  |  |  |  | PSMA1 | 3.28766 |  |  | LILRA2 | -2.68229 | DACT3 | 2.074464 |
|  |  |  |  | NAP1L2 | 3.306359 |  |  | SCARA3 | -2.68016 | LOC100507557 | 2.079651 |
|  |  |  |  | PFN2 | 3.319816 |  |  | PTPN2 | -2.6769 | APOC1 | 2.09937 |
|  |  |  |  | TRA2B | 3.330609 |  |  | NRP2 | -2.67548 | SERHL2 | 2.107301 |
|  |  |  |  | MAFB | 3.335885 |  |  | FAM26F | -2.67199 | BEX5 | 2.118566 |
|  |  |  |  | C1QBP | 3.355914 |  |  | SIX4 | -2.66764 | LONRF2 | 2.119292 |
|  |  |  |  | RAB1A | 3.363994 |  |  | ALDH1L2 | -2.6669 | GALNT15 | 2.139759 |
|  |  |  |  | LMBRD1 | 3.367766 |  |  | ARHGAP4 | -2.65965 | TIMP4 | 2.140207 |
|  |  |  |  | ALDH1A1 | 3.377195 |  |  | CCDC102B | -2.65331 | F3 | 2.14194 |
|  |  |  |  | ADCYAP1 | 3.394417 |  |  | LAT2 | -2.65048 | WNT7B | 2.15296 |
|  |  |  |  | SCG3 | 3.402168 |  |  | STC1 | -2.64856 | TMEM116 | 2.16532 |
|  |  |  |  | RCHY1 | 3.418259 |  |  | MERTK | -2.64372 | MYOM2 | 2.168947 |
|  |  |  |  | PEX2 | 3.433994 |  |  | ERRFI1 | -2.64089 | TMEM178A | 2.170461 |
|  |  |  |  | DLK1 | 3.444252 |  |  | NCF4 | -2.63984 | ZNF34 | 2.173411 |
|  |  |  |  | ARG2 | 3.484557 |  |  | CADM3 | -2.63677 | CLIP3 | 2.18077 |
|  |  |  |  | UPF3A | 3.484579 |  |  | RBMS3 | -2.63673 | CNTN4 | 2.189261 |
|  |  |  |  | RTN1 | 3.494004 |  |  | Sep-11 | -2.63606 | PKP2 | 2.193023 |
|  |  |  |  | SCG2 | 3.504214 |  |  | SLC15A3 | -2.63127 | PCDHGA8 | 2.195419 |
|  |  |  |  | SELT | 3.506728 |  |  | C4orf48 | -2.62841 | C14orf64 | 2.201084 |
|  |  |  |  | CLGN | 3.513426 |  |  | PTP4A3 | -2.61182 | ADSSL1 | 2.226453 |
|  |  |  |  | SCGN | 3.54645 |  |  | MDFIC | -2.6114 | HAO2 | 2.229044 |
|  |  |  |  | GREM1 | 3.613703 |  |  | TNFSF8 | -2.60914 | AXL | 2.229454 |
|  |  |  |  | PRKAR1A | 3.742411 |  |  | MUC8 | -2.60881 | ESPN | 2.232454 |
|  |  |  |  | RGS4 | 3.7429 |  |  | NLGN4X | -2.60619 | WNT2 | 2.251491 |
|  |  |  |  | RBP4 | 3.838784 |  |  | CTSB | -2.60131 | ZSCAN18 | 2.256672 |
|  |  |  |  | HADH | 3.853406 |  |  | KLF2 | -2.59617 | XKR4 | 2.284083 |
|  |  |  |  | UCHL1 | 3.879638 |  |  | SSC5D | -2.59534 | MIR4720 | 2.284626 |
|  |  |  |  | ERO1LB | 3.949182 |  |  | PROCR | -2.59484 | KRT79 | 2.294989 |
|  |  |  |  | IAPP | 3.975714 |  |  | CPNE5 | -2.59459 | LONRF1 | 2.29573 |
|  |  |  |  | PNMA2 | 4.047542 |  |  | FAM120A | -2.59084 | MYOCD | 2.297451 |
|  |  |  |  | CPE | 4.603779 |  |  | TAGAP | -2.59002 | LOC285084 | 2.300686 |
|  |  |  |  | PCSK1 | 4.70215 |  |  | NRXN2 | -2.58636 | GLIS1 | 2.301213 |
|  |  |  |  | ENPP2 | 4.785815 |  |  | SOX17 | -2.58508 | CYP2J2 | 2.313067 |
|  |  |  |  |  |  |  |  | PIK3AP1 | -2.5835 | GATA6 | 2.316813 |
|  |  |  |  |  |  |  |  | GLIPR1 | -2.58348 | TPPP | 2.318024 |
|  |  |  |  |  |  |  |  | VEGFC | -2.57771 | FA2H | 2.325322 |
|  |  |  |  |  |  |  |  | SGCD | -2.57346 | HSPB7 | 2.332604 |
|  |  |  |  |  |  |  |  | PTGER2 | -2.57171 | LOC157562 | 2.336713 |
|  |  |  |  |  |  |  |  | SPON1 | -2.57073 | LOC338799 | 2.345315 |
|  |  |  |  |  |  |  |  | LYZ | -2.5678 | TUBGCP6 | 2.346851 |
|  |  |  |  |  |  |  |  | SCARF1 | -2.56752 | GLDC | 2.364756 |
|  |  |  |  |  |  |  |  | DAB2 | -2.56618 | LOC100506098 | 2.383484 |
|  |  |  |  |  |  |  |  | OSMR | -2.5643 | TNMD | 2.384479 |
|  |  |  |  |  |  |  |  | SLAMF8 | -2.56123 | FAM189A2 | 2.390524 |
|  |  |  |  |  |  |  |  | TFPI | -2.56004 | CORO2B | 2.453654 |
|  |  |  |  |  |  |  |  | ALPL | -2.55869 | LINC00312 | 2.459245 |
|  |  |  |  |  |  |  |  | IL10RA | -2.55522 | MMP28 | 2.474884 |
|  |  |  |  |  |  |  |  | CCPG1 | -2.55373 | ANKFN1 | 2.480062 |
|  |  |  |  |  |  |  |  | CYTH4 | -2.55229 | SCGB2A1 | 2.504521 |
|  |  |  |  |  |  |  |  | HTR2B | -2.54705 | AGR3 | 2.535073 |
|  |  |  |  |  |  |  |  | CH25H | -2.54427 | ENPP5 | 2.563558 |
|  |  |  |  |  |  |  |  | ADAMTS2 | -2.54107 | C9orf152 | 2.566308 |
|  |  |  |  |  |  |  |  | PLEKHG2 | -2.54009 | PHYHIP | 2.575854 |
|  |  |  |  |  |  |  |  | RAB29 | -2.5398 | WFDC3 | 2.583236 |
|  |  |  |  |  |  |  |  | PRRX1 | -2.53825 | PIK3C2G | 2.586034 |
|  |  |  |  |  |  |  |  | STAT1 | -2.53569 | ARHGEF26 | 2.61234 |
|  |  |  |  |  |  |  |  | GALNT15 | -2.53404 | TNNT1 | 2.620061 |
|  |  |  |  |  |  |  |  | LACTB | -2.534 | CHAD | 2.661137 |
|  |  |  |  |  |  |  |  | RARRES3 | -2.52978 | SLC14A1 | 2.662139 |
|  |  |  |  |  |  |  |  | EDEM3 | -2.52731 | SERPINA12 | 2.680623 |
|  |  |  |  |  |  |  |  | FLJ32255 | -2.52667 | KIAA1244 | 2.687861 |
|  |  |  |  |  |  |  |  | HCLS1 | -2.52657 | SLC1A6 | 2.706334 |
|  |  |  |  |  |  |  |  | LOXL1 | -2.52605 | LOC100130476 | 2.717055 |
|  |  |  |  |  |  |  |  | PTHLH | -2.52245 | GSTA3 | 2.726481 |
|  |  |  |  |  |  |  |  | TNFSF13B | -2.52157 | AWAT1 | 2.734952 |
|  |  |  |  |  |  |  |  | FGR | -2.51941 | SCIN | 2.754802 |
|  |  |  |  |  |  |  |  | APOL6 | -2.51778 | PLD6 | 2.767332 |
|  |  |  |  |  |  |  |  | ISG15 | -2.51735 | THRSP | 2.778149 |
|  |  |  |  |  |  |  |  | GGA1 | -2.51623 | FAM221A | 2.780771 |
|  |  |  |  |  |  |  |  | PIK3CG | -2.51356 | HSD11B1 | 2.808884 |
|  |  |  |  |  |  |  |  | FERMT3 | -2.51085 | TMEM56 | 2.879259 |
|  |  |  |  |  |  |  |  | GRB10 | -2.50998 | LOC643792 | 2.903111 |
|  |  |  |  |  |  |  |  | KCNE3 | -2.50917 | ZDHHC11 | 2.908944 |
|  |  |  |  |  |  |  |  | LINC-PINT | -2.50736 | ACADL | 2.940452 |
|  |  |  |  |  |  |  |  | RAPGEF2 | -2.50682 | LOC728730 | 2.957802 |
|  |  |  |  |  |  |  |  | MS4A14 | -2.5067 | PTPN21 | 2.962926 |
|  |  |  |  |  |  |  |  | PRKAA1 | -2.50636 | ZBTB16 | 2.966531 |
|  |  |  |  |  |  |  |  | HNRNPM | -2.50363 | GAN | 3.033519 |
|  |  |  |  |  |  |  |  | CHSY3 | -2.50136 | ACSBG1 | 3.06407 |
|  |  |  |  |  |  |  |  | EAF2 | -2.49855 | ANKRD33B | 3.068611 |
|  |  |  |  |  |  |  |  | CSF1R | -2.49722 | SP8 | 3.114578 |
|  |  |  |  |  |  |  |  | HRH1 | -2.4941 | SLC46A2 | 3.127188 |
|  |  |  |  |  |  |  |  | THY1 | -2.49287 | FABP7 | 3.138685 |
|  |  |  |  |  |  |  |  | GBP5 | -2.49025 | C5orf46 | 3.185594 |
|  |  |  |  |  |  |  |  | TMEM163 | -2.48859 | HSD3B1 | 3.195125 |
|  |  |  |  |  |  |  |  | MCL1 | -2.48518 | CHP2 | 3.315678 |
|  |  |  |  |  |  |  |  | MMP13 | -2.48501 | MYOC | 3.354548 |
|  |  |  |  |  |  |  |  | LAPTM5 | -2.48444 | KRT77 | 3.367228 |
|  |  |  |  |  |  |  |  | TNFRSF1B | -2.48371 | TMEM255A | 3.387341 |
|  |  |  |  |  |  |  |  | PRO2852 | -2.48324 | ELOVL3 | 3.479036 |
|  |  |  |  |  |  |  |  | IGLL3P | -2.48274 | FADS2 | 3.603767 |
|  |  |  |  |  |  |  |  | CXCL16 | -2.4822 | WIF1 | 3.744521 |
|  |  |  |  |  |  |  |  | GZMB | -2.48211 | HS3ST6 | 3.764552 |
|  |  |  |  |  |  |  |  | EMILIN1 | -2.48206 | CCL2 | 4.041537 |
|  |  |  |  |  |  |  |  | S100A9 | -2.48167 | PM20D1 | 4.056329 |
|  |  |  |  |  |  |  |  | LILRA6 | -2.48131 | IL37 | 4.16303 |
|  |  |  |  |  |  |  |  | CLEC4A | -2.48041 | BTC | 5.071634 |
|  |  |  |  |  |  |  |  | TBC1D8B | -2.47726 |  |  |
|  |  |  |  |  |  |  |  | ADAP2 | -2.47343 |  |  |
|  |  |  |  |  |  |  |  | ARL11 | -2.47341 |  |  |
|  |  |  |  |  |  |  |  | PLSCR1 | -2.47048 |  |  |
|  |  |  |  |  |  |  |  | PCOLCE2 | -2.46965 |  |  |
|  |  |  |  |  |  |  |  | KERA | -2.4694 |  |  |
|  |  |  |  |  |  |  |  | EFEMP1 | -2.46639 |  |  |
|  |  |  |  |  |  |  |  | CHST11 | -2.4633 |  |  |
|  |  |  |  |  |  |  |  | ADAMTS6 | -2.46244 |  |  |
|  |  |  |  |  |  |  |  | HSPA6 | -2.46153 |  |  |
|  |  |  |  |  |  |  |  | TTYH3 | -2.46136 |  |  |
|  |  |  |  |  |  |  |  | GZMK | -2.45656 |  |  |
|  |  |  |  |  |  |  |  | NUPL1 | -2.45543 |  |  |
|  |  |  |  |  |  |  |  | JMJD1C | -2.4544 |  |  |
|  |  |  |  |  |  |  |  | CSGALNACT2 | -2.45402 |  |  |
|  |  |  |  |  |  |  |  | PCDH17 | -2.45031 |  |  |
|  |  |  |  |  |  |  |  | C1S | -2.44755 |  |  |
|  |  |  |  |  |  |  |  | EVI2B | -2.44704 |  |  |
|  |  |  |  |  |  |  |  | ISLR | -2.44219 |  |  |
|  |  |  |  |  |  |  |  | PXDN | -2.44078 |  |  |
|  |  |  |  |  |  |  |  | CALML4 | -2.43861 |  |  |
|  |  |  |  |  |  |  |  | SAMHD1 | -2.43745 |  |  |
|  |  |  |  |  |  |  |  | ENTPD1 | -2.43704 |  |  |
|  |  |  |  |  |  |  |  | HLA-F | -2.43536 |  |  |
|  |  |  |  |  |  |  |  | DRAM1 | -2.4329 |  |  |
|  |  |  |  |  |  |  |  | Sep-06 | -2.43172 |  |  |
|  |  |  |  |  |  |  |  | SIGLEC7 | -2.43017 |  |  |
|  |  |  |  |  |  |  |  | TLR1 | -2.42911 |  |  |
|  |  |  |  |  |  |  |  | MTUS2 | -2.42776 |  |  |
|  |  |  |  |  |  |  |  | TMEM176A | -2.4268 |  |  |
|  |  |  |  |  |  |  |  | ZFP36 | -2.42209 |  |  |
|  |  |  |  |  |  |  |  | MAGI2-AS3 | -2.41926 |  |  |
|  |  |  |  |  |  |  |  | ANPEP | -2.41848 |  |  |
|  |  |  |  |  |  |  |  | GEM | -2.41807 |  |  |
|  |  |  |  |  |  |  |  | TAF1A | -2.41719 |  |  |
|  |  |  |  |  |  |  |  | SLC4A7 | -2.41578 |  |  |
|  |  |  |  |  |  |  |  | SAMD9 | -2.41366 |  |  |
|  |  |  |  |  |  |  |  | GABRB2 | -2.41322 |  |  |
|  |  |  |  |  |  |  |  | PLEKHA4 | -2.40981 |  |  |
|  |  |  |  |  |  |  |  | DOCK2 | -2.40555 |  |  |
|  |  |  |  |  |  |  |  | IGFBP4 | -2.4034 |  |  |
|  |  |  |  |  |  |  |  | GADD45B | -2.40013 |  |  |
|  |  |  |  |  |  |  |  | ODF2L | -2.39607 |  |  |
|  |  |  |  |  |  |  |  | SNRNP200 | -2.39585 |  |  |
|  |  |  |  |  |  |  |  | COL4A2 | -2.39492 |  |  |
|  |  |  |  |  |  |  |  | JAK3 | -2.39076 |  |  |
|  |  |  |  |  |  |  |  | ITGBL1 | -2.39061 |  |  |
|  |  |  |  |  |  |  |  | CALR | -2.38944 |  |  |
|  |  |  |  |  |  |  |  | MGAT4A | -2.38854 |  |  |
|  |  |  |  |  |  |  |  | RGCC | -2.3865 |  |  |
|  |  |  |  |  |  |  |  | CYTIP | -2.38519 |  |  |
|  |  |  |  |  |  |  |  | PRSS35 | -2.38436 |  |  |
|  |  |  |  |  |  |  |  | ANKH | -2.38148 |  |  |
|  |  |  |  |  |  |  |  | PDE7B | -2.37486 |  |  |
|  |  |  |  |  |  |  |  | FABP3 | -2.37257 |  |  |
|  |  |  |  |  |  |  |  | KDELR3 | -2.36866 |  |  |
|  |  |  |  |  |  |  |  | PITPNC1 | -2.3683 |  |  |
|  |  |  |  |  |  |  |  | TMEM100 | -2.3681 |  |  |
|  |  |  |  |  |  |  |  | CD38 | -2.36693 |  |  |
|  |  |  |  |  |  |  |  | ST3GAL3 | -2.36343 |  |  |
|  |  |  |  |  |  |  |  | TMEM173 | -2.36315 |  |  |
|  |  |  |  |  |  |  |  | UBE2D1 | -2.36291 |  |  |
|  |  |  |  |  |  |  |  | ORAI2 | -2.35636 |  |  |
|  |  |  |  |  |  |  |  | COL3A1 | -2.3544 |  |  |
|  |  |  |  |  |  |  |  | FCRL5 | -2.35251 |  |  |
|  |  |  |  |  |  |  |  | SLCO2A1 | -2.3505 |  |  |
|  |  |  |  |  |  |  |  | ICAM1 | -2.34842 |  |  |
|  |  |  |  |  |  |  |  | FLJ31306 | -2.34779 |  |  |
|  |  |  |  |  |  |  |  | TPK1 | -2.34655 |  |  |
|  |  |  |  |  |  |  |  | LGALS9 | -2.34142 |  |  |
|  |  |  |  |  |  |  |  | MUC4 | -2.34084 |  |  |
|  |  |  |  |  |  |  |  | STEAP2 | -2.33748 |  |  |
|  |  |  |  |  |  |  |  | ITPRIP | -2.33707 |  |  |
|  |  |  |  |  |  |  |  | RNASE2 | -2.33607 |  |  |
|  |  |  |  |  |  |  |  | PYHIN1 | -2.33566 |  |  |
|  |  |  |  |  |  |  |  | NRGN | -2.33234 |  |  |
|  |  |  |  |  |  |  |  | MAP3K8 | -2.33104 |  |  |
|  |  |  |  |  |  |  |  | CALHM2 | -2.33054 |  |  |
|  |  |  |  |  |  |  |  | ITGA4 | -2.32964 |  |  |
|  |  |  |  |  |  |  |  | BMP1 | -2.32865 |  |  |
|  |  |  |  |  |  |  |  | PCOLCE | -2.32837 |  |  |
|  |  |  |  |  |  |  |  | CCL2 | -2.32775 |  |  |
|  |  |  |  |  |  |  |  | HLA-DRB4 | -2.32506 |  |  |
|  |  |  |  |  |  |  |  | RUNX1-IT1 | -2.3245 |  |  |
|  |  |  |  |  |  |  |  | GPC6 | -2.32404 |  |  |
|  |  |  |  |  |  |  |  | SAP30 | -2.32311 |  |  |
|  |  |  |  |  |  |  |  | RAB20 | -2.3224 |  |  |
|  |  |  |  |  |  |  |  | PPAPDC1B | -2.31691 |  |  |
|  |  |  |  |  |  |  |  | IL4I1 | -2.31569 |  |  |
|  |  |  |  |  |  |  |  | AGPAT4 | -2.30948 |  |  |
|  |  |  |  |  |  |  |  | COL5A2 | -2.30647 |  |  |
|  |  |  |  |  |  |  |  | LUM | -2.30511 |  |  |
|  |  |  |  |  |  |  |  | HTRA3 | -2.30342 |  |  |
|  |  |  |  |  |  |  |  | LOC100505812 | -2.29907 |  |  |
|  |  |  |  |  |  |  |  | SLC20A1 | -2.29899 |  |  |
|  |  |  |  |  |  |  |  | CHST2 | -2.29897 |  |  |
|  |  |  |  |  |  |  |  | ABL2 | -2.29873 |  |  |
|  |  |  |  |  |  |  |  | C1GALT1 | -2.29841 |  |  |
|  |  |  |  |  |  |  |  | COL11A1 | -2.29717 |  |  |
|  |  |  |  |  |  |  |  | ARHGAP30 | -2.297 |  |  |
|  |  |  |  |  |  |  |  | ADCY7 | -2.29384 |  |  |
|  |  |  |  |  |  |  |  | ACP5 | -2.29361 |  |  |
|  |  |  |  |  |  |  |  | LY86 | -2.29006 |  |  |
|  |  |  |  |  |  |  |  | JUNB | -2.28841 |  |  |
|  |  |  |  |  |  |  |  | CHD9 | -2.28571 |  |  |
|  |  |  |  |  |  |  |  | CD72 | -2.2856 |  |  |
|  |  |  |  |  |  |  |  | THBS3 | -2.28466 |  |  |
|  |  |  |  |  |  |  |  | SPI1 | -2.28454 |  |  |
|  |  |  |  |  |  |  |  | ARHGAP9 | -2.28362 |  |  |
|  |  |  |  |  |  |  |  | DUSP1 | -2.28276 |  |  |
|  |  |  |  |  |  |  |  | CCDC69 | -2.28167 |  |  |
|  |  |  |  |  |  |  |  | TNFRSF10B | -2.28157 |  |  |
|  |  |  |  |  |  |  |  | NABP1 | -2.28099 |  |  |
|  |  |  |  |  |  |  |  | IDUA | -2.28008 |  |  |
|  |  |  |  |  |  |  |  | SPOCK1 | -2.27883 |  |  |
|  |  |  |  |  |  |  |  | ODF3B | -2.27549 |  |  |
|  |  |  |  |  |  |  |  | FAM198B | -2.27429 |  |  |
|  |  |  |  |  |  |  |  | MXRA8 | -2.27386 |  |  |
|  |  |  |  |  |  |  |  | GUSBP11 | -2.27065 |  |  |
|  |  |  |  |  |  |  |  | SASH3 | -2.27023 |  |  |
|  |  |  |  |  |  |  |  | ALOX5AP | -2.26869 |  |  |
|  |  |  |  |  |  |  |  | APCDD1L | -2.26116 |  |  |
|  |  |  |  |  |  |  |  | DPYSL3 | -2.2594 |  |  |
|  |  |  |  |  |  |  |  | IKBIP | -2.25594 |  |  |
|  |  |  |  |  |  |  |  | PCNX | -2.25513 |  |  |
|  |  |  |  |  |  |  |  | DNALI1 | -2.25511 |  |  |
|  |  |  |  |  |  |  |  | PTAFR | -2.25316 |  |  |
|  |  |  |  |  |  |  |  | KIAA1755 | -2.25045 |  |  |
|  |  |  |  |  |  |  |  | PREX1 | -2.24993 |  |  |
|  |  |  |  |  |  |  |  | MRC1 | -2.24781 |  |  |
|  |  |  |  |  |  |  |  | ITGA10 | -2.24759 |  |  |
|  |  |  |  |  |  |  |  | COL5A1 | -2.24707 |  |  |
|  |  |  |  |  |  |  |  | LRRC25 | -2.24153 |  |  |
|  |  |  |  |  |  |  |  | TCN2 | -2.24139 |  |  |
|  |  |  |  |  |  |  |  | TGFB3 | -2.24136 |  |  |
|  |  |  |  |  |  |  |  | LPXN | -2.24095 |  |  |
|  |  |  |  |  |  |  |  | HOXB6 | -2.23836 |  |  |
|  |  |  |  |  |  |  |  | MLKL | -2.23545 |  |  |
|  |  |  |  |  |  |  |  | CCR5 | -2.23501 |  |  |
|  |  |  |  |  |  |  |  | DNAJB9 | -2.23368 |  |  |
|  |  |  |  |  |  |  |  | C1orf54 | -2.23151 |  |  |
|  |  |  |  |  |  |  |  | ITGB2-AS1 | -2.2309 |  |  |
|  |  |  |  |  |  |  |  | LRRN3 | -2.22967 |  |  |
|  |  |  |  |  |  |  |  | FMNL1 | -2.22892 |  |  |
|  |  |  |  |  |  |  |  | SKIL | -2.22781 |  |  |
|  |  |  |  |  |  |  |  | CELF2 | -2.22726 |  |  |
|  |  |  |  |  |  |  |  | PHLDA1 | -2.22624 |  |  |
|  |  |  |  |  |  |  |  | MME | -2.22573 |  |  |
|  |  |  |  |  |  |  |  | MED13L | -2.22346 |  |  |
|  |  |  |  |  |  |  |  | RPS27 | -2.22154 |  |  |
|  |  |  |  |  |  |  |  | DENND5B | -2.2211 |  |  |
|  |  |  |  |  |  |  |  | CD180 | -2.21529 |  |  |
|  |  |  |  |  |  |  |  | FMOD | -2.21391 |  |  |
|  |  |  |  |  |  |  |  | HAPLN1 | -2.21235 |  |  |
|  |  |  |  |  |  |  |  | ZCCHC11 | -2.21205 |  |  |
|  |  |  |  |  |  |  |  | FZD2 | -2.21065 |  |  |
|  |  |  |  |  |  |  |  | CSGALNACT1 | -2.21057 |  |  |
|  |  |  |  |  |  |  |  | SMIM3 | -2.21018 |  |  |
|  |  |  |  |  |  |  |  | NPL | -2.20894 |  |  |
|  |  |  |  |  |  |  |  | IGFBP7 | -2.20671 |  |  |
|  |  |  |  |  |  |  |  | ADAMTS5 | -2.2058 |  |  |
|  |  |  |  |  |  |  |  | COLGALT2 | -2.20504 |  |  |
|  |  |  |  |  |  |  |  | MAP1S | -2.20269 |  |  |
|  |  |  |  |  |  |  |  | ARHGAP33 | -2.20184 |  |  |
|  |  |  |  |  |  |  |  | ICAM2 | -2.20051 |  |  |
|  |  |  |  |  |  |  |  | GPRC5A | -2.19877 |  |  |
|  |  |  |  |  |  |  |  | ARSJ | -2.1977 |  |  |
|  |  |  |  |  |  |  |  | ZFHX3 | -2.19687 |  |  |
|  |  |  |  |  |  |  |  | LRRC4C | -2.19675 |  |  |
|  |  |  |  |  |  |  |  | APOBEC3G | -2.19522 |  |  |
|  |  |  |  |  |  |  |  | CCR7 | -2.19451 |  |  |
|  |  |  |  |  |  |  |  | EHBP1L1 | -2.19131 |  |  |
|  |  |  |  |  |  |  |  | C2orf40 | -2.19123 |  |  |
|  |  |  |  |  |  |  |  | HMHA1 | -2.19022 |  |  |
|  |  |  |  |  |  |  |  | PXDNL | -2.18817 |  |  |
|  |  |  |  |  |  |  |  | MGAT4C | -2.18774 |  |  |
|  |  |  |  |  |  |  |  | CARD16 | -2.18751 |  |  |
|  |  |  |  |  |  |  |  | MFNG | -2.18746 |  |  |
|  |  |  |  |  |  |  |  | SH3TC1 | -2.18538 |  |  |
|  |  |  |  |  |  |  |  | GIMAP7 | -2.18497 |  |  |
|  |  |  |  |  |  |  |  | CSPG4 | -2.18251 |  |  |
|  |  |  |  |  |  |  |  | DERL3 | -2.18166 |  |  |
|  |  |  |  |  |  |  |  | COPA | -2.18066 |  |  |
|  |  |  |  |  |  |  |  | EMP3 | -2.17862 |  |  |
|  |  |  |  |  |  |  |  | LTBP2 | -2.17663 |  |  |
|  |  |  |  |  |  |  |  | UAP1 | -2.17637 |  |  |
|  |  |  |  |  |  |  |  | METAP2 | -2.17518 |  |  |
|  |  |  |  |  |  |  |  | SDK1 | -2.1732 |  |  |
|  |  |  |  |  |  |  |  | KCND3 | -2.17032 |  |  |
|  |  |  |  |  |  |  |  | XAF1 | -2.16637 |  |  |
|  |  |  |  |  |  |  |  | AIF1 | -2.16502 |  |  |
|  |  |  |  |  |  |  |  | C10orf10 | -2.16414 |  |  |
|  |  |  |  |  |  |  |  | IKZF1 | -2.1626 |  |  |
|  |  |  |  |  |  |  |  | CD4 | -2.16219 |  |  |
|  |  |  |  |  |  |  |  | CD37 | -2.16199 |  |  |
|  |  |  |  |  |  |  |  | CARD6 | -2.1605 |  |  |
|  |  |  |  |  |  |  |  | FAIM3 | -2.15979 |  |  |
|  |  |  |  |  |  |  |  | DOK1 | -2.15964 |  |  |
|  |  |  |  |  |  |  |  | SOX18 | -2.15933 |  |  |
|  |  |  |  |  |  |  |  | SLC43A2 | -2.15895 |  |  |
|  |  |  |  |  |  |  |  | MAN1A1 | -2.15806 |  |  |
|  |  |  |  |  |  |  |  | STK17B | -2.15703 |  |  |
|  |  |  |  |  |  |  |  | RNASE1 | -2.15582 |  |  |
|  |  |  |  |  |  |  |  | MPP1 | -2.15161 |  |  |
|  |  |  |  |  |  |  |  | MEG3 | -2.15117 |  |  |
|  |  |  |  |  |  |  |  | RCN3 | -2.15079 |  |  |
|  |  |  |  |  |  |  |  | CPNE8 | -2.14887 |  |  |
|  |  |  |  |  |  |  |  | CD300A | -2.14665 |  |  |
|  |  |  |  |  |  |  |  | PDE3A | -2.1452 |  |  |
|  |  |  |  |  |  |  |  | ARPC1B | -2.14507 |  |  |
|  |  |  |  |  |  |  |  | CHI3L1 | -2.14115 |  |  |
|  |  |  |  |  |  |  |  | ANKRD10-IT1 | -2.14101 |  |  |
|  |  |  |  |  |  |  |  | HLA-DOA | -2.14026 |  |  |
|  |  |  |  |  |  |  |  | SYNDIG1 | -2.14009 |  |  |
|  |  |  |  |  |  |  |  | DPYD | -2.13858 |  |  |
|  |  |  |  |  |  |  |  | MAP9 | -2.13674 |  |  |
|  |  |  |  |  |  |  |  | NR4A3 | -2.13503 |  |  |
|  |  |  |  |  |  |  |  | GPR65 | -2.13447 |  |  |
|  |  |  |  |  |  |  |  | TMEM119 | -2.13394 |  |  |
|  |  |  |  |  |  |  |  | ZBTB10 | -2.13356 |  |  |
|  |  |  |  |  |  |  |  | GPCPD1 | -2.12662 |  |  |
|  |  |  |  |  |  |  |  | LACC1 | -2.12443 |  |  |
|  |  |  |  |  |  |  |  | BAX | -2.1241 |  |  |
|  |  |  |  |  |  |  |  | MYO1G | -2.12295 |  |  |
|  |  |  |  |  |  |  |  | PDLIM3 | -2.12271 |  |  |
|  |  |  |  |  |  |  |  | MIDN | -2.12243 |  |  |
|  |  |  |  |  |  |  |  | LRRC15 | -2.11939 |  |  |
|  |  |  |  |  |  |  |  | P2RY6 | -2.11375 |  |  |
|  |  |  |  |  |  |  |  | ADCY1 | -2.11103 |  |  |
|  |  |  |  |  |  |  |  | MAP7D3 | -2.11012 |  |  |
|  |  |  |  |  |  |  |  | PIWIL4 | -2.10816 |  |  |
|  |  |  |  |  |  |  |  | CLECL1 | -2.10758 |  |  |
|  |  |  |  |  |  |  |  | TDO2 | -2.10728 |  |  |
|  |  |  |  |  |  |  |  | RAB31 | -2.10703 |  |  |
|  |  |  |  |  |  |  |  | ITGAL | -2.10676 |  |  |
|  |  |  |  |  |  |  |  | PRKD2 | -2.10475 |  |  |
|  |  |  |  |  |  |  |  | SLC25A37 | -2.10246 |  |  |
|  |  |  |  |  |  |  |  | ANGPTL1 | -2.10203 |  |  |
|  |  |  |  |  |  |  |  | TACC1 | -2.10173 |  |  |
|  |  |  |  |  |  |  |  | NTN4 | -2.10083 |  |  |
|  |  |  |  |  |  |  |  | CDK14 | -2.10052 |  |  |
|  |  |  |  |  |  |  |  | KDM6B | -2.09972 |  |  |
|  |  |  |  |  |  |  |  | PLEKHO2 | -2.09881 |  |  |
|  |  |  |  |  |  |  |  | KCNJ6 | -2.09804 |  |  |
|  |  |  |  |  |  |  |  | RERG | -2.09686 |  |  |
|  |  |  |  |  |  |  |  | HLA-DMA | -2.09445 |  |  |
|  |  |  |  |  |  |  |  | ZFYVE16 | -2.09069 |  |  |
|  |  |  |  |  |  |  |  | SLC38A7 | -2.09038 |  |  |
|  |  |  |  |  |  |  |  | ARHGAP20 | -2.09024 |  |  |
|  |  |  |  |  |  |  |  | LINC01296 | -2.08884 |  |  |
|  |  |  |  |  |  |  |  | NBPF10 | -2.08761 |  |  |
|  |  |  |  |  |  |  |  | ENG | -2.08752 |  |  |
|  |  |  |  |  |  |  |  | ZNF385D | -2.08642 |  |  |
|  |  |  |  |  |  |  |  | MAB21L1 | -2.0862 |  |  |
|  |  |  |  |  |  |  |  | PER1 | -2.08607 |  |  |
|  |  |  |  |  |  |  |  | GBP1 | -2.0856 |  |  |
|  |  |  |  |  |  |  |  | CXCL11 | -2.08533 |  |  |
|  |  |  |  |  |  |  |  | SLN | -2.08505 |  |  |
|  |  |  |  |  |  |  |  | EMILIN2 | -2.08342 |  |  |
|  |  |  |  |  |  |  |  | KDELC1 | -2.08275 |  |  |
|  |  |  |  |  |  |  |  | MUC5AC | -2.08218 |  |  |
|  |  |  |  |  |  |  |  | IL27RA | -2.0796 |  |  |
|  |  |  |  |  |  |  |  | FUT4 | -2.07701 |  |  |
|  |  |  |  |  |  |  |  | GABRA4 | -2.0766 |  |  |
|  |  |  |  |  |  |  |  | DPY19L1 | -2.07462 |  |  |
|  |  |  |  |  |  |  |  | CSF2RB | -2.07316 |  |  |
|  |  |  |  |  |  |  |  | SP140L | -2.06907 |  |  |
|  |  |  |  |  |  |  |  | LOC100288570 | -2.06862 |  |  |
|  |  |  |  |  |  |  |  | MALAT1 | -2.06702 |  |  |
|  |  |  |  |  |  |  |  | TENM1 | -2.06696 |  |  |
|  |  |  |  |  |  |  |  | GALNT2 | -2.06465 |  |  |
|  |  |  |  |  |  |  |  | PARP14 | -2.06463 |  |  |
|  |  |  |  |  |  |  |  | EPB41L2 | -2.06424 |  |  |
|  |  |  |  |  |  |  |  | JAK2 | -2.06335 |  |  |
|  |  |  |  |  |  |  |  | LCA5 | -2.0628 |  |  |
|  |  |  |  |  |  |  |  | IFNAR2 | -2.06179 |  |  |
|  |  |  |  |  |  |  |  | PSD3 | -2.06095 |  |  |
|  |  |  |  |  |  |  |  | F5 | -2.06076 |  |  |
|  |  |  |  |  |  |  |  | GUCY1B3 | -2.05958 |  |  |
|  |  |  |  |  |  |  |  | TCIRG1 | -2.05826 |  |  |
|  |  |  |  |  |  |  |  | SHC1 | -2.05714 |  |  |
|  |  |  |  |  |  |  |  | PDE4B | -2.05651 |  |  |
|  |  |  |  |  |  |  |  | CDH11 | -2.05527 |  |  |
|  |  |  |  |  |  |  |  | EFCAB2 | -2.05397 |  |  |
|  |  |  |  |  |  |  |  | PHF21A | -2.05266 |  |  |
|  |  |  |  |  |  |  |  | CPXM2 | -2.05262 |  |  |
|  |  |  |  |  |  |  |  | TSHZ3 | -2.04712 |  |  |
|  |  |  |  |  |  |  |  | IFFO1 | -2.04647 |  |  |
|  |  |  |  |  |  |  |  | IL15 | -2.04516 |  |  |
|  |  |  |  |  |  |  |  | LTBP3 | -2.044 |  |  |
|  |  |  |  |  |  |  |  | GRAMD1A | -2.04212 |  |  |
|  |  |  |  |  |  |  |  | NEURL2 | -2.03821 |  |  |
|  |  |  |  |  |  |  |  | MARK3 | -2.03815 |  |  |
|  |  |  |  |  |  |  |  | TRAM2 | -2.03745 |  |  |
|  |  |  |  |  |  |  |  | P2RY12 | -2.03594 |  |  |
|  |  |  |  |  |  |  |  | ZNF236 | -2.03563 |  |  |
|  |  |  |  |  |  |  |  | NKG7 | -2.03422 |  |  |
|  |  |  |  |  |  |  |  | GIMAP2 | -2.03366 |  |  |
|  |  |  |  |  |  |  |  | RFTN2 | -2.03347 |  |  |
|  |  |  |  |  |  |  |  | ELMOD2 | -2.03043 |  |  |
|  |  |  |  |  |  |  |  | TNFAIP2 | -2.03019 |  |  |
|  |  |  |  |  |  |  |  | HTRA1 | -2.02973 |  |  |
|  |  |  |  |  |  |  |  | GBGT1 | -2.02895 |  |  |
|  |  |  |  |  |  |  |  | PCED1A | -2.02868 |  |  |
|  |  |  |  |  |  |  |  | RRBP1 | -2.02859 |  |  |
|  |  |  |  |  |  |  |  | GNAI2 | -2.02478 |  |  |
|  |  |  |  |  |  |  |  | HFE | -2.02448 |  |  |
|  |  |  |  |  |  |  |  | SPAG4 | -2.02033 |  |  |
|  |  |  |  |  |  |  |  | AASS | -2.01986 |  |  |
|  |  |  |  |  |  |  |  | CXCL12 | -2.01972 |  |  |
|  |  |  |  |  |  |  |  | RUNX1T1 | -2.01828 |  |  |
|  |  |  |  |  |  |  |  | TRAF5 | -2.01727 |  |  |
|  |  |  |  |  |  |  |  | MKNK1 | -2.0168 |  |  |
|  |  |  |  |  |  |  |  | ADCYAP1 | -2.01535 |  |  |
|  |  |  |  |  |  |  |  | AKT3 | -2.0138 |  |  |
|  |  |  |  |  |  |  |  | SULF2 | -2.01263 |  |  |
|  |  |  |  |  |  |  |  | HECTD2 | -2.01247 |  |  |
|  |  |  |  |  |  |  |  | LINC01088 | -2.01189 |  |  |
|  |  |  |  |  |  |  |  | PRR5L | -2.01172 |  |  |
|  |  |  |  |  |  |  |  | GALNS | -2.00965 |  |  |
|  |  |  |  |  |  |  |  | GGT5 | -2.00877 |  |  |
|  |  |  |  |  |  |  |  | VKORC1 | -2.00784 |  |  |
|  |  |  |  |  |  |  |  | CA5BP1 | -2.00752 |  |  |
|  |  |  |  |  |  |  |  | ERO1L | -2.005 |  |  |
|  |  |  |  |  |  |  |  | HVCN1 | -2.00405 |  |  |
|  |  |  |  |  |  |  |  | CD27 | -2.00302 |  |  |
|  |  |  |  |  |  |  |  | STEAP4 | -2.0026 |  |  |
|  |  |  |  |  |  |  |  | NAV1 | -2.00188 |  |  |
|  |  |  |  |  |  |  |  | ANGPT2 | -2.00063 |  |  |
|  |  |  |  |  |  |  |  | CALD1 | -2.00058 |  |  |
|  |  |  |  |  |  |  |  | GCNT2 | 2.001354 |  |  |
|  |  |  |  |  |  |  |  | TOLLIP | 2.001672 |  |  |
|  |  |  |  |  |  |  |  | PI16 | 2.002697 |  |  |
|  |  |  |  |  |  |  |  | PCMTD1 | 2.003345 |  |  |
|  |  |  |  |  |  |  |  | PTER | 2.00377 |  |  |
|  |  |  |  |  |  |  |  | KRTAP4-2 | 2.005051 |  |  |
|  |  |  |  |  |  |  |  | PPARA | 2.005986 |  |  |
|  |  |  |  |  |  |  |  | NMNAT3 | 2.007807 |  |  |
|  |  |  |  |  |  |  |  | TSTA3 | 2.008401 |  |  |
|  |  |  |  |  |  |  |  | KIAA1468 | 2.009309 |  |  |
|  |  |  |  |  |  |  |  | CHRM3 | 2.013791 |  |  |
|  |  |  |  |  |  |  |  | SIM1 | 2.016296 |  |  |
|  |  |  |  |  |  |  |  | ANKRD50 | 2.016691 |  |  |
|  |  |  |  |  |  |  |  | ALG9 | 2.019077 |  |  |
|  |  |  |  |  |  |  |  | FAM120AOS | 2.020892 |  |  |
|  |  |  |  |  |  |  |  | HSPA2 | 2.021693 |  |  |
|  |  |  |  |  |  |  |  | KRTAP4-8 | 2.022328 |  |  |
|  |  |  |  |  |  |  |  | CNIH4 | 2.024005 |  |  |
|  |  |  |  |  |  |  |  | MCUR1 | 2.026813 |  |  |
|  |  |  |  |  |  |  |  | RERGL | 2.027293 |  |  |
|  |  |  |  |  |  |  |  | S100A3 | 2.029059 |  |  |
|  |  |  |  |  |  |  |  | SH2D3A | 2.029306 |  |  |
|  |  |  |  |  |  |  |  | STK39 | 2.029784 |  |  |
|  |  |  |  |  |  |  |  | GPATCH4 | 2.029886 |  |  |
|  |  |  |  |  |  |  |  | RMND5A | 2.030055 |  |  |
|  |  |  |  |  |  |  |  | GSTZ1 | 2.030541 |  |  |
|  |  |  |  |  |  |  |  | ZBTB21 | 2.031801 |  |  |
|  |  |  |  |  |  |  |  | HN1L | 2.031899 |  |  |
|  |  |  |  |  |  |  |  | LARP4 | 2.032127 |  |  |
|  |  |  |  |  |  |  |  | EEF2K | 2.032589 |  |  |
|  |  |  |  |  |  |  |  | MYO19 | 2.03303 |  |  |
|  |  |  |  |  |  |  |  | KLF8 | 2.033095 |  |  |
|  |  |  |  |  |  |  |  | HOXC13 | 2.033226 |  |  |
|  |  |  |  |  |  |  |  | DUS4L | 2.035844 |  |  |
|  |  |  |  |  |  |  |  | MCOLN3 | 2.036252 |  |  |
|  |  |  |  |  |  |  |  | WDFY1 | 2.03635 |  |  |
|  |  |  |  |  |  |  |  | TANC2 | 2.037387 |  |  |
|  |  |  |  |  |  |  |  | LANCL1 | 2.037862 |  |  |
|  |  |  |  |  |  |  |  | ZCCHC2 | 2.038013 |  |  |
|  |  |  |  |  |  |  |  | RPP40 | 2.040485 |  |  |
|  |  |  |  |  |  |  |  | MFN2 | 2.041086 |  |  |
|  |  |  |  |  |  |  |  | N4BP1 | 2.04239 |  |  |
|  |  |  |  |  |  |  |  | SNCA | 2.042905 |  |  |
|  |  |  |  |  |  |  |  | PNPLA4 | 2.045179 |  |  |
|  |  |  |  |  |  |  |  | LPAR2 | 2.045568 |  |  |
|  |  |  |  |  |  |  |  | INTS6 | 2.046714 |  |  |
|  |  |  |  |  |  |  |  | BCL7A | 2.04747 |  |  |
|  |  |  |  |  |  |  |  | CARD14 | 2.047724 |  |  |
|  |  |  |  |  |  |  |  | GK5 | 2.048426 |  |  |
|  |  |  |  |  |  |  |  | CGN | 2.049005 |  |  |
|  |  |  |  |  |  |  |  | SRSF11 | 2.049136 |  |  |
|  |  |  |  |  |  |  |  | HSDL2 | 2.049283 |  |  |
|  |  |  |  |  |  |  |  | CASQ2 | 2.049318 |  |  |
|  |  |  |  |  |  |  |  | ABHD17C | 2.049923 |  |  |
|  |  |  |  |  |  |  |  | GPHN | 2.051567 |  |  |
|  |  |  |  |  |  |  |  | FBXO28 | 2.051721 |  |  |
|  |  |  |  |  |  |  |  | DUSP16 | 2.052344 |  |  |
|  |  |  |  |  |  |  |  | MSH2 | 2.052711 |  |  |
|  |  |  |  |  |  |  |  | ACSM3 | 2.054239 |  |  |
|  |  |  |  |  |  |  |  | GAREM | 2.055349 |  |  |
|  |  |  |  |  |  |  |  | STK24 | 2.055902 |  |  |
|  |  |  |  |  |  |  |  | RPRD2 | 2.058372 |  |  |
|  |  |  |  |  |  |  |  | NBEA | 2.058753 |  |  |
|  |  |  |  |  |  |  |  | AIMP1 | 2.059814 |  |  |
|  |  |  |  |  |  |  |  | SMURF1 | 2.06305 |  |  |
|  |  |  |  |  |  |  |  | ZNF343 | 2.063559 |  |  |
|  |  |  |  |  |  |  |  | MBLAC2 | 2.063916 |  |  |
|  |  |  |  |  |  |  |  | ENDOD1 | 2.063976 |  |  |
|  |  |  |  |  |  |  |  | SLC6A8 | 2.065923 |  |  |
|  |  |  |  |  |  |  |  | EIF3A | 2.068886 |  |  |
|  |  |  |  |  |  |  |  | CTTNBP2NL | 2.072104 |  |  |
|  |  |  |  |  |  |  |  | EMC3 | 2.073073 |  |  |
|  |  |  |  |  |  |  |  | MYO10 | 2.073662 |  |  |
|  |  |  |  |  |  |  |  | SSFA2 | 2.075932 |  |  |
|  |  |  |  |  |  |  |  | RALGPS1 | 2.075995 |  |  |
|  |  |  |  |  |  |  |  | LMO4 | 2.076346 |  |  |
|  |  |  |  |  |  |  |  | GABPB1-AS1 | 2.077247 |  |  |
|  |  |  |  |  |  |  |  | ANO9 | 2.078794 |  |  |
|  |  |  |  |  |  |  |  | MVK | 2.078858 |  |  |
|  |  |  |  |  |  |  |  | LUC7L3 | 2.079083 |  |  |
|  |  |  |  |  |  |  |  | USP9X | 2.07966 |  |  |
|  |  |  |  |  |  |  |  | CDC42 | 2.081066 |  |  |
|  |  |  |  |  |  |  |  | JADE1 | 2.081949 |  |  |
|  |  |  |  |  |  |  |  | TRAP1 | 2.082873 |  |  |
|  |  |  |  |  |  |  |  | LOC100288860 | 2.083783 |  |  |
|  |  |  |  |  |  |  |  | PLEKHF2 | 2.083828 |  |  |
|  |  |  |  |  |  |  |  | ASPM | 2.086293 |  |  |
|  |  |  |  |  |  |  |  | BEX5 | 2.088256 |  |  |
|  |  |  |  |  |  |  |  | HNRNPU | 2.089226 |  |  |
|  |  |  |  |  |  |  |  | LGALS8 | 2.089691 |  |  |
|  |  |  |  |  |  |  |  | DLG5 | 2.09376 |  |  |
|  |  |  |  |  |  |  |  | FOXP2 | 2.094558 |  |  |
|  |  |  |  |  |  |  |  | AGA | 2.096059 |  |  |
|  |  |  |  |  |  |  |  | RAD23B | 2.096975 |  |  |
|  |  |  |  |  |  |  |  | TTLL12 | 2.096984 |  |  |
|  |  |  |  |  |  |  |  | TRAK1 | 2.097157 |  |  |
|  |  |  |  |  |  |  |  | ZDHHC9 | 2.097507 |  |  |
|  |  |  |  |  |  |  |  | ARRDC4 | 2.097552 |  |  |
|  |  |  |  |  |  |  |  | KLK13 | 2.099303 |  |  |
|  |  |  |  |  |  |  |  | KRT9 | 2.099423 |  |  |
|  |  |  |  |  |  |  |  | MTMR1 | 2.102459 |  |  |
|  |  |  |  |  |  |  |  | PRDX2 | 2.102635 |  |  |
|  |  |  |  |  |  |  |  | DAPP1 | 2.103076 |  |  |
|  |  |  |  |  |  |  |  | FDPS | 2.103516 |  |  |
|  |  |  |  |  |  |  |  | HIF1AN | 2.1037 |  |  |
|  |  |  |  |  |  |  |  | CES4A | 2.105209 |  |  |
|  |  |  |  |  |  |  |  | GXYLT1 | 2.105643 |  |  |
|  |  |  |  |  |  |  |  | ORAI1 | 2.106326 |  |  |
|  |  |  |  |  |  |  |  | PWWP2B | 2.108089 |  |  |
|  |  |  |  |  |  |  |  | SOX21-AS1 | 2.108537 |  |  |
|  |  |  |  |  |  |  |  | MCCC1 | 2.108992 |  |  |
|  |  |  |  |  |  |  |  | GLDN | 2.109371 |  |  |
|  |  |  |  |  |  |  |  | NIPAL3 | 2.109827 |  |  |
|  |  |  |  |  |  |  |  | GATB | 2.110173 |  |  |
|  |  |  |  |  |  |  |  | STRN | 2.111489 |  |  |
|  |  |  |  |  |  |  |  | IDI1 | 2.112311 |  |  |
|  |  |  |  |  |  |  |  | ANKRD35 | 2.116965 |  |  |
|  |  |  |  |  |  |  |  | HILPDA | 2.117031 |  |  |
|  |  |  |  |  |  |  |  | WNT7B | 2.117492 |  |  |
|  |  |  |  |  |  |  |  | UTY | 2.118138 |  |  |
|  |  |  |  |  |  |  |  | RFC1 | 2.11831 |  |  |
|  |  |  |  |  |  |  |  | MYO6 | 2.120894 |  |  |
|  |  |  |  |  |  |  |  | LTN1 | 2.121059 |  |  |
|  |  |  |  |  |  |  |  | LCN2 | 2.121976 |  |  |
|  |  |  |  |  |  |  |  | SLC11A2 | 2.121999 |  |  |
|  |  |  |  |  |  |  |  | TRMT13 | 2.12229 |  |  |
|  |  |  |  |  |  |  |  | KRTAP4-4 | 2.123622 |  |  |
|  |  |  |  |  |  |  |  | ZNF398 | 2.125004 |  |  |
|  |  |  |  |  |  |  |  | KPNA1 | 2.125132 |  |  |
|  |  |  |  |  |  |  |  | RAB3B | 2.126457 |  |  |
|  |  |  |  |  |  |  |  | SEMA4A | 2.127289 |  |  |
|  |  |  |  |  |  |  |  | SLC16A7 | 2.128253 |  |  |
|  |  |  |  |  |  |  |  | GAMT | 2.128343 |  |  |
|  |  |  |  |  |  |  |  | CDC20 | 2.128448 |  |  |
|  |  |  |  |  |  |  |  | MAOA | 2.129185 |  |  |
|  |  |  |  |  |  |  |  | ACACA | 2.129579 |  |  |
|  |  |  |  |  |  |  |  | VSIG10 | 2.129986 |  |  |
|  |  |  |  |  |  |  |  | SLC27A4 | 2.130726 |  |  |
|  |  |  |  |  |  |  |  | SH3BGRL2 | 2.131009 |  |  |
|  |  |  |  |  |  |  |  | ABCA5 | 2.132659 |  |  |
|  |  |  |  |  |  |  |  | ARFGEF1 | 2.133059 |  |  |
|  |  |  |  |  |  |  |  | DHRS1 | 2.133202 |  |  |
|  |  |  |  |  |  |  |  | KPNA4 | 2.135968 |  |  |
|  |  |  |  |  |  |  |  | NFX1 | 2.138173 |  |  |
|  |  |  |  |  |  |  |  | BCAP29 | 2.14156 |  |  |
|  |  |  |  |  |  |  |  | KAT7 | 2.142982 |  |  |
|  |  |  |  |  |  |  |  | ADRB2 | 2.147118 |  |  |
|  |  |  |  |  |  |  |  | NUDT7 | 2.147283 |  |  |
|  |  |  |  |  |  |  |  | CKB | 2.152548 |  |  |
|  |  |  |  |  |  |  |  | PDE9A | 2.15267 |  |  |
|  |  |  |  |  |  |  |  | KDM5B | 2.153374 |  |  |
|  |  |  |  |  |  |  |  | FST | 2.153904 |  |  |
|  |  |  |  |  |  |  |  | WEE1 | 2.154405 |  |  |
|  |  |  |  |  |  |  |  | SPEN | 2.155789 |  |  |
|  |  |  |  |  |  |  |  | C11orf80 | 2.15637 |  |  |
|  |  |  |  |  |  |  |  | SERBP1 | 2.157076 |  |  |
|  |  |  |  |  |  |  |  | NTN1 | 2.158047 |  |  |
|  |  |  |  |  |  |  |  | STAC2 | 2.159743 |  |  |
|  |  |  |  |  |  |  |  | C10orf99 | 2.160865 |  |  |
|  |  |  |  |  |  |  |  | SLCO4C1 | 2.162021 |  |  |
|  |  |  |  |  |  |  |  | SCNN1G | 2.162396 |  |  |
|  |  |  |  |  |  |  |  | THUMPD3 | 2.162542 |  |  |
|  |  |  |  |  |  |  |  | JAG2 | 2.162688 |  |  |
|  |  |  |  |  |  |  |  | PPP1R14B | 2.162848 |  |  |
|  |  |  |  |  |  |  |  | UBR4 | 2.166444 |  |  |
|  |  |  |  |  |  |  |  | TOB2 | 2.170558 |  |  |
|  |  |  |  |  |  |  |  | PBX1 | 2.170595 |  |  |
|  |  |  |  |  |  |  |  | MOB3B | 2.171085 |  |  |
|  |  |  |  |  |  |  |  | CYB561D1 | 2.172757 |  |  |
|  |  |  |  |  |  |  |  | NAA30 | 2.172779 |  |  |
|  |  |  |  |  |  |  |  | ARNTL | 2.17623 |  |  |
|  |  |  |  |  |  |  |  | ZBTB5 | 2.176639 |  |  |
|  |  |  |  |  |  |  |  | SH3BP4 | 2.177612 |  |  |
|  |  |  |  |  |  |  |  | ZFY | 2.17882 |  |  |
|  |  |  |  |  |  |  |  | ABCD3 | 2.18003 |  |  |
|  |  |  |  |  |  |  |  | VRK1 | 2.1805 |  |  |
|  |  |  |  |  |  |  |  | TOX4 | 2.180606 |  |  |
|  |  |  |  |  |  |  |  | CCNF | 2.182428 |  |  |
|  |  |  |  |  |  |  |  | CCDC146 | 2.183089 |  |  |
|  |  |  |  |  |  |  |  | CENPW | 2.183137 |  |  |
|  |  |  |  |  |  |  |  | TIPRL | 2.184338 |  |  |
|  |  |  |  |  |  |  |  | ZMYND19 | 2.185128 |  |  |
|  |  |  |  |  |  |  |  | CDCA7L | 2.188548 |  |  |
|  |  |  |  |  |  |  |  | TYRO3 | 2.189863 |  |  |
|  |  |  |  |  |  |  |  | BICD2 | 2.190816 |  |  |
|  |  |  |  |  |  |  |  | LCE1E | 2.190831 |  |  |
|  |  |  |  |  |  |  |  | STYX | 2.190839 |  |  |
|  |  |  |  |  |  |  |  | AGFG2 | 2.191746 |  |  |
|  |  |  |  |  |  |  |  | USP30 | 2.192313 |  |  |
|  |  |  |  |  |  |  |  | BRCC3 | 2.19251 |  |  |
|  |  |  |  |  |  |  |  | CYB5D1 | 2.19334 |  |  |
|  |  |  |  |  |  |  |  | MBTD1 | 2.194348 |  |  |
|  |  |  |  |  |  |  |  | MFSD6 | 2.195425 |  |  |
|  |  |  |  |  |  |  |  | CCNB1IP1 | 2.197986 |  |  |
|  |  |  |  |  |  |  |  | GNAL | 2.198173 |  |  |
|  |  |  |  |  |  |  |  | RPP14 | 2.20043 |  |  |
|  |  |  |  |  |  |  |  | POLR1C | 2.200448 |  |  |
|  |  |  |  |  |  |  |  | SHB | 2.201533 |  |  |
|  |  |  |  |  |  |  |  | SMARCA4 | 2.203486 |  |  |
|  |  |  |  |  |  |  |  | GSDMC | 2.204664 |  |  |
|  |  |  |  |  |  |  |  | ATG16L2 | 2.205267 |  |  |
|  |  |  |  |  |  |  |  | MRPS9 | 2.20658 |  |  |
|  |  |  |  |  |  |  |  | SAMD5 | 2.209521 |  |  |
|  |  |  |  |  |  |  |  | PMAIP1 | 2.211815 |  |  |
|  |  |  |  |  |  |  |  | SHROOM2 | 2.212794 |  |  |
|  |  |  |  |  |  |  |  | EML4 | 2.213112 |  |  |
|  |  |  |  |  |  |  |  | CCDC126 | 2.214264 |  |  |
|  |  |  |  |  |  |  |  | MUM1 | 2.215204 |  |  |
|  |  |  |  |  |  |  |  | XRCC6BP1 | 2.215507 |  |  |
|  |  |  |  |  |  |  |  | CEP76 | 2.216751 |  |  |
|  |  |  |  |  |  |  |  | KIF13B | 2.218091 |  |  |
|  |  |  |  |  |  |  |  | PTRH1 | 2.218634 |  |  |
|  |  |  |  |  |  |  |  | MYO5C | 2.21942 |  |  |
|  |  |  |  |  |  |  |  | U2SURP | 2.219714 |  |  |
|  |  |  |  |  |  |  |  | NEO1 | 2.222716 |  |  |
|  |  |  |  |  |  |  |  | TMEM41B | 2.223538 |  |  |
|  |  |  |  |  |  |  |  | PLXNB1 | 2.223887 |  |  |
|  |  |  |  |  |  |  |  | CHCHD6 | 2.224117 |  |  |
|  |  |  |  |  |  |  |  | PCDH20 | 2.224541 |  |  |
|  |  |  |  |  |  |  |  | BTBD11 | 2.225369 |  |  |
|  |  |  |  |  |  |  |  | HOMER1 | 2.225465 |  |  |
|  |  |  |  |  |  |  |  | AXIN2 | 2.226863 |  |  |
|  |  |  |  |  |  |  |  | OSBPL2 | 2.227475 |  |  |
|  |  |  |  |  |  |  |  | KRTAP3-2 | 2.231729 |  |  |
|  |  |  |  |  |  |  |  | DCXR | 2.232893 |  |  |
|  |  |  |  |  |  |  |  | AGO3 | 2.233579 |  |  |
|  |  |  |  |  |  |  |  | MYLK | 2.236966 |  |  |
|  |  |  |  |  |  |  |  | MED1 | 2.237298 |  |  |
|  |  |  |  |  |  |  |  | LEPREL1 | 2.23808 |  |  |
|  |  |  |  |  |  |  |  | SLC14A1 | 2.238396 |  |  |
|  |  |  |  |  |  |  |  | LOC100506990 | 2.241032 |  |  |
|  |  |  |  |  |  |  |  | CYCS | 2.242165 |  |  |
|  |  |  |  |  |  |  |  | FEM1A | 2.242882 |  |  |
|  |  |  |  |  |  |  |  | SCD | 2.244105 |  |  |
|  |  |  |  |  |  |  |  | ZNF273 | 2.244394 |  |  |
|  |  |  |  |  |  |  |  | PAMR1 | 2.244664 |  |  |
|  |  |  |  |  |  |  |  | GPR155 | 2.244987 |  |  |
|  |  |  |  |  |  |  |  | TMEM68 | 2.245298 |  |  |
|  |  |  |  |  |  |  |  | CCND1 | 2.249434 |  |  |
|  |  |  |  |  |  |  |  | HAO2 | 2.251121 |  |  |
|  |  |  |  |  |  |  |  | PHOSPHO2 | 2.25138 |  |  |
|  |  |  |  |  |  |  |  | C12orf29 | 2.253122 |  |  |
|  |  |  |  |  |  |  |  | SMPDL3A | 2.2556 |  |  |
|  |  |  |  |  |  |  |  | GPD1L | 2.257471 |  |  |
|  |  |  |  |  |  |  |  | KIF11 | 2.2579 |  |  |
|  |  |  |  |  |  |  |  | GRPEL1 | 2.257924 |  |  |
|  |  |  |  |  |  |  |  | ARHGEF7 | 2.258532 |  |  |
|  |  |  |  |  |  |  |  | GINS1 | 2.258715 |  |  |
|  |  |  |  |  |  |  |  | SP6 | 2.259054 |  |  |
|  |  |  |  |  |  |  |  | MTURN | 2.259143 |  |  |
|  |  |  |  |  |  |  |  | ALS2CL | 2.259144 |  |  |
|  |  |  |  |  |  |  |  | RNF141 | 2.260064 |  |  |
|  |  |  |  |  |  |  |  | CCDC85C | 2.263442 |  |  |
|  |  |  |  |  |  |  |  | CPEB4 | 2.263571 |  |  |
|  |  |  |  |  |  |  |  | ERMP1 | 2.264188 |  |  |
|  |  |  |  |  |  |  |  | MEIS1 | 2.265488 |  |  |
|  |  |  |  |  |  |  |  | SLC13A2 | 2.265707 |  |  |
|  |  |  |  |  |  |  |  | CMTM4 | 2.268459 |  |  |
|  |  |  |  |  |  |  |  | RAB11A | 2.26969 |  |  |
|  |  |  |  |  |  |  |  | DAAM1 | 2.269803 |  |  |
|  |  |  |  |  |  |  |  | ID4 | 2.271076 |  |  |
|  |  |  |  |  |  |  |  | TMCC3 | 2.272863 |  |  |
|  |  |  |  |  |  |  |  | CLUH | 2.274549 |  |  |
|  |  |  |  |  |  |  |  | NT5DC1 | 2.275538 |  |  |
|  |  |  |  |  |  |  |  | EXOC6B | 2.277614 |  |  |
|  |  |  |  |  |  |  |  | PMVK | 2.278582 |  |  |
|  |  |  |  |  |  |  |  | CD1C | 2.279067 |  |  |
|  |  |  |  |  |  |  |  | SBNO1 | 2.279078 |  |  |
|  |  |  |  |  |  |  |  | PSG4 | 2.279233 |  |  |
|  |  |  |  |  |  |  |  | AGBL5 | 2.280097 |  |  |
|  |  |  |  |  |  |  |  | ATP5S | 2.281552 |  |  |
|  |  |  |  |  |  |  |  | TRIM33 | 2.281793 |  |  |
|  |  |  |  |  |  |  |  | IRX4 | 2.282649 |  |  |
|  |  |  |  |  |  |  |  | MACC1 | 2.284138 |  |  |
|  |  |  |  |  |  |  |  | F11R | 2.284563 |  |  |
|  |  |  |  |  |  |  |  | ESYT3 | 2.285351 |  |  |
|  |  |  |  |  |  |  |  | RAP1GDS1 | 2.286047 |  |  |
|  |  |  |  |  |  |  |  | ZDHHC3 | 2.287531 |  |  |
|  |  |  |  |  |  |  |  | ESPN | 2.287938 |  |  |
|  |  |  |  |  |  |  |  | TFAM | 2.288693 |  |  |
|  |  |  |  |  |  |  |  | PSMB4 | 2.289575 |  |  |
|  |  |  |  |  |  |  |  | ZNF256 | 2.289704 |  |  |
|  |  |  |  |  |  |  |  | AGPAT9 | 2.294103 |  |  |
|  |  |  |  |  |  |  |  | WASL | 2.295098 |  |  |
|  |  |  |  |  |  |  |  | GTF3C4 | 2.299634 |  |  |
|  |  |  |  |  |  |  |  | COX7B | 2.300374 |  |  |
|  |  |  |  |  |  |  |  | ZBTB7C | 2.301686 |  |  |
|  |  |  |  |  |  |  |  | RPS6KB2 | 2.304044 |  |  |
|  |  |  |  |  |  |  |  | QSOX1 | 2.306436 |  |  |
|  |  |  |  |  |  |  |  | EPHA1 | 2.306694 |  |  |
|  |  |  |  |  |  |  |  | DTNA | 2.31161 |  |  |
|  |  |  |  |  |  |  |  | FMO5 | 2.311914 |  |  |
|  |  |  |  |  |  |  |  | AMOTL1 | 2.312267 |  |  |
|  |  |  |  |  |  |  |  | GOT2 | 2.315392 |  |  |
|  |  |  |  |  |  |  |  | MYH11 | 2.318436 |  |  |
|  |  |  |  |  |  |  |  | MIB2 | 2.320031 |  |  |
|  |  |  |  |  |  |  |  | MACROD1 | 2.321082 |  |  |
|  |  |  |  |  |  |  |  | TOB1 | 2.321276 |  |  |
|  |  |  |  |  |  |  |  | INSR | 2.322355 |  |  |
|  |  |  |  |  |  |  |  | GLE1 | 2.322425 |  |  |
|  |  |  |  |  |  |  |  | PPFIBP2 | 2.322754 |  |  |
|  |  |  |  |  |  |  |  | SREK1IP1 | 2.323549 |  |  |
|  |  |  |  |  |  |  |  | BAG4 | 2.325723 |  |  |
|  |  |  |  |  |  |  |  | GPM6B | 2.326419 |  |  |
|  |  |  |  |  |  |  |  | ERBB2 | 2.331053 |  |  |
|  |  |  |  |  |  |  |  | KDM5A | 2.332202 |  |  |
|  |  |  |  |  |  |  |  | PGAP2 | 2.33318 |  |  |
|  |  |  |  |  |  |  |  | LINC01305 | 2.333272 |  |  |
|  |  |  |  |  |  |  |  | RNPC3 | 2.335964 |  |  |
|  |  |  |  |  |  |  |  | CIART | 2.336165 |  |  |
|  |  |  |  |  |  |  |  | C14orf28 | 2.339877 |  |  |
|  |  |  |  |  |  |  |  | EGFR | 2.339995 |  |  |
|  |  |  |  |  |  |  |  | PPID | 2.340342 |  |  |
|  |  |  |  |  |  |  |  | COPG2 | 2.347593 |  |  |
|  |  |  |  |  |  |  |  | PTTG1 | 2.347606 |  |  |
|  |  |  |  |  |  |  |  | ADNP2 | 2.347782 |  |  |
|  |  |  |  |  |  |  |  | UCHL3 | 2.348973 |  |  |
|  |  |  |  |  |  |  |  | SYNE2 | 2.3496 |  |  |
|  |  |  |  |  |  |  |  | KRTAP4-3 | 2.352452 |  |  |
|  |  |  |  |  |  |  |  | SERPINB8 | 2.352996 |  |  |
|  |  |  |  |  |  |  |  | MCF2L | 2.354299 |  |  |
|  |  |  |  |  |  |  |  | COLCA1 | 2.354666 |  |  |
|  |  |  |  |  |  |  |  | DMTN | 2.354919 |  |  |
|  |  |  |  |  |  |  |  | UBAP2 | 2.358265 |  |  |
|  |  |  |  |  |  |  |  | EIF2S3 | 2.361407 |  |  |
|  |  |  |  |  |  |  |  | IRX3 | 2.364971 |  |  |
|  |  |  |  |  |  |  |  | TCEA3 | 2.365548 |  |  |
|  |  |  |  |  |  |  |  | LOC100287387 | 2.365718 |  |  |
|  |  |  |  |  |  |  |  | GYLTL1B | 2.367657 |  |  |
|  |  |  |  |  |  |  |  | CYB5A | 2.368015 |  |  |
|  |  |  |  |  |  |  |  | C2orf88 | 2.368387 |  |  |
|  |  |  |  |  |  |  |  | PPIP5K1 | 2.368755 |  |  |
|  |  |  |  |  |  |  |  | ATG10 | 2.369751 |  |  |
|  |  |  |  |  |  |  |  | NAB1 | 2.370123 |  |  |
|  |  |  |  |  |  |  |  | GDPD2 | 2.370784 |  |  |
|  |  |  |  |  |  |  |  | XKRX | 2.372698 |  |  |
|  |  |  |  |  |  |  |  | PGRMC2 | 2.372882 |  |  |
|  |  |  |  |  |  |  |  | TCEB3 | 2.373087 |  |  |
|  |  |  |  |  |  |  |  | PCSK1N | 2.373788 |  |  |
|  |  |  |  |  |  |  |  | LOC100129550 | 2.374135 |  |  |
|  |  |  |  |  |  |  |  | GSK3B | 2.375709 |  |  |
|  |  |  |  |  |  |  |  | ETV3 | 2.375812 |  |  |
|  |  |  |  |  |  |  |  | ZNF544 | 2.376335 |  |  |
|  |  |  |  |  |  |  |  | USP47 | 2.377917 |  |  |
|  |  |  |  |  |  |  |  | KCNJ2 | 2.378923 |  |  |
|  |  |  |  |  |  |  |  | JMY | 2.379755 |  |  |
|  |  |  |  |  |  |  |  | RAI2 | 2.38098 |  |  |
|  |  |  |  |  |  |  |  | OXR1 | 2.382328 |  |  |
|  |  |  |  |  |  |  |  | TRIB3 | 2.383137 |  |  |
|  |  |  |  |  |  |  |  | HBB | 2.383143 |  |  |
|  |  |  |  |  |  |  |  | COBLL1 | 2.385126 |  |  |
|  |  |  |  |  |  |  |  | ITPR2 | 2.387394 |  |  |
|  |  |  |  |  |  |  |  | MBP | 2.393321 |  |  |
|  |  |  |  |  |  |  |  | SMAD5 | 2.395242 |  |  |
|  |  |  |  |  |  |  |  | KMT2C | 2.395837 |  |  |
|  |  |  |  |  |  |  |  | POLR3B | 2.396514 |  |  |
|  |  |  |  |  |  |  |  | MEGF9 | 2.396812 |  |  |
|  |  |  |  |  |  |  |  | THSD4 | 2.400855 |  |  |
|  |  |  |  |  |  |  |  | ZNF662 | 2.401706 |  |  |
|  |  |  |  |  |  |  |  | ZBTB33 | 2.402116 |  |  |
|  |  |  |  |  |  |  |  | HEBP2 | 2.405367 |  |  |
|  |  |  |  |  |  |  |  | MYH10 | 2.405965 |  |  |
|  |  |  |  |  |  |  |  | HDAC11 | 2.406384 |  |  |
|  |  |  |  |  |  |  |  | IGF1R | 2.406956 |  |  |
|  |  |  |  |  |  |  |  | GLDC | 2.407183 |  |  |
|  |  |  |  |  |  |  |  | PALB2 | 2.407186 |  |  |
|  |  |  |  |  |  |  |  | NBEAL1 | 2.407228 |  |  |
|  |  |  |  |  |  |  |  | EHBP1 | 2.407857 |  |  |
|  |  |  |  |  |  |  |  | TMEM125 | 2.408211 |  |  |
|  |  |  |  |  |  |  |  | DEGS1 | 2.410788 |  |  |
|  |  |  |  |  |  |  |  | URI1 | 2.412074 |  |  |
|  |  |  |  |  |  |  |  | GEMIN4 | 2.41323 |  |  |
|  |  |  |  |  |  |  |  | TAF1B | 2.413336 |  |  |
|  |  |  |  |  |  |  |  | SLC6A9 | 2.413434 |  |  |
|  |  |  |  |  |  |  |  | LYSMD4 | 2.420133 |  |  |
|  |  |  |  |  |  |  |  | GSTA4 | 2.421607 |  |  |
|  |  |  |  |  |  |  |  | C2orf54 | 2.422613 |  |  |
|  |  |  |  |  |  |  |  | FARP2 | 2.423363 |  |  |
|  |  |  |  |  |  |  |  | HMGA1 | 2.425847 |  |  |
|  |  |  |  |  |  |  |  | AK7 | 2.426517 |  |  |
|  |  |  |  |  |  |  |  | KRTAP9-8 | 2.426854 |  |  |
|  |  |  |  |  |  |  |  | MAPT | 2.428006 |  |  |
|  |  |  |  |  |  |  |  | HOXC10 | 2.429388 |  |  |
|  |  |  |  |  |  |  |  | AQP9 | 2.431331 |  |  |
|  |  |  |  |  |  |  |  | ZDHHC13 | 2.432008 |  |  |
|  |  |  |  |  |  |  |  | EFNA1 | 2.434523 |  |  |
|  |  |  |  |  |  |  |  | IKZF2 | 2.438286 |  |  |
|  |  |  |  |  |  |  |  | ZNF652 | 2.439389 |  |  |
|  |  |  |  |  |  |  |  | TTC39B | 2.442754 |  |  |
|  |  |  |  |  |  |  |  | KREMEN1 | 2.451478 |  |  |
|  |  |  |  |  |  |  |  | FAAH2 | 2.451725 |  |  |
|  |  |  |  |  |  |  |  | SETD8 | 2.45319 |  |  |
|  |  |  |  |  |  |  |  | DLEU1 | 2.454157 |  |  |
|  |  |  |  |  |  |  |  | USP31 | 2.455086 |  |  |
|  |  |  |  |  |  |  |  | LMTK2 | 2.456382 |  |  |
|  |  |  |  |  |  |  |  | NSDHL | 2.456559 |  |  |
|  |  |  |  |  |  |  |  | SCIN | 2.45785 |  |  |
|  |  |  |  |  |  |  |  | DLX5 | 2.459395 |  |  |
|  |  |  |  |  |  |  |  | MAL | 2.461261 |  |  |
|  |  |  |  |  |  |  |  | FAM212A | 2.464182 |  |  |
|  |  |  |  |  |  |  |  | BAIAP2 | 2.466021 |  |  |
|  |  |  |  |  |  |  |  | ZNF33B | 2.466793 |  |  |
|  |  |  |  |  |  |  |  | STK26 | 2.468401 |  |  |
|  |  |  |  |  |  |  |  | SUDS3 | 2.468793 |  |  |
|  |  |  |  |  |  |  |  | FBXL20 | 2.471272 |  |  |
|  |  |  |  |  |  |  |  | SLC39A6 | 2.471396 |  |  |
|  |  |  |  |  |  |  |  | DLL1 | 2.472528 |  |  |
|  |  |  |  |  |  |  |  | ATL2 | 2.472949 |  |  |
|  |  |  |  |  |  |  |  | DANCR | 2.473826 |  |  |
|  |  |  |  |  |  |  |  | C14orf1 | 2.475246 |  |  |
|  |  |  |  |  |  |  |  | TYR | 2.475868 |  |  |
|  |  |  |  |  |  |  |  | KLK6 | 2.476125 |  |  |
|  |  |  |  |  |  |  |  | BCKDHB | 2.478723 |  |  |
|  |  |  |  |  |  |  |  | AGR3 | 2.478926 |  |  |
|  |  |  |  |  |  |  |  | MBOAT2 | 2.479459 |  |  |
|  |  |  |  |  |  |  |  | KRTAP4-12 | 2.481328 |  |  |
|  |  |  |  |  |  |  |  | ACADL | 2.486257 |  |  |
|  |  |  |  |  |  |  |  | RGMB | 2.486885 |  |  |
|  |  |  |  |  |  |  |  | TNNT1 | 2.488405 |  |  |
|  |  |  |  |  |  |  |  | EPRS | 2.488688 |  |  |
|  |  |  |  |  |  |  |  | ST7L | 2.489315 |  |  |
|  |  |  |  |  |  |  |  | VPS13D | 2.491494 |  |  |
|  |  |  |  |  |  |  |  | TST | 2.493426 |  |  |
|  |  |  |  |  |  |  |  | SAMD12 | 2.494707 |  |  |
|  |  |  |  |  |  |  |  | PTPN4 | 2.496546 |  |  |
|  |  |  |  |  |  |  |  | F2RL2 | 2.496898 |  |  |
|  |  |  |  |  |  |  |  | SLC27A2 | 2.498452 |  |  |
|  |  |  |  |  |  |  |  | FHDC1 | 2.498507 |  |  |
|  |  |  |  |  |  |  |  | LONRF1 | 2.499639 |  |  |
|  |  |  |  |  |  |  |  | SECISBP2L | 2.501278 |  |  |
|  |  |  |  |  |  |  |  | GRIP1 | 2.502794 |  |  |
|  |  |  |  |  |  |  |  | SGPL1 | 2.509819 |  |  |
|  |  |  |  |  |  |  |  | RPUSD3 | 2.510483 |  |  |
|  |  |  |  |  |  |  |  | P2RX1 | 2.510969 |  |  |
|  |  |  |  |  |  |  |  | CD24 | 2.511133 |  |  |
|  |  |  |  |  |  |  |  | MTA3 | 2.512193 |  |  |
|  |  |  |  |  |  |  |  | PEX3 | 2.513807 |  |  |
|  |  |  |  |  |  |  |  | PRPS2 | 2.514242 |  |  |
|  |  |  |  |  |  |  |  | ZNF416 | 2.517521 |  |  |
|  |  |  |  |  |  |  |  | UHMK1 | 2.51773 |  |  |
|  |  |  |  |  |  |  |  | DIO2 | 2.518109 |  |  |
|  |  |  |  |  |  |  |  | TXLNGY | 2.519272 |  |  |
|  |  |  |  |  |  |  |  | FAM167A | 2.52124 |  |  |
|  |  |  |  |  |  |  |  | TJP2 | 2.523694 |  |  |
|  |  |  |  |  |  |  |  | HIST1H2AC | 2.525405 |  |  |
|  |  |  |  |  |  |  |  | PAIP1 | 2.52564 |  |  |
|  |  |  |  |  |  |  |  | MTERF2 | 2.529114 |  |  |
|  |  |  |  |  |  |  |  | KRTAP9-4 | 2.529763 |  |  |
|  |  |  |  |  |  |  |  | CLDN4 | 2.532053 |  |  |
|  |  |  |  |  |  |  |  | NFIA | 2.532494 |  |  |
|  |  |  |  |  |  |  |  | SCUBE2 | 2.533514 |  |  |
|  |  |  |  |  |  |  |  | LINC01184 | 2.533515 |  |  |
|  |  |  |  |  |  |  |  | PRKAB2 | 2.535373 |  |  |
|  |  |  |  |  |  |  |  | BRI3BP | 2.538818 |  |  |
|  |  |  |  |  |  |  |  | RBP7 | 2.541831 |  |  |
|  |  |  |  |  |  |  |  | UEVLD | 2.544214 |  |  |
|  |  |  |  |  |  |  |  | MCC | 2.545854 |  |  |
|  |  |  |  |  |  |  |  | TLCD1 | 2.548307 |  |  |
|  |  |  |  |  |  |  |  | LEP | 2.548693 |  |  |
|  |  |  |  |  |  |  |  | RANGAP1 | 2.551539 |  |  |
|  |  |  |  |  |  |  |  | NXT2 | 2.554535 |  |  |
|  |  |  |  |  |  |  |  | CYP26B1 | 2.557473 |  |  |
|  |  |  |  |  |  |  |  | ECHDC3 | 2.561992 |  |  |
|  |  |  |  |  |  |  |  | NDRG2 | 2.570718 |  |  |
|  |  |  |  |  |  |  |  | NRARP | 2.571021 |  |  |
|  |  |  |  |  |  |  |  | CLN8 | 2.571353 |  |  |
|  |  |  |  |  |  |  |  | PIK3R1 | 2.571575 |  |  |
|  |  |  |  |  |  |  |  | SORBS1 | 2.572142 |  |  |
|  |  |  |  |  |  |  |  | WFDC21P | 2.579317 |  |  |
|  |  |  |  |  |  |  |  | OCIAD2 | 2.579663 |  |  |
|  |  |  |  |  |  |  |  | FRAS1 | 2.580309 |  |  |
|  |  |  |  |  |  |  |  | PSME3 | 2.580619 |  |  |
|  |  |  |  |  |  |  |  | PGAP1 | 2.580947 |  |  |
|  |  |  |  |  |  |  |  | DCAF16 | 2.581339 |  |  |
|  |  |  |  |  |  |  |  | ZNF711 | 2.582639 |  |  |
|  |  |  |  |  |  |  |  | CMA1 | 2.582977 |  |  |
|  |  |  |  |  |  |  |  | CGNL1 | 2.584038 |  |  |
|  |  |  |  |  |  |  |  | AACS | 2.584496 |  |  |
|  |  |  |  |  |  |  |  | GPC3 | 2.586873 |  |  |
|  |  |  |  |  |  |  |  | PRR15L | 2.592438 |  |  |
|  |  |  |  |  |  |  |  | TBC1D8 | 2.59534 |  |  |
|  |  |  |  |  |  |  |  | EMP2 | 2.5997 |  |  |
|  |  |  |  |  |  |  |  | F12 | 2.602672 |  |  |
|  |  |  |  |  |  |  |  | GOLGA7B | 2.60481 |  |  |
|  |  |  |  |  |  |  |  | CSNK2A2 | 2.60795 |  |  |
|  |  |  |  |  |  |  |  | PHYHIP | 2.61236 |  |  |
|  |  |  |  |  |  |  |  | TTTY14 | 2.613237 |  |  |
|  |  |  |  |  |  |  |  | MIPEP | 2.614021 |  |  |
|  |  |  |  |  |  |  |  | RBM8A | 2.614949 |  |  |
|  |  |  |  |  |  |  |  | EPT1 | 2.620093 |  |  |
|  |  |  |  |  |  |  |  | SLC24A3 | 2.620386 |  |  |
|  |  |  |  |  |  |  |  | MST1R | 2.622022 |  |  |
|  |  |  |  |  |  |  |  | CDK6 | 2.622025 |  |  |
|  |  |  |  |  |  |  |  | PLS1 | 2.623725 |  |  |
|  |  |  |  |  |  |  |  | RHOD | 2.62484 |  |  |
|  |  |  |  |  |  |  |  | ZNF800 | 2.62536 |  |  |
|  |  |  |  |  |  |  |  | SHMT1 | 2.62563 |  |  |
|  |  |  |  |  |  |  |  | ESRRG | 2.628818 |  |  |
|  |  |  |  |  |  |  |  | MAML3 | 2.628978 |  |  |
|  |  |  |  |  |  |  |  | KIAA1671 | 2.631373 |  |  |
|  |  |  |  |  |  |  |  | CCDC113 | 2.632492 |  |  |
|  |  |  |  |  |  |  |  | POP7 | 2.634303 |  |  |
|  |  |  |  |  |  |  |  | KIAA0907 | 2.635176 |  |  |
|  |  |  |  |  |  |  |  | SH3GLB2 | 2.637628 |  |  |
|  |  |  |  |  |  |  |  | EBP | 2.639658 |  |  |
|  |  |  |  |  |  |  |  | CRABP2 | 2.640617 |  |  |
|  |  |  |  |  |  |  |  | GPD1 | 2.645562 |  |  |
|  |  |  |  |  |  |  |  | SC5D | 2.645775 |  |  |
|  |  |  |  |  |  |  |  | NELL2 | 2.646951 |  |  |
|  |  |  |  |  |  |  |  | G3BP1 | 2.647011 |  |  |
|  |  |  |  |  |  |  |  | MGST1 | 2.647981 |  |  |
|  |  |  |  |  |  |  |  | CTSG | 2.649782 |  |  |
|  |  |  |  |  |  |  |  | PVRL1 | 2.650906 |  |  |
|  |  |  |  |  |  |  |  | LRRC1 | 2.653673 |  |  |
|  |  |  |  |  |  |  |  | ALDOC | 2.655488 |  |  |
|  |  |  |  |  |  |  |  | ABLIM1 | 2.656282 |  |  |
|  |  |  |  |  |  |  |  | KIAA1467 | 2.659925 |  |  |
|  |  |  |  |  |  |  |  | TMEM116 | 2.660023 |  |  |
|  |  |  |  |  |  |  |  | CBX5 | 2.661506 |  |  |
|  |  |  |  |  |  |  |  | ALDH5A1 | 2.662978 |  |  |
|  |  |  |  |  |  |  |  | SH3YL1 | 2.664745 |  |  |
|  |  |  |  |  |  |  |  | FRZB | 2.664747 |  |  |
|  |  |  |  |  |  |  |  | LRBA | 2.667163 |  |  |
|  |  |  |  |  |  |  |  | PLA2G3 | 2.667665 |  |  |
|  |  |  |  |  |  |  |  | WIBG | 2.669333 |  |  |
|  |  |  |  |  |  |  |  | GPRIN2 | 2.670787 |  |  |
|  |  |  |  |  |  |  |  | TTC39C | 2.672503 |  |  |
|  |  |  |  |  |  |  |  | CEACAM5 | 2.675009 |  |  |
|  |  |  |  |  |  |  |  | COQ3 | 2.676506 |  |  |
|  |  |  |  |  |  |  |  | HIP1R | 2.67936 |  |  |
|  |  |  |  |  |  |  |  | FAM19A5 | 2.689887 |  |  |
|  |  |  |  |  |  |  |  | ROBO2 | 2.690508 |  |  |
|  |  |  |  |  |  |  |  | HACL1 | 2.691394 |  |  |
|  |  |  |  |  |  |  |  | PLA2G4F | 2.691876 |  |  |
|  |  |  |  |  |  |  |  | MMP28 | 2.693058 |  |  |
|  |  |  |  |  |  |  |  | USP54 | 2.6933 |  |  |
|  |  |  |  |  |  |  |  | BBIP1 | 2.693489 |  |  |
|  |  |  |  |  |  |  |  | TECR | 2.693612 |  |  |
|  |  |  |  |  |  |  |  | LSR | 2.696224 |  |  |
|  |  |  |  |  |  |  |  | HDHD3 | 2.69812 |  |  |
|  |  |  |  |  |  |  |  | S100B | 2.698128 |  |  |
|  |  |  |  |  |  |  |  | CCDC64B | 2.700438 |  |  |
|  |  |  |  |  |  |  |  | ELOVL6 | 2.706152 |  |  |
|  |  |  |  |  |  |  |  | PRODH | 2.706976 |  |  |
|  |  |  |  |  |  |  |  | CDA | 2.709855 |  |  |
|  |  |  |  |  |  |  |  | ENC1 | 2.711573 |  |  |
|  |  |  |  |  |  |  |  | ALOX12 | 2.712703 |  |  |
|  |  |  |  |  |  |  |  | SETD6 | 2.712924 |  |  |
|  |  |  |  |  |  |  |  | DACH1 | 2.713744 |  |  |
|  |  |  |  |  |  |  |  | SDC4 | 2.713789 |  |  |
|  |  |  |  |  |  |  |  | FAR2 | 2.721882 |  |  |
|  |  |  |  |  |  |  |  | NFAT5 | 2.722426 |  |  |
|  |  |  |  |  |  |  |  | PNPLA3 | 2.730939 |  |  |
|  |  |  |  |  |  |  |  | SOX6 | 2.732232 |  |  |
|  |  |  |  |  |  |  |  | SYT8 | 2.732778 |  |  |
|  |  |  |  |  |  |  |  | SPPL3 | 2.733571 |  |  |
|  |  |  |  |  |  |  |  | ADCY2 | 2.733659 |  |  |
|  |  |  |  |  |  |  |  | PPARGC1A | 2.73557 |  |  |
|  |  |  |  |  |  |  |  | UBE2QL1 | 2.737519 |  |  |
|  |  |  |  |  |  |  |  | TRIM16 | 2.7381 |  |  |
|  |  |  |  |  |  |  |  | NIPAL2 | 2.738837 |  |  |
|  |  |  |  |  |  |  |  | KIT | 2.742924 |  |  |
|  |  |  |  |  |  |  |  | WNK1 | 2.743168 |  |  |
|  |  |  |  |  |  |  |  | KRTAP1-3 | 2.743308 |  |  |
|  |  |  |  |  |  |  |  | TMPRSS11E | 2.748014 |  |  |
|  |  |  |  |  |  |  |  | HELLS | 2.748166 |  |  |
|  |  |  |  |  |  |  |  | C15orf48 | 2.748689 |  |  |
|  |  |  |  |  |  |  |  | CYB5R1 | 2.751047 |  |  |
|  |  |  |  |  |  |  |  | REEP6 | 2.751352 |  |  |
|  |  |  |  |  |  |  |  | RALGPS2 | 2.758283 |  |  |
|  |  |  |  |  |  |  |  | SLC35F2 | 2.761902 |  |  |
|  |  |  |  |  |  |  |  | RWDD2B | 2.771086 |  |  |
|  |  |  |  |  |  |  |  | ZBTB43 | 2.77474 |  |  |
|  |  |  |  |  |  |  |  | TMEM19 | 2.775744 |  |  |
|  |  |  |  |  |  |  |  | TIAM1 | 2.776002 |  |  |
|  |  |  |  |  |  |  |  | EFNA4 | 2.776985 |  |  |
|  |  |  |  |  |  |  |  | ABCA9 | 2.779153 |  |  |
|  |  |  |  |  |  |  |  | ABHD6 | 2.782648 |  |  |
|  |  |  |  |  |  |  |  | CLTB | 2.785655 |  |  |
|  |  |  |  |  |  |  |  | C1orf210 | 2.789717 |  |  |
|  |  |  |  |  |  |  |  | DGKA | 2.790805 |  |  |
|  |  |  |  |  |  |  |  | ZNRF3 | 2.799285 |  |  |
|  |  |  |  |  |  |  |  | AADAC | 2.80113 |  |  |
|  |  |  |  |  |  |  |  | CLCN3 | 2.802294 |  |  |
|  |  |  |  |  |  |  |  | BPY2 | 2.802685 |  |  |
|  |  |  |  |  |  |  |  | ACVR1B | 2.803212 |  |  |
|  |  |  |  |  |  |  |  | KRTAP4-1 | 2.803779 |  |  |
|  |  |  |  |  |  |  |  | KANK1 | 2.806434 |  |  |
|  |  |  |  |  |  |  |  | RAB11FIP1 | 2.80727 |  |  |
|  |  |  |  |  |  |  |  | EZR | 2.809464 |  |  |
|  |  |  |  |  |  |  |  | TMEM45A | 2.813602 |  |  |
|  |  |  |  |  |  |  |  | HSD3B1 | 2.814092 |  |  |
|  |  |  |  |  |  |  |  | ACSL1 | 2.816655 |  |  |
|  |  |  |  |  |  |  |  | HIST1H2BD | 2.819923 |  |  |
|  |  |  |  |  |  |  |  | FIGNL1 | 2.820423 |  |  |
|  |  |  |  |  |  |  |  | SHC3 | 2.822302 |  |  |
|  |  |  |  |  |  |  |  | TC2N | 2.822649 |  |  |
|  |  |  |  |  |  |  |  | SORD | 2.823515 |  |  |
|  |  |  |  |  |  |  |  | MAF | 2.827719 |  |  |
|  |  |  |  |  |  |  |  | WASF2 | 2.830553 |  |  |
|  |  |  |  |  |  |  |  | PADI2 | 2.832333 |  |  |
|  |  |  |  |  |  |  |  | TMEM117 | 2.834673 |  |  |
|  |  |  |  |  |  |  |  | EIF1AY | 2.834956 |  |  |
|  |  |  |  |  |  |  |  | ACAT2 | 2.8443 |  |  |
|  |  |  |  |  |  |  |  | INPP5A | 2.84799 |  |  |
|  |  |  |  |  |  |  |  | AIM1 | 2.8498 |  |  |
|  |  |  |  |  |  |  |  | AGR2 | 2.851503 |  |  |
|  |  |  |  |  |  |  |  | PPP1R13B | 2.855559 |  |  |
|  |  |  |  |  |  |  |  | FJX1 | 2.856121 |  |  |
|  |  |  |  |  |  |  |  | NAP1L2 | 2.857009 |  |  |
|  |  |  |  |  |  |  |  | THRB | 2.864392 |  |  |
|  |  |  |  |  |  |  |  | MID2 | 2.864693 |  |  |
|  |  |  |  |  |  |  |  | TMEM255A | 2.869746 |  |  |
|  |  |  |  |  |  |  |  | NKTR | 2.871636 |  |  |
|  |  |  |  |  |  |  |  | LEPR | 2.875748 |  |  |
|  |  |  |  |  |  |  |  | HPGD | 2.879799 |  |  |
|  |  |  |  |  |  |  |  | MMP7 | 2.880909 |  |  |
|  |  |  |  |  |  |  |  | PHLDB3 | 2.881227 |  |  |
|  |  |  |  |  |  |  |  | GPR157 | 2.881468 |  |  |
|  |  |  |  |  |  |  |  | KIF1C | 2.882722 |  |  |
|  |  |  |  |  |  |  |  | HES1 | 2.890184 |  |  |
|  |  |  |  |  |  |  |  | WFDC3 | 2.896485 |  |  |
|  |  |  |  |  |  |  |  | ITGB6 | 2.897812 |  |  |
|  |  |  |  |  |  |  |  | IL1RN | 2.899444 |  |  |
|  |  |  |  |  |  |  |  | MTHFD2L | 2.901087 |  |  |
|  |  |  |  |  |  |  |  | CCL21 | 2.901707 |  |  |
|  |  |  |  |  |  |  |  | NCALD | 2.909383 |  |  |
|  |  |  |  |  |  |  |  | ITGB4 | 2.921546 |  |  |
|  |  |  |  |  |  |  |  | POGK | 2.924251 |  |  |
|  |  |  |  |  |  |  |  | TMEM54 | 2.927522 |  |  |
|  |  |  |  |  |  |  |  | PLD1 | 2.928734 |  |  |
|  |  |  |  |  |  |  |  | LRRC8B | 2.930662 |  |  |
|  |  |  |  |  |  |  |  | FAM213A | 2.933529 |  |  |
|  |  |  |  |  |  |  |  | PTPLB | 2.934673 |  |  |
|  |  |  |  |  |  |  |  | PLA2R1 | 2.935189 |  |  |
|  |  |  |  |  |  |  |  | AGO1 | 2.937244 |  |  |
|  |  |  |  |  |  |  |  | NEGR1 | 2.937331 |  |  |
|  |  |  |  |  |  |  |  | SUPT16H | 2.938434 |  |  |
|  |  |  |  |  |  |  |  | MOXD1 | 2.938777 |  |  |
|  |  |  |  |  |  |  |  | NMRAL1 | 2.939007 |  |  |
|  |  |  |  |  |  |  |  | CTDSPL | 2.943317 |  |  |
|  |  |  |  |  |  |  |  | KRT35 | 2.943349 |  |  |
|  |  |  |  |  |  |  |  | PTCD2 | 2.94503 |  |  |
|  |  |  |  |  |  |  |  | VIPR1 | 2.946932 |  |  |
|  |  |  |  |  |  |  |  | SERHL2 | 2.947269 |  |  |
|  |  |  |  |  |  |  |  | TWIST2 | 2.949096 |  |  |
|  |  |  |  |  |  |  |  | CCDC3 | 2.952681 |  |  |
|  |  |  |  |  |  |  |  | SSX2IP | 2.953243 |  |  |
|  |  |  |  |  |  |  |  | TMC5 | 2.955502 |  |  |
|  |  |  |  |  |  |  |  | BTC | 2.958467 |  |  |
|  |  |  |  |  |  |  |  | TMEM237 | 2.963613 |  |  |
|  |  |  |  |  |  |  |  | LAMP3 | 2.965619 |  |  |
|  |  |  |  |  |  |  |  | CCL28 | 2.965826 |  |  |
|  |  |  |  |  |  |  |  | CLEC2B | 2.970111 |  |  |
|  |  |  |  |  |  |  |  | ADORA2B | 2.972039 |  |  |
|  |  |  |  |  |  |  |  | RCAN3 | 2.9739 |  |  |
|  |  |  |  |  |  |  |  | SPTSSB | 2.981033 |  |  |
|  |  |  |  |  |  |  |  | AP1M2 | 2.981399 |  |  |
|  |  |  |  |  |  |  |  | BLMH | 2.98295 |  |  |
|  |  |  |  |  |  |  |  | DEFB1 | 2.98461 |  |  |
|  |  |  |  |  |  |  |  | CDCP1 | 2.990197 |  |  |
|  |  |  |  |  |  |  |  | OVOL2 | 2.990827 |  |  |
|  |  |  |  |  |  |  |  | PROM1 | 2.991033 |  |  |
|  |  |  |  |  |  |  |  | PEX7 | 2.99204 |  |  |
|  |  |  |  |  |  |  |  | RPS15A | 2.992797 |  |  |
|  |  |  |  |  |  |  |  | BCL2 | 2.994398 |  |  |
|  |  |  |  |  |  |  |  | CYP4F12 | 2.995284 |  |  |
|  |  |  |  |  |  |  |  | DYNC1H1 | 2.998724 |  |  |
|  |  |  |  |  |  |  |  | TAOK1 | 2.999696 |  |  |
|  |  |  |  |  |  |  |  | RRM1 | 3.004627 |  |  |
|  |  |  |  |  |  |  |  | PPP1R16A | 3.004681 |  |  |
|  |  |  |  |  |  |  |  | MAP3K9 | 3.007575 |  |  |
|  |  |  |  |  |  |  |  | MICALCL | 3.0154 |  |  |
|  |  |  |  |  |  |  |  | HSPB7 | 3.018646 |  |  |
|  |  |  |  |  |  |  |  | EFHC2 | 3.025465 |  |  |
|  |  |  |  |  |  |  |  | TET3 | 3.027198 |  |  |
|  |  |  |  |  |  |  |  | RAB40C | 3.027811 |  |  |
|  |  |  |  |  |  |  |  | ZADH2 | 3.032194 |  |  |
|  |  |  |  |  |  |  |  | SLC47A1 | 3.035946 |  |  |
|  |  |  |  |  |  |  |  | ALDH3A2 | 3.040774 |  |  |
|  |  |  |  |  |  |  |  | BCL2L10 | 3.044913 |  |  |
|  |  |  |  |  |  |  |  | FAM60A | 3.054313 |  |  |
|  |  |  |  |  |  |  |  | LOC284837 | 3.055249 |  |  |
|  |  |  |  |  |  |  |  | LRRC8E | 3.059118 |  |  |
|  |  |  |  |  |  |  |  | GSTA3 | 3.060601 |  |  |
|  |  |  |  |  |  |  |  | ADTRP | 3.063162 |  |  |
|  |  |  |  |  |  |  |  | MAST4 | 3.069951 |  |  |
|  |  |  |  |  |  |  |  | KRTAP4-7 | 3.070142 |  |  |
|  |  |  |  |  |  |  |  | OSBPL6 | 3.070941 |  |  |
|  |  |  |  |  |  |  |  | KRT78 | 3.077235 |  |  |
|  |  |  |  |  |  |  |  | CA12 | 3.08028 |  |  |
|  |  |  |  |  |  |  |  | FDFT1 | 3.084026 |  |  |
|  |  |  |  |  |  |  |  | KRT33A | 3.085794 |  |  |
|  |  |  |  |  |  |  |  | STARD10 | 3.086821 |  |  |
|  |  |  |  |  |  |  |  | CBR1 | 3.087607 |  |  |
|  |  |  |  |  |  |  |  | TACC2 | 3.091884 |  |  |
|  |  |  |  |  |  |  |  | C11orf1 | 3.094197 |  |  |
|  |  |  |  |  |  |  |  | ACOX1 | 3.101764 |  |  |
|  |  |  |  |  |  |  |  | KCNK5 | 3.105097 |  |  |
|  |  |  |  |  |  |  |  | BARD1 | 3.109521 |  |  |
|  |  |  |  |  |  |  |  | GFRA1 | 3.110952 |  |  |
|  |  |  |  |  |  |  |  | FASN | 3.111887 |  |  |
|  |  |  |  |  |  |  |  | SP8 | 3.118529 |  |  |
|  |  |  |  |  |  |  |  | C9orf152 | 3.128917 |  |  |
|  |  |  |  |  |  |  |  | COCH | 3.131762 |  |  |
|  |  |  |  |  |  |  |  | CIDEC | 3.140749 |  |  |
|  |  |  |  |  |  |  |  | DCUN1D1 | 3.141782 |  |  |
|  |  |  |  |  |  |  |  | PLCXD1 | 3.145731 |  |  |
|  |  |  |  |  |  |  |  | NCS1 | 3.151612 |  |  |
|  |  |  |  |  |  |  |  | GLTP | 3.152824 |  |  |
|  |  |  |  |  |  |  |  | TMPRSS13 | 3.157861 |  |  |
|  |  |  |  |  |  |  |  | PDK3 | 3.161508 |  |  |
|  |  |  |  |  |  |  |  | ATP1B1 | 3.162488 |  |  |
|  |  |  |  |  |  |  |  | ARHGEF12 | 3.16307 |  |  |
|  |  |  |  |  |  |  |  | SLC38A1 | 3.167645 |  |  |
|  |  |  |  |  |  |  |  | TRIM59 | 3.16864 |  |  |
|  |  |  |  |  |  |  |  | KRT85 | 3.169198 |  |  |
|  |  |  |  |  |  |  |  | PLIN4 | 3.17058 |  |  |
|  |  |  |  |  |  |  |  | MCCC2 | 3.171517 |  |  |
|  |  |  |  |  |  |  |  | C3orf70 | 3.17168 |  |  |
|  |  |  |  |  |  |  |  | FAM150B | 3.17232 |  |  |
|  |  |  |  |  |  |  |  | ATP6V0A4 | 3.176542 |  |  |
|  |  |  |  |  |  |  |  | KRT74 | 3.177108 |  |  |
|  |  |  |  |  |  |  |  | EREG | 3.201843 |  |  |
|  |  |  |  |  |  |  |  | GNG12 | 3.202619 |  |  |
|  |  |  |  |  |  |  |  | MIR210HG | 3.202687 |  |  |
|  |  |  |  |  |  |  |  | LARP4B | 3.205772 |  |  |
|  |  |  |  |  |  |  |  | STON2 | 3.210866 |  |  |
|  |  |  |  |  |  |  |  | INHBB | 3.215331 |  |  |
|  |  |  |  |  |  |  |  | ACTG2 | 3.21605 |  |  |
|  |  |  |  |  |  |  |  | FCER1A | 3.216218 |  |  |
|  |  |  |  |  |  |  |  | KDM5D | 3.217703 |  |  |
|  |  |  |  |  |  |  |  | DUOXA1 | 3.21906 |  |  |
|  |  |  |  |  |  |  |  | ARID1B | 3.219924 |  |  |
|  |  |  |  |  |  |  |  | GPR27 | 3.220787 |  |  |
|  |  |  |  |  |  |  |  | MOSPD1 | 3.224081 |  |  |
|  |  |  |  |  |  |  |  | C6orf132 | 3.230792 |  |  |
|  |  |  |  |  |  |  |  | ANK3 | 3.231676 |  |  |
|  |  |  |  |  |  |  |  | GRAMD1C | 3.232593 |  |  |
|  |  |  |  |  |  |  |  | SEMA3F | 3.236427 |  |  |
|  |  |  |  |  |  |  |  | CAB39L | 3.236935 |  |  |
|  |  |  |  |  |  |  |  | RNF39 | 3.239845 |  |  |
|  |  |  |  |  |  |  |  | DHRS11 | 3.241733 |  |  |
|  |  |  |  |  |  |  |  | RASGRP1 | 3.242629 |  |  |
|  |  |  |  |  |  |  |  | SOX10 | 3.25096 |  |  |
|  |  |  |  |  |  |  |  | HECTD1 | 3.260685 |  |  |
|  |  |  |  |  |  |  |  | CHP1 | 3.264338 |  |  |
|  |  |  |  |  |  |  |  | USP9Y | 3.265879 |  |  |
|  |  |  |  |  |  |  |  | SLC16A10 | 3.271879 |  |  |
|  |  |  |  |  |  |  |  | PCSK2 | 3.280114 |  |  |
|  |  |  |  |  |  |  |  | KRTAP1-1 | 3.28129 |  |  |
|  |  |  |  |  |  |  |  | IGSF9 | 3.282918 |  |  |
|  |  |  |  |  |  |  |  | GPR126 | 3.285681 |  |  |
|  |  |  |  |  |  |  |  | SPAG1 | 3.287658 |  |  |
|  |  |  |  |  |  |  |  | CYP2J2 | 3.288881 |  |  |
|  |  |  |  |  |  |  |  | SERPINB12 | 3.290764 |  |  |
|  |  |  |  |  |  |  |  | COX15 | 3.292366 |  |  |
|  |  |  |  |  |  |  |  | TMEM33 | 3.29285 |  |  |
|  |  |  |  |  |  |  |  | TNFRSF19 | 3.296637 |  |  |
|  |  |  |  |  |  |  |  | CNN1 | 3.296681 |  |  |
|  |  |  |  |  |  |  |  | CAPN3 | 3.3016 |  |  |
|  |  |  |  |  |  |  |  | DNAJA4 | 3.303967 |  |  |
|  |  |  |  |  |  |  |  | HES2 | 3.314871 |  |  |
|  |  |  |  |  |  |  |  | PRKCZ | 3.318115 |  |  |
|  |  |  |  |  |  |  |  | NOS1 | 3.32041 |  |  |
|  |  |  |  |  |  |  |  | GAN | 3.323645 |  |  |
|  |  |  |  |  |  |  |  | C11orf71 | 3.324493 |  |  |
|  |  |  |  |  |  |  |  | TMEM99 | 3.326572 |  |  |
|  |  |  |  |  |  |  |  | LAMC3 | 3.329253 |  |  |
|  |  |  |  |  |  |  |  | SLC22A23 | 3.330944 |  |  |
|  |  |  |  |  |  |  |  | SYNGR1 | 3.334834 |  |  |
|  |  |  |  |  |  |  |  | PLIN1 | 3.337137 |  |  |
|  |  |  |  |  |  |  |  | SMAGP | 3.339353 |  |  |
|  |  |  |  |  |  |  |  | MAP3K1 | 3.339546 |  |  |
|  |  |  |  |  |  |  |  | ATP7A | 3.344488 |  |  |
|  |  |  |  |  |  |  |  | NDFIP2 | 3.34518 |  |  |
|  |  |  |  |  |  |  |  | RAB7B | 3.351283 |  |  |
|  |  |  |  |  |  |  |  | TOM1L1 | 3.356767 |  |  |
|  |  |  |  |  |  |  |  | TMEM254 | 3.358625 |  |  |
|  |  |  |  |  |  |  |  | KRTAP3-1 | 3.361877 |  |  |
|  |  |  |  |  |  |  |  | HMGCS1 | 3.363621 |  |  |
|  |  |  |  |  |  |  |  | PARD6B | 3.368122 |  |  |
|  |  |  |  |  |  |  |  | ST14 | 3.383851 |  |  |
|  |  |  |  |  |  |  |  | LAMC2 | 3.385115 |  |  |
|  |  |  |  |  |  |  |  | UNC93A | 3.389784 |  |  |
|  |  |  |  |  |  |  |  | CCNT1 | 3.390427 |  |  |
|  |  |  |  |  |  |  |  | GNAI1 | 3.39651 |  |  |
|  |  |  |  |  |  |  |  | SCN7A | 3.396996 |  |  |
|  |  |  |  |  |  |  |  | CENPU | 3.405147 |  |  |
|  |  |  |  |  |  |  |  | FBXO45 | 3.406962 |  |  |
|  |  |  |  |  |  |  |  | PAWR | 3.406969 |  |  |
|  |  |  |  |  |  |  |  | RAB27B | 3.408362 |  |  |
|  |  |  |  |  |  |  |  | COL7A1 | 3.416052 |  |  |
|  |  |  |  |  |  |  |  | C1orf21 | 3.420023 |  |  |
|  |  |  |  |  |  |  |  | AHNAK | 3.421576 |  |  |
|  |  |  |  |  |  |  |  | HSD17B8 | 3.424784 |  |  |
|  |  |  |  |  |  |  |  | HOXA1 | 3.426078 |  |  |
|  |  |  |  |  |  |  |  | QPCT | 3.431992 |  |  |
|  |  |  |  |  |  |  |  | KIAA1324 | 3.434561 |  |  |
|  |  |  |  |  |  |  |  | NR2F6 | 3.438495 |  |  |
|  |  |  |  |  |  |  |  | STX19 | 3.43975 |  |  |
|  |  |  |  |  |  |  |  | HIST2H2BE | 3.451271 |  |  |
|  |  |  |  |  |  |  |  | HIPK1 | 3.459315 |  |  |
|  |  |  |  |  |  |  |  | SLC28A3 | 3.460697 |  |  |
|  |  |  |  |  |  |  |  | SPTLC3 | 3.462648 |  |  |
|  |  |  |  |  |  |  |  | IL36RN | 3.464589 |  |  |
|  |  |  |  |  |  |  |  | RABGAP1L | 3.468431 |  |  |
|  |  |  |  |  |  |  |  | HSD11B2 | 3.471018 |  |  |
|  |  |  |  |  |  |  |  | EMX2 | 3.473603 |  |  |
|  |  |  |  |  |  |  |  | SDR42E1 | 3.485474 |  |  |
|  |  |  |  |  |  |  |  | SLC4A11 | 3.486985 |  |  |
|  |  |  |  |  |  |  |  | C12orf66 | 3.490948 |  |  |
|  |  |  |  |  |  |  |  | BMP7 | 3.492324 |  |  |
|  |  |  |  |  |  |  |  | MREG | 3.513372 |  |  |
|  |  |  |  |  |  |  |  | LIMK2 | 3.517704 |  |  |
|  |  |  |  |  |  |  |  | ARAP2 | 3.523325 |  |  |
|  |  |  |  |  |  |  |  | ACKR4 | 3.526109 |  |  |
|  |  |  |  |  |  |  |  | KRTAP4-9 | 3.533763 |  |  |
|  |  |  |  |  |  |  |  | DNASE1L2 | 3.541267 |  |  |
|  |  |  |  |  |  |  |  | PTPN21 | 3.553403 |  |  |
|  |  |  |  |  |  |  |  | TBX3 | 3.555327 |  |  |
|  |  |  |  |  |  |  |  | MSMO1 | 3.559724 |  |  |
|  |  |  |  |  |  |  |  | TPD52 | 3.563611 |  |  |
|  |  |  |  |  |  |  |  | NAV3 | 3.566488 |  |  |
|  |  |  |  |  |  |  |  | HLF | 3.568109 |  |  |
|  |  |  |  |  |  |  |  | PRIMA1 | 3.572441 |  |  |
|  |  |  |  |  |  |  |  | SLC7A1 | 3.577809 |  |  |
|  |  |  |  |  |  |  |  | TMEM56 | 3.579985 |  |  |
|  |  |  |  |  |  |  |  | TPD52L1 | 3.583747 |  |  |
|  |  |  |  |  |  |  |  | EPHB3 | 3.583951 |  |  |
|  |  |  |  |  |  |  |  | KRT8 | 3.58448 |  |  |
|  |  |  |  |  |  |  |  | ABHD5 | 3.601117 |  |  |
|  |  |  |  |  |  |  |  | SYNM | 3.61041 |  |  |
|  |  |  |  |  |  |  |  | WFDC12 | 3.614197 |  |  |
|  |  |  |  |  |  |  |  | KIF21A | 3.617487 |  |  |
|  |  |  |  |  |  |  |  | TRIM7 | 3.618111 |  |  |
|  |  |  |  |  |  |  |  | ARHGEF19 | 3.618434 |  |  |
|  |  |  |  |  |  |  |  | NPY1R | 3.622215 |  |  |
|  |  |  |  |  |  |  |  | TSPAN5 | 3.626899 |  |  |
|  |  |  |  |  |  |  |  | ATP2C2 | 3.63126 |  |  |
|  |  |  |  |  |  |  |  | OTUB2 | 3.636792 |  |  |
|  |  |  |  |  |  |  |  | MAP7 | 3.636851 |  |  |
|  |  |  |  |  |  |  |  | HMGCS2 | 3.636944 |  |  |
|  |  |  |  |  |  |  |  | RASSF6 | 3.638153 |  |  |
|  |  |  |  |  |  |  |  | BSPRY | 3.643248 |  |  |
|  |  |  |  |  |  |  |  | CXCL14 | 3.643862 |  |  |
|  |  |  |  |  |  |  |  | RAB3D | 3.650638 |  |  |
|  |  |  |  |  |  |  |  | HRASLS | 3.659547 |  |  |
|  |  |  |  |  |  |  |  | OTUB1 | 3.661819 |  |  |
|  |  |  |  |  |  |  |  | FAM162A | 3.662881 |  |  |
|  |  |  |  |  |  |  |  | LHX2 | 3.670007 |  |  |
|  |  |  |  |  |  |  |  | PITPNM3 | 3.670582 |  |  |
|  |  |  |  |  |  |  |  | LYPD5 | 3.672307 |  |  |
|  |  |  |  |  |  |  |  | TMEM159 | 3.672327 |  |  |
|  |  |  |  |  |  |  |  | YOD1 | 3.678117 |  |  |
|  |  |  |  |  |  |  |  | AHNAK2 | 3.68153 |  |  |
|  |  |  |  |  |  |  |  | F10 | 3.683071 |  |  |
|  |  |  |  |  |  |  |  | SOWAHC | 3.693958 |  |  |
|  |  |  |  |  |  |  |  | RIMS3 | 3.696692 |  |  |
|  |  |  |  |  |  |  |  | RAB3IP | 3.69694 |  |  |
|  |  |  |  |  |  |  |  | PCDH7 | 3.698164 |  |  |
|  |  |  |  |  |  |  |  | EPHB1 | 3.698911 |  |  |
|  |  |  |  |  |  |  |  | SCNN1B | 3.699898 |  |  |
|  |  |  |  |  |  |  |  | MMP27 | 3.701559 |  |  |
|  |  |  |  |  |  |  |  | BTG3 | 3.706355 |  |  |
|  |  |  |  |  |  |  |  | AR | 3.706803 |  |  |
|  |  |  |  |  |  |  |  | KRTAP1-5 | 3.719184 |  |  |
|  |  |  |  |  |  |  |  | PMEL | 3.724503 |  |  |
|  |  |  |  |  |  |  |  | SLC16A14 | 3.734956 |  |  |
|  |  |  |  |  |  |  |  | SLC15A1 | 3.738155 |  |  |
|  |  |  |  |  |  |  |  | ANKRD33B | 3.747458 |  |  |
|  |  |  |  |  |  |  |  | TSTD1 | 3.75117 |  |  |
|  |  |  |  |  |  |  |  | REEP1 | 3.756611 |  |  |
|  |  |  |  |  |  |  |  | PLEK2 | 3.759578 |  |  |
|  |  |  |  |  |  |  |  | SLC44A5 | 3.759689 |  |  |
|  |  |  |  |  |  |  |  | SOX9 | 3.764358 |  |  |
|  |  |  |  |  |  |  |  | RYR1 | 3.764559 |  |  |
|  |  |  |  |  |  |  |  | KDF1 | 3.766015 |  |  |
|  |  |  |  |  |  |  |  | SVIP | 3.78162 |  |  |
|  |  |  |  |  |  |  |  | CBLC | 3.785794 |  |  |
|  |  |  |  |  |  |  |  | PTGS1 | 3.796865 |  |  |
|  |  |  |  |  |  |  |  | UBE4B | 3.79764 |  |  |
|  |  |  |  |  |  |  |  | SLPI | 3.799124 |  |  |
|  |  |  |  |  |  |  |  | GREM2 | 3.802549 |  |  |
|  |  |  |  |  |  |  |  | UBN2 | 3.8027 |  |  |
|  |  |  |  |  |  |  |  | ELF5 | 3.820928 |  |  |
|  |  |  |  |  |  |  |  | COL4A6 | 3.821692 |  |  |
|  |  |  |  |  |  |  |  | PPL | 3.822978 |  |  |
|  |  |  |  |  |  |  |  | SPRR3 | 3.823202 |  |  |
|  |  |  |  |  |  |  |  | KRTAP3-3 | 3.823244 |  |  |
|  |  |  |  |  |  |  |  | SHROOM3 | 3.825737 |  |  |
|  |  |  |  |  |  |  |  | SGCG | 3.843846 |  |  |
|  |  |  |  |  |  |  |  | SPINT2 | 3.846823 |  |  |
|  |  |  |  |  |  |  |  | RORC | 3.865232 |  |  |
|  |  |  |  |  |  |  |  | RNF43 | 3.872362 |  |  |
|  |  |  |  |  |  |  |  | CYB5R2 | 3.875034 |  |  |
|  |  |  |  |  |  |  |  | ST6GALNAC2 | 3.88288 |  |  |
|  |  |  |  |  |  |  |  | ZC3H12C | 3.887095 |  |  |
|  |  |  |  |  |  |  |  | KRT75 | 3.890997 |  |  |
|  |  |  |  |  |  |  |  | CA2 | 3.908262 |  |  |
|  |  |  |  |  |  |  |  | KIAA1244 | 3.912942 |  |  |
|  |  |  |  |  |  |  |  | SPTBN2 | 3.916485 |  |  |
|  |  |  |  |  |  |  |  | FADS2 | 3.918828 |  |  |
|  |  |  |  |  |  |  |  | MYO5B | 3.919804 |  |  |
|  |  |  |  |  |  |  |  | RIPK4 | 3.922689 |  |  |
|  |  |  |  |  |  |  |  | PIK3C2G | 3.924615 |  |  |
|  |  |  |  |  |  |  |  | CPA4 | 3.9372 |  |  |
|  |  |  |  |  |  |  |  | GPR56 | 3.938787 |  |  |
|  |  |  |  |  |  |  |  | TGM1 | 3.940263 |  |  |
|  |  |  |  |  |  |  |  | ADIPOQ | 3.941011 |  |  |
|  |  |  |  |  |  |  |  | ACTA1 | 3.946917 |  |  |
|  |  |  |  |  |  |  |  | GATM | 3.947237 |  |  |
|  |  |  |  |  |  |  |  | APOD | 3.960588 |  |  |
|  |  |  |  |  |  |  |  | SLC2A1 | 3.964459 |  |  |
|  |  |  |  |  |  |  |  | ARHGEF28 | 3.964477 |  |  |
|  |  |  |  |  |  |  |  | SLC12A2 | 3.966116 |  |  |
|  |  |  |  |  |  |  |  | MAP2 | 3.977916 |  |  |
|  |  |  |  |  |  |  |  | C21orf91 | 3.984351 |  |  |
|  |  |  |  |  |  |  |  | IL18 | 3.989436 |  |  |
|  |  |  |  |  |  |  |  | CDH19 | 3.989553 |  |  |
|  |  |  |  |  |  |  |  | SLC39A2 | 4.009965 |  |  |
|  |  |  |  |  |  |  |  | AIM1L | 4.0114 |  |  |
|  |  |  |  |  |  |  |  | MICALL1 | 4.011484 |  |  |
|  |  |  |  |  |  |  |  | KIAA1522 | 4.01671 |  |  |
|  |  |  |  |  |  |  |  | EPHX2 | 4.022584 |  |  |
|  |  |  |  |  |  |  |  | PKIB | 4.028833 |  |  |
|  |  |  |  |  |  |  |  | TMEM246 | 4.038293 |  |  |
|  |  |  |  |  |  |  |  | KCTD15 | 4.052586 |  |  |
|  |  |  |  |  |  |  |  | TUBA4A | 4.056289 |  |  |
|  |  |  |  |  |  |  |  | EFNB2 | 4.057459 |  |  |
|  |  |  |  |  |  |  |  | TGFA | 4.058207 |  |  |
|  |  |  |  |  |  |  |  | MASP1 | 4.066709 |  |  |
|  |  |  |  |  |  |  |  | KRT10 | 4.069826 |  |  |
|  |  |  |  |  |  |  |  | EPN3 | 4.071032 |  |  |
|  |  |  |  |  |  |  |  | HOTAIR | 4.07277 |  |  |
|  |  |  |  |  |  |  |  | SMIM5 | 4.08097 |  |  |
|  |  |  |  |  |  |  |  | MAB21L3 | 4.082508 |  |  |
|  |  |  |  |  |  |  |  | BEX2 | 4.0861 |  |  |
|  |  |  |  |  |  |  |  | LPAR3 | 4.09888 |  |  |
|  |  |  |  |  |  |  |  | ITGA8 | 4.101184 |  |  |
|  |  |  |  |  |  |  |  | EIF4B | 4.105857 |  |  |
|  |  |  |  |  |  |  |  | PPAP2C | 4.117893 |  |  |
|  |  |  |  |  |  |  |  | FAM189A2 | 4.120104 |  |  |
|  |  |  |  |  |  |  |  | MFSD2A | 4.123407 |  |  |
|  |  |  |  |  |  |  |  | BDH1 | 4.128514 |  |  |
|  |  |  |  |  |  |  |  | PSAT1 | 4.146801 |  |  |
|  |  |  |  |  |  |  |  | HMGCR | 4.149866 |  |  |
|  |  |  |  |  |  |  |  | MTPAP | 4.16055 |  |  |
|  |  |  |  |  |  |  |  | IL22RA1 | 4.16094 |  |  |
|  |  |  |  |  |  |  |  | PAIP2B | 4.167981 |  |  |
|  |  |  |  |  |  |  |  | ARHGEF5 | 4.168347 |  |  |
|  |  |  |  |  |  |  |  | POU3F1 | 4.172244 |  |  |
|  |  |  |  |  |  |  |  | INADL | 4.172311 |  |  |
|  |  |  |  |  |  |  |  | ACP6 | 4.189461 |  |  |
|  |  |  |  |  |  |  |  | BAIAP2L1 | 4.190999 |  |  |
|  |  |  |  |  |  |  |  | PDZD2 | 4.191901 |  |  |
|  |  |  |  |  |  |  |  | ARHGEF26 | 4.205878 |  |  |
|  |  |  |  |  |  |  |  | KLK1 | 4.206531 |  |  |
|  |  |  |  |  |  |  |  | PANK1 | 4.209139 |  |  |
|  |  |  |  |  |  |  |  | MAPK13 | 4.211495 |  |  |
|  |  |  |  |  |  |  |  | RPTN | 4.221373 |  |  |
|  |  |  |  |  |  |  |  | GABRP | 4.223104 |  |  |
|  |  |  |  |  |  |  |  | CYP3A5 | 4.229308 |  |  |
|  |  |  |  |  |  |  |  | SQLE | 4.243857 |  |  |
|  |  |  |  |  |  |  |  | PLP1 | 4.2491 |  |  |
|  |  |  |  |  |  |  |  | GREM1 | 4.254436 |  |  |
|  |  |  |  |  |  |  |  | PRSS12 | 4.257609 |  |  |
|  |  |  |  |  |  |  |  | LMO7 | 4.257844 |  |  |
|  |  |  |  |  |  |  |  | RHBDL2 | 4.282946 |  |  |
|  |  |  |  |  |  |  |  | STAP2 | 4.288617 |  |  |
|  |  |  |  |  |  |  |  | IFNLR1 | 4.296313 |  |  |
|  |  |  |  |  |  |  |  | MACROD2 | 4.313969 |  |  |
|  |  |  |  |  |  |  |  | MYOCD | 4.318118 |  |  |
|  |  |  |  |  |  |  |  | ITGA2 | 4.32745 |  |  |
|  |  |  |  |  |  |  |  | FBXW7 | 4.331547 |  |  |
|  |  |  |  |  |  |  |  | GPAM | 4.333574 |  |  |
|  |  |  |  |  |  |  |  | SLITRK6 | 4.341847 |  |  |
|  |  |  |  |  |  |  |  | OCLN | 4.343015 |  |  |
|  |  |  |  |  |  |  |  | CELSR1 | 4.349445 |  |  |
|  |  |  |  |  |  |  |  | JUP | 4.354682 |  |  |
|  |  |  |  |  |  |  |  | CNTN3 | 4.356847 |  |  |
|  |  |  |  |  |  |  |  | HR | 4.403469 |  |  |
|  |  |  |  |  |  |  |  | ZNF57 | 4.41032 |  |  |
|  |  |  |  |  |  |  |  | C3orf52 | 4.414952 |  |  |
|  |  |  |  |  |  |  |  | HSPA4L | 4.428593 |  |  |
|  |  |  |  |  |  |  |  | PON3 | 4.43324 |  |  |
|  |  |  |  |  |  |  |  | SLC6A14 | 4.434669 |  |  |
|  |  |  |  |  |  |  |  | DDX3Y | 4.449513 |  |  |
|  |  |  |  |  |  |  |  | IL1R2 | 4.460859 |  |  |
|  |  |  |  |  |  |  |  | CORO2A | 4.46469 |  |  |
|  |  |  |  |  |  |  |  | LAMB3 | 4.465469 |  |  |
|  |  |  |  |  |  |  |  | GPT2 | 4.468821 |  |  |
|  |  |  |  |  |  |  |  | PKIA | 4.470319 |  |  |
|  |  |  |  |  |  |  |  | SPRR4 | 4.481879 |  |  |
|  |  |  |  |  |  |  |  | PLEKHH1 | 4.488593 |  |  |
|  |  |  |  |  |  |  |  | S1PR5 | 4.492914 |  |  |
|  |  |  |  |  |  |  |  | INO80D | 4.493549 |  |  |
|  |  |  |  |  |  |  |  | CD1E | 4.493559 |  |  |
|  |  |  |  |  |  |  |  | DENND2C | 4.494942 |  |  |
|  |  |  |  |  |  |  |  | DSG2 | 4.508514 |  |  |
|  |  |  |  |  |  |  |  | ELMO3 | 4.513906 |  |  |
|  |  |  |  |  |  |  |  | PM20D1 | 4.5277 |  |  |
|  |  |  |  |  |  |  |  | CYP4B1 | 4.531746 |  |  |
|  |  |  |  |  |  |  |  | WWC1 | 4.53632 |  |  |
|  |  |  |  |  |  |  |  | FAM83G | 4.538225 |  |  |
|  |  |  |  |  |  |  |  | DEGS2 | 4.546802 |  |  |
|  |  |  |  |  |  |  |  | LPHN3 | 4.552445 |  |  |
|  |  |  |  |  |  |  |  | TGM5 | 4.552505 |  |  |
|  |  |  |  |  |  |  |  | TFCP2L1 | 4.553887 |  |  |
|  |  |  |  |  |  |  |  | PHGDH | 4.556469 |  |  |
|  |  |  |  |  |  |  |  | ZNF185 | 4.563376 |  |  |
|  |  |  |  |  |  |  |  | NHSL1 | 4.568658 |  |  |
|  |  |  |  |  |  |  |  | PTBP3 | 4.579459 |  |  |
|  |  |  |  |  |  |  |  | GPC1 | 4.580644 |  |  |
|  |  |  |  |  |  |  |  | PTPRF | 4.582444 |  |  |
|  |  |  |  |  |  |  |  | PPP2R3A | 4.586046 |  |  |
|  |  |  |  |  |  |  |  | EGLN3 | 4.588572 |  |  |
|  |  |  |  |  |  |  |  | PARD3 | 4.593327 |  |  |
|  |  |  |  |  |  |  |  | ARHGEF4 | 4.617373 |  |  |
|  |  |  |  |  |  |  |  | FAM83H | 4.617482 |  |  |
|  |  |  |  |  |  |  |  | HOMER2 | 4.620243 |  |  |
|  |  |  |  |  |  |  |  | EPB41L5 | 4.625942 |  |  |
|  |  |  |  |  |  |  |  | CYSRT1 | 4.645211 |  |  |
|  |  |  |  |  |  |  |  | CNTN1 | 4.648973 |  |  |
|  |  |  |  |  |  |  |  | PRRG4 | 4.653461 |  |  |
|  |  |  |  |  |  |  |  | TF | 4.662588 |  |  |
|  |  |  |  |  |  |  |  | SDC1 | 4.667415 |  |  |
|  |  |  |  |  |  |  |  | CRISP3 | 4.667673 |  |  |
|  |  |  |  |  |  |  |  | RBP1 | 4.670634 |  |  |
|  |  |  |  |  |  |  |  | LTB4R | 4.681536 |  |  |
|  |  |  |  |  |  |  |  | EPS8L2 | 4.683515 |  |  |
|  |  |  |  |  |  |  |  | VSIG10L | 4.694329 |  |  |
|  |  |  |  |  |  |  |  | IGSF3 | 4.703537 |  |  |
|  |  |  |  |  |  |  |  | ELOVL3 | 4.723037 |  |  |
|  |  |  |  |  |  |  |  | MYCL | 4.724334 |  |  |
|  |  |  |  |  |  |  |  | TUFT1 | 4.728085 |  |  |
|  |  |  |  |  |  |  |  | CAMSAP3 | 4.72928 |  |  |
|  |  |  |  |  |  |  |  | ANXA3 | 4.740175 |  |  |
|  |  |  |  |  |  |  |  | SOX15 | 4.7465 |  |  |
|  |  |  |  |  |  |  |  | CASP14 | 4.75175 |  |  |
|  |  |  |  |  |  |  |  | LINC01133 | 4.762379 |  |  |
|  |  |  |  |  |  |  |  | ARHGAP32 | 4.765424 |  |  |
|  |  |  |  |  |  |  |  | CSTA | 4.77022 |  |  |
|  |  |  |  |  |  |  |  | FAM57A | 4.790149 |  |  |
|  |  |  |  |  |  |  |  | ALOXE3 | 4.791009 |  |  |
|  |  |  |  |  |  |  |  | IRX1 | 4.792011 |  |  |
|  |  |  |  |  |  |  |  | ELL3 | 4.796286 |  |  |
|  |  |  |  |  |  |  |  | ACPP | 4.8027 |  |  |
|  |  |  |  |  |  |  |  | EPHA4 | 4.805055 |  |  |
|  |  |  |  |  |  |  |  | MUC7 | 4.808425 |  |  |
|  |  |  |  |  |  |  |  | ANXA9 | 4.809922 |  |  |
|  |  |  |  |  |  |  |  | GPLD1 | 4.823381 |  |  |
|  |  |  |  |  |  |  |  | CTNNBIP1 | 4.82957 |  |  |
|  |  |  |  |  |  |  |  | DSC2 | 4.832973 |  |  |
|  |  |  |  |  |  |  |  | CCDC64 | 4.833882 |  |  |
|  |  |  |  |  |  |  |  | SERPINB3 | 4.835783 |  |  |
|  |  |  |  |  |  |  |  | TMEM154 | 4.848042 |  |  |
|  |  |  |  |  |  |  |  | RHPN2 | 4.861166 |  |  |
|  |  |  |  |  |  |  |  | CYP39A1 | 4.875962 |  |  |
|  |  |  |  |  |  |  |  | PTPN3 | 4.877922 |  |  |
|  |  |  |  |  |  |  |  | MYH14 | 4.883646 |  |  |
|  |  |  |  |  |  |  |  | EPHB6 | 4.885981 |  |  |
|  |  |  |  |  |  |  |  | SCGB2A1 | 4.895168 |  |  |
|  |  |  |  |  |  |  |  | PVRL4 | 4.895427 |  |  |
|  |  |  |  |  |  |  |  | S100P | 4.897885 |  |  |
|  |  |  |  |  |  |  |  | EPB41L4B | 4.90244 |  |  |
|  |  |  |  |  |  |  |  | LRP4 | 4.902671 |  |  |
|  |  |  |  |  |  |  |  | IMPA2 | 4.917871 |  |  |
|  |  |  |  |  |  |  |  | LAMA3 | 4.924662 |  |  |
|  |  |  |  |  |  |  |  | ACER1 | 4.928109 |  |  |
|  |  |  |  |  |  |  |  | DES | 4.933143 |  |  |
|  |  |  |  |  |  |  |  | RHOV | 4.94007 |  |  |
|  |  |  |  |  |  |  |  | IRX5 | 4.94067 |  |  |
|  |  |  |  |  |  |  |  | PTGER3 | 4.944525 |  |  |
|  |  |  |  |  |  |  |  | SRD5A1 | 4.94584 |  |  |
|  |  |  |  |  |  |  |  | LCE3D | 4.948171 |  |  |
|  |  |  |  |  |  |  |  | GRHL2 | 4.978891 |  |  |
|  |  |  |  |  |  |  |  | WDR72 | 4.985813 |  |  |
|  |  |  |  |  |  |  |  | LGR5 | 4.994764 |  |  |
|  |  |  |  |  |  |  |  | A2ML1 | 5.006394 |  |  |
|  |  |  |  |  |  |  |  | SLC46A2 | 5.007345 |  |  |
|  |  |  |  |  |  |  |  | PARD6G | 5.008284 |  |  |
|  |  |  |  |  |  |  |  | PPP1R13L | 5.012414 |  |  |
|  |  |  |  |  |  |  |  | BAG1 | 5.019907 |  |  |
|  |  |  |  |  |  |  |  | KRT18 | 5.03875 |  |  |
|  |  |  |  |  |  |  |  | CHL1 | 5.042871 |  |  |
|  |  |  |  |  |  |  |  | CDKN2B | 5.049791 |  |  |
|  |  |  |  |  |  |  |  | SYTL1 | 5.064931 |  |  |
|  |  |  |  |  |  |  |  | EVPL | 5.068368 |  |  |
|  |  |  |  |  |  |  |  | ELOVL7 | 5.068734 |  |  |
|  |  |  |  |  |  |  |  | TP53AIP1 | 5.072911 |  |  |
|  |  |  |  |  |  |  |  | KY | 5.078411 |  |  |
|  |  |  |  |  |  |  |  | FBXL16 | 5.082067 |  |  |
|  |  |  |  |  |  |  |  | IFFO2 | 5.091756 |  |  |
|  |  |  |  |  |  |  |  | SDR9C7 | 5.092772 |  |  |
|  |  |  |  |  |  |  |  | ALDH3A1 | 5.099599 |  |  |
|  |  |  |  |  |  |  |  | ADH1B | 5.115311 |  |  |
|  |  |  |  |  |  |  |  | GPR115 | 5.132712 |  |  |
|  |  |  |  |  |  |  |  | CXADR | 5.132907 |  |  |
|  |  |  |  |  |  |  |  | MARVELD2 | 5.135262 |  |  |
|  |  |  |  |  |  |  |  | TGM3 | 5.147991 |  |  |
|  |  |  |  |  |  |  |  | FGFR3 | 5.197727 |  |  |
|  |  |  |  |  |  |  |  | COL4A5 | 5.20009 |  |  |
|  |  |  |  |  |  |  |  | TENM2 | 5.20156 |  |  |
|  |  |  |  |  |  |  |  | AQP5 | 5.225 |  |  |
|  |  |  |  |  |  |  |  | ELOVL4 | 5.242438 |  |  |
|  |  |  |  |  |  |  |  | MIR205HG | 5.249579 |  |  |
|  |  |  |  |  |  |  |  | KRT27 | 5.260685 |  |  |
|  |  |  |  |  |  |  |  | ZG16B | 5.263966 |  |  |
|  |  |  |  |  |  |  |  | ANKRD22 | 5.281876 |  |  |
|  |  |  |  |  |  |  |  | TM7SF2 | 5.285973 |  |  |
|  |  |  |  |  |  |  |  | EFNA3 | 5.296698 |  |  |
|  |  |  |  |  |  |  |  | EN1 | 5.312822 |  |  |
|  |  |  |  |  |  |  |  | TNS4 | 5.323448 |  |  |
|  |  |  |  |  |  |  |  | PRR9 | 5.329633 |  |  |
|  |  |  |  |  |  |  |  | CIDEA | 5.332089 |  |  |
|  |  |  |  |  |  |  |  | CYP4F22 | 5.340334 |  |  |
|  |  |  |  |  |  |  |  | HLA-DQB2 | 5.350969 |  |  |
|  |  |  |  |  |  |  |  | LOC101927164 | 5.355889 |  |  |
|  |  |  |  |  |  |  |  | ATP6V1C2 | 5.3613 |  |  |
|  |  |  |  |  |  |  |  | STARD5 | 5.364629 |  |  |
|  |  |  |  |  |  |  |  | GJB6 | 5.375126 |  |  |
|  |  |  |  |  |  |  |  | CLDN10 | 5.397005 |  |  |
|  |  |  |  |  |  |  |  | MFSD4 | 5.407485 |  |  |
|  |  |  |  |  |  |  |  | VAV3 | 5.407856 |  |  |
|  |  |  |  |  |  |  |  | AJUBA | 5.411339 |  |  |
|  |  |  |  |  |  |  |  | LOC100130476 | 5.425033 |  |  |
|  |  |  |  |  |  |  |  | GSTO2 | 5.425835 |  |  |
|  |  |  |  |  |  |  |  | TMEM79 | 5.440569 |  |  |
|  |  |  |  |  |  |  |  | OVOL1 | 5.448473 |  |  |
|  |  |  |  |  |  |  |  | IVL | 5.449839 |  |  |
|  |  |  |  |  |  |  |  | RASSF10 | 5.462959 |  |  |
|  |  |  |  |  |  |  |  | KCTD1 | 5.465547 |  |  |
|  |  |  |  |  |  |  |  | DKK2 | 5.474409 |  |  |
|  |  |  |  |  |  |  |  | FCGBP | 5.488027 |  |  |
|  |  |  |  |  |  |  |  | FLRT3 | 5.497101 |  |  |
|  |  |  |  |  |  |  |  | BCHE | 5.501694 |  |  |
|  |  |  |  |  |  |  |  | HCAR3 | 5.53889 |  |  |
|  |  |  |  |  |  |  |  | SLC1A6 | 5.552849 |  |  |
|  |  |  |  |  |  |  |  | TMEM45B | 5.553732 |  |  |
|  |  |  |  |  |  |  |  | PRSS8 | 5.580079 |  |  |
|  |  |  |  |  |  |  |  | COBL | 5.600481 |  |  |
|  |  |  |  |  |  |  |  | IGFL2 | 5.602084 |  |  |
|  |  |  |  |  |  |  |  | FAM110C | 5.619861 |  |  |
|  |  |  |  |  |  |  |  | CDH3 | 5.620962 |  |  |
|  |  |  |  |  |  |  |  | BNC1 | 5.621317 |  |  |
|  |  |  |  |  |  |  |  | RORA | 5.631356 |  |  |
|  |  |  |  |  |  |  |  | FA2H | 5.643849 |  |  |
|  |  |  |  |  |  |  |  | EPCAM | 5.65229 |  |  |
|  |  |  |  |  |  |  |  | LOC284023 | 5.691909 |  |  |
|  |  |  |  |  |  |  |  | VANGL2 | 5.692329 |  |  |
|  |  |  |  |  |  |  |  | ENDOU | 5.697306 |  |  |
|  |  |  |  |  |  |  |  | WNT16 | 5.698915 |  |  |
|  |  |  |  |  |  |  |  | DUOX1 | 5.704927 |  |  |
|  |  |  |  |  |  |  |  | PLLP | 5.707952 |  |  |
|  |  |  |  |  |  |  |  | ESRP2 | 5.725895 |  |  |
|  |  |  |  |  |  |  |  | IDE | 5.740143 |  |  |
|  |  |  |  |  |  |  |  | GRHL3 | 5.740893 |  |  |
|  |  |  |  |  |  |  |  | KRT16 | 5.747316 |  |  |
|  |  |  |  |  |  |  |  | DHCR7 | 5.754665 |  |  |
|  |  |  |  |  |  |  |  | KRT71 | 5.759212 |  |  |
|  |  |  |  |  |  |  |  | LGALSL | 5.76079 |  |  |
|  |  |  |  |  |  |  |  | KRT79 | 5.770893 |  |  |
|  |  |  |  |  |  |  |  | NIPAL1 | 5.771694 |  |  |
|  |  |  |  |  |  |  |  | LYPD6B | 5.775212 |  |  |
|  |  |  |  |  |  |  |  | WNT4 | 5.776378 |  |  |
|  |  |  |  |  |  |  |  | GALNT3 | 5.795184 |  |  |
|  |  |  |  |  |  |  |  | AQP3 | 5.805554 |  |  |
|  |  |  |  |  |  |  |  | PSAPL1 | 5.855469 |  |  |
|  |  |  |  |  |  |  |  | VSNL1 | 5.861633 |  |  |
|  |  |  |  |  |  |  |  | GRTP1 | 5.873485 |  |  |
|  |  |  |  |  |  |  |  | PDZK1IP1 | 5.880075 |  |  |
|  |  |  |  |  |  |  |  | ARHGEF37 | 5.894677 |  |  |
|  |  |  |  |  |  |  |  | FGFBP1 | 5.897505 |  |  |
|  |  |  |  |  |  |  |  | SULT2B1 | 5.93132 |  |  |
|  |  |  |  |  |  |  |  | RAPGEFL1 | 5.949971 |  |  |
|  |  |  |  |  |  |  |  | CDS1 | 5.98972 |  |  |
|  |  |  |  |  |  |  |  | ALOX12B | 6.004873 |  |  |
|  |  |  |  |  |  |  |  | KCNK7 | 6.013497 |  |  |
|  |  |  |  |  |  |  |  | KRT25 | 6.021401 |  |  |
|  |  |  |  |  |  |  |  | CORIN | 6.070705 |  |  |
|  |  |  |  |  |  |  |  | HOOK1 | 6.082304 |  |  |
|  |  |  |  |  |  |  |  | LAMB4 | 6.087256 |  |  |
|  |  |  |  |  |  |  |  | RPS4Y1 | 6.097804 |  |  |
|  |  |  |  |  |  |  |  | LAD1 | 6.098184 |  |  |
|  |  |  |  |  |  |  |  | IL20RA | 6.100266 |  |  |
|  |  |  |  |  |  |  |  | SGPP2 | 6.149084 |  |  |
|  |  |  |  |  |  |  |  | FAM46B | 6.157356 |  |  |
|  |  |  |  |  |  |  |  | PPP1R14C | 6.161331 |  |  |
|  |  |  |  |  |  |  |  | MLANA | 6.16346 |  |  |
|  |  |  |  |  |  |  |  | WIF1 | 6.171771 |  |  |
|  |  |  |  |  |  |  |  | MIR4720 | 6.1816 |  |  |
|  |  |  |  |  |  |  |  | FAT2 | 6.19138 |  |  |
|  |  |  |  |  |  |  |  | PPP1R1B | 6.198731 |  |  |
|  |  |  |  |  |  |  |  | CLCA4 | 6.205419 |  |  |
|  |  |  |  |  |  |  |  | RAB38 | 6.224439 |  |  |
|  |  |  |  |  |  |  |  | PKP3 | 6.234307 |  |  |
|  |  |  |  |  |  |  |  | NIPAL4 | 6.240822 |  |  |
|  |  |  |  |  |  |  |  | EPHX3 | 6.247405 |  |  |
|  |  |  |  |  |  |  |  | CELSR2 | 6.250427 |  |  |
|  |  |  |  |  |  |  |  | MPZL3 | 6.279309 |  |  |
|  |  |  |  |  |  |  |  | TMEM40 | 6.27955 |  |  |
|  |  |  |  |  |  |  |  | CLDN8 | 6.322483 |  |  |
|  |  |  |  |  |  |  |  | TFAP2B | 6.348335 |  |  |
|  |  |  |  |  |  |  |  | GJB3 | 6.349376 |  |  |
|  |  |  |  |  |  |  |  | RNASE7 | 6.350791 |  |  |
|  |  |  |  |  |  |  |  | KRT7 | 6.363214 |  |  |
|  |  |  |  |  |  |  |  | MSMB | 6.369759 |  |  |
|  |  |  |  |  |  |  |  | KRT31 | 6.390556 |  |  |
|  |  |  |  |  |  |  |  | TMEM97 | 6.392611 |  |  |
|  |  |  |  |  |  |  |  | ABCA12 | 6.417625 |  |  |
|  |  |  |  |  |  |  |  | APCDD1 | 6.425564 |  |  |
|  |  |  |  |  |  |  |  | DNER | 6.441115 |  |  |
|  |  |  |  |  |  |  |  | HS3ST6 | 6.453188 |  |  |
|  |  |  |  |  |  |  |  | ALOX15B | 6.454137 |  |  |
|  |  |  |  |  |  |  |  | PCP4 | 6.471056 |  |  |
|  |  |  |  |  |  |  |  | CLIC3 | 6.478304 |  |  |
|  |  |  |  |  |  |  |  | CERS3 | 6.489796 |  |  |
|  |  |  |  |  |  |  |  | CD1A | 6.521185 |  |  |
|  |  |  |  |  |  |  |  | DHCR24 | 6.525562 |  |  |
|  |  |  |  |  |  |  |  | EPS8L1 | 6.539854 |  |  |
|  |  |  |  |  |  |  |  | BCL11B | 6.5415 |  |  |
|  |  |  |  |  |  |  |  | TTC22 | 6.543174 |  |  |
|  |  |  |  |  |  |  |  | RNF128 | 6.565674 |  |  |
|  |  |  |  |  |  |  |  | CHP2 | 6.57418 |  |  |
|  |  |  |  |  |  |  |  | ACSBG1 | 6.618401 |  |  |
|  |  |  |  |  |  |  |  | FERMT1 | 6.630563 |  |  |
|  |  |  |  |  |  |  |  | GJB5 | 6.698514 |  |  |
|  |  |  |  |  |  |  |  | WNT3 | 6.700826 |  |  |
|  |  |  |  |  |  |  |  | PSORS1C2 | 6.71853 |  |  |
|  |  |  |  |  |  |  |  | PNLIPRP3 | 6.718888 |  |  |
|  |  |  |  |  |  |  |  | CEACAM6 | 6.726154 |  |  |
|  |  |  |  |  |  |  |  | SPINT1 | 6.734331 |  |  |
|  |  |  |  |  |  |  |  | PAK6 | 6.742159 |  |  |
|  |  |  |  |  |  |  |  | PTK6 | 6.748156 |  |  |
|  |  |  |  |  |  |  |  | IRX2 | 6.751991 |  |  |
|  |  |  |  |  |  |  |  | GAL | 6.75249 |  |  |
|  |  |  |  |  |  |  |  | MFAP3L | 6.772104 |  |  |
|  |  |  |  |  |  |  |  | TINCR | 6.790576 |  |  |
|  |  |  |  |  |  |  |  | TCHH | 6.797746 |  |  |
|  |  |  |  |  |  |  |  | TFAP2C | 6.801791 |  |  |
|  |  |  |  |  |  |  |  | SPRR2G | 6.820094 |  |  |
|  |  |  |  |  |  |  |  | CLDN1 | 6.881038 |  |  |
|  |  |  |  |  |  |  |  | CARD18 | 6.892551 |  |  |
|  |  |  |  |  |  |  |  | CD207 | 6.944657 |  |  |
|  |  |  |  |  |  |  |  | ASPRV1 | 6.948766 |  |  |
|  |  |  |  |  |  |  |  | KLK10 | 6.969392 |  |  |
|  |  |  |  |  |  |  |  | MPP7 | 6.989282 |  |  |
|  |  |  |  |  |  |  |  | CASZ1 | 7.000178 |  |  |
|  |  |  |  |  |  |  |  | RDH12 | 7.044394 |  |  |
|  |  |  |  |  |  |  |  | HAL | 7.046316 |  |  |
|  |  |  |  |  |  |  |  | FGFR2 | 7.062792 |  |  |
|  |  |  |  |  |  |  |  | PPP2R2C | 7.063688 |  |  |
|  |  |  |  |  |  |  |  | BPIFC | 7.069824 |  |  |
|  |  |  |  |  |  |  |  | CTSV | 7.07595 |  |  |
|  |  |  |  |  |  |  |  | C1orf106 | 7.085802 |  |  |
|  |  |  |  |  |  |  |  | CAPNS2 | 7.117831 |  |  |
|  |  |  |  |  |  |  |  | NEBL | 7.12593 |  |  |
|  |  |  |  |  |  |  |  | C1orf68 | 7.12813 |  |  |
|  |  |  |  |  |  |  |  | BCL11A | 7.155437 |  |  |
|  |  |  |  |  |  |  |  | SERPINB13 | 7.172537 |  |  |
|  |  |  |  |  |  |  |  | CWH43 | 7.192431 |  |  |
|  |  |  |  |  |  |  |  | PTPRZ1 | 7.223162 |  |  |
|  |  |  |  |  |  |  |  | GGT6 | 7.23433 |  |  |
|  |  |  |  |  |  |  |  | SH3RF2 | 7.290161 |  |  |
|  |  |  |  |  |  |  |  | CRCT1 | 7.301331 |  |  |
|  |  |  |  |  |  |  |  | KRT19 | 7.306382 |  |  |
|  |  |  |  |  |  |  |  | NMU | 7.316725 |  |  |
|  |  |  |  |  |  |  |  | LYNX1 | 7.325595 |  |  |
|  |  |  |  |  |  |  |  | SERPINA12 | 7.36314 |  |  |
|  |  |  |  |  |  |  |  | C19orf33 | 7.383661 |  |  |
|  |  |  |  |  |  |  |  | DGAT2 | 7.390115 |  |  |
|  |  |  |  |  |  |  |  | KLF5 | 7.390125 |  |  |
|  |  |  |  |  |  |  |  | POU2F3 | 7.391597 |  |  |
|  |  |  |  |  |  |  |  | CDHR1 | 7.416829 |  |  |
|  |  |  |  |  |  |  |  | TSPAN8 | 7.419307 |  |  |
|  |  |  |  |  |  |  |  | KLC3 | 7.428656 |  |  |
|  |  |  |  |  |  |  |  | FOXN1 | 7.430686 |  |  |
|  |  |  |  |  |  |  |  | KLK8 | 7.44746 |  |  |
|  |  |  |  |  |  |  |  | NSG1 | 7.493236 |  |  |
|  |  |  |  |  |  |  |  | SOSTDC1 | 7.514056 |  |  |
|  |  |  |  |  |  |  |  | SCNN1A | 7.526065 |  |  |
|  |  |  |  |  |  |  |  | GPR87 | 7.543604 |  |  |
|  |  |  |  |  |  |  |  | CNFN | 7.553355 |  |  |
|  |  |  |  |  |  |  |  | HOPX | 7.569376 |  |  |
|  |  |  |  |  |  |  |  | TP63 | 7.582611 |  |  |
|  |  |  |  |  |  |  |  | FAM84A | 7.609298 |  |  |
|  |  |  |  |  |  |  |  | KLK7 | 7.618761 |  |  |
|  |  |  |  |  |  |  |  | IRF6 | 7.637007 |  |  |
|  |  |  |  |  |  |  |  | CHMP4C | 7.641055 |  |  |
|  |  |  |  |  |  |  |  | GATA3 | 7.643234 |  |  |
|  |  |  |  |  |  |  |  | C1orf116 | 7.656828 |  |  |
|  |  |  |  |  |  |  |  | COL17A1 | 7.658445 |  |  |
|  |  |  |  |  |  |  |  | BNIPL | 7.660737 |  |  |
|  |  |  |  |  |  |  |  | LINC00302 | 7.664208 |  |  |
|  |  |  |  |  |  |  |  | EXPH5 | 7.756578 |  |  |
|  |  |  |  |  |  |  |  | CCL27 | 7.817083 |  |  |
|  |  |  |  |  |  |  |  | PROM2 | 7.831421 |  |  |
|  |  |  |  |  |  |  |  | S100A7 | 7.876025 |  |  |
|  |  |  |  |  |  |  |  | LY6G6C | 7.884491 |  |  |
|  |  |  |  |  |  |  |  | POF1B | 7.932608 |  |  |
|  |  |  |  |  |  |  |  | THRSP | 7.948632 |  |  |
|  |  |  |  |  |  |  |  | FAM83C | 7.968782 |  |  |
|  |  |  |  |  |  |  |  | KRT80 | 7.977691 |  |  |
|  |  |  |  |  |  |  |  | BBOX1 | 7.981566 |  |  |
|  |  |  |  |  |  |  |  | SFN | 8.000928 |  |  |
|  |  |  |  |  |  |  |  | GRHL1 | 8.004028 |  |  |
|  |  |  |  |  |  |  |  | F2RL1 | 8.058651 |  |  |
|  |  |  |  |  |  |  |  | SLURP1 | 8.106158 |  |  |
|  |  |  |  |  |  |  |  | ERBB3 | 8.115605 |  |  |
|  |  |  |  |  |  |  |  | PERP | 8.158846 |  |  |
|  |  |  |  |  |  |  |  | FAM83B | 8.169686 |  |  |
|  |  |  |  |  |  |  |  | KRT23 | 8.215126 |  |  |
|  |  |  |  |  |  |  |  | CA6 | 8.228676 |  |  |
|  |  |  |  |  |  |  |  | MUC15 | 8.243413 |  |  |
|  |  |  |  |  |  |  |  | DSG3 | 8.253097 |  |  |
|  |  |  |  |  |  |  |  | SERPINB2 | 8.322978 |  |  |
|  |  |  |  |  |  |  |  | IL20RB | 8.352434 |  |  |
|  |  |  |  |  |  |  |  | AADACL2 | 8.366701 |  |  |
|  |  |  |  |  |  |  |  | C5orf46 | 8.370348 |  |  |
|  |  |  |  |  |  |  |  | IL37 | 8.390953 |  |  |
|  |  |  |  |  |  |  |  | SERPINB7 | 8.403275 |  |  |
|  |  |  |  |  |  |  |  | ARG1 | 8.405536 |  |  |
|  |  |  |  |  |  |  |  | CALML3 | 8.419063 |  |  |
|  |  |  |  |  |  |  |  | KLK11 | 8.456982 |  |  |
|  |  |  |  |  |  |  |  | SDR16C5 | 8.533585 |  |  |
|  |  |  |  |  |  |  |  | FOXQ1 | 8.538418 |  |  |
|  |  |  |  |  |  |  |  | ALDH3B2 | 8.544974 |  |  |
|  |  |  |  |  |  |  |  | KLK5 | 8.636782 |  |  |
|  |  |  |  |  |  |  |  | FABP7 | 8.691696 |  |  |
|  |  |  |  |  |  |  |  | RAB25 | 8.729076 |  |  |
|  |  |  |  |  |  |  |  | FXYD3 | 8.749561 |  |  |
|  |  |  |  |  |  |  |  | TRIM29 | 8.794867 |  |  |
|  |  |  |  |  |  |  |  | DCT | 8.866043 |  |  |
|  |  |  |  |  |  |  |  | S100A14 | 8.881202 |  |  |
|  |  |  |  |  |  |  |  | CDSN | 8.905182 |  |  |
|  |  |  |  |  |  |  |  | TFAP2A | 8.918655 |  |  |
|  |  |  |  |  |  |  |  | WFDC5 | 8.922845 |  |  |
|  |  |  |  |  |  |  |  | S100A2 | 8.924232 |  |  |
|  |  |  |  |  |  |  |  | CLCA2 | 8.95478 |  |  |
|  |  |  |  |  |  |  |  | DAPL1 | 8.96235 |  |  |
|  |  |  |  |  |  |  |  | LY6D | 8.991377 |  |  |
|  |  |  |  |  |  |  |  | EPPK1 | 9.042592 |  |  |
|  |  |  |  |  |  |  |  | ESRP1 | 9.102663 |  |  |
|  |  |  |  |  |  |  |  | SPRR1A | 9.114648 |  |  |
|  |  |  |  |  |  |  |  | KRT6A | 9.226653 |  |  |
|  |  |  |  |  |  |  |  | SCEL | 9.22904 |  |  |
|  |  |  |  |  |  |  |  | KRT77 | 9.23801 |  |  |
|  |  |  |  |  |  |  |  | PIP | 9.238231 |  |  |
|  |  |  |  |  |  |  |  | SCGB1D2 | 9.270478 |  |  |
|  |  |  |  |  |  |  |  | EHF | 9.303703 |  |  |
|  |  |  |  |  |  |  |  | SPRR1B | 9.308266 |  |  |
|  |  |  |  |  |  |  |  | LYPD3 | 9.563039 |  |  |
|  |  |  |  |  |  |  |  | SPINK5 | 9.603987 |  |  |
|  |  |  |  |  |  |  |  | MAL2 | 9.680962 |  |  |
|  |  |  |  |  |  |  |  | LCE2B | 9.791383 |  |  |
|  |  |  |  |  |  |  |  | ZNF750 | 9.870122 |  |  |
|  |  |  |  |  |  |  |  | DST | 10.01274 |  |  |
|  |  |  |  |  |  |  |  | PKP1 | 10.03168 |  |  |
|  |  |  |  |  |  |  |  | CALML5 | 10.04204 |  |  |
|  |  |  |  |  |  |  |  | TACSTD2 | 10.09982 |  |  |
|  |  |  |  |  |  |  |  | TYRP1 | 10.21171 |  |  |
|  |  |  |  |  |  |  |  | CDH1 | 10.24253 |  |  |
|  |  |  |  |  |  |  |  | AZGP1 | 10.24609 |  |  |
|  |  |  |  |  |  |  |  | KRT15 | 10.25674 |  |  |
|  |  |  |  |  |  |  |  | KRT6B | 10.38047 |  |  |
|  |  |  |  |  |  |  |  | CST6 | 10.47271 |  |  |
|  |  |  |  |  |  |  |  | SERPINB5 | 10.50302 |  |  |
|  |  |  |  |  |  |  |  | SBSN | 10.54567 |  |  |
|  |  |  |  |  |  |  |  | LCE1B | 10.56922 |  |  |
|  |  |  |  |  |  |  |  | DSC3 | 10.84372 |  |  |
|  |  |  |  |  |  |  |  | DSP | 10.95681 |  |  |
|  |  |  |  |  |  |  |  | MUCL1 | 11.03477 |  |  |
|  |  |  |  |  |  |  |  | FLG | 11.16012 |  |  |
|  |  |  |  |  |  |  |  | DMKN | 11.31906 |  |  |
|  |  |  |  |  |  |  |  | LOR | 11.49691 |  |  |
|  |  |  |  |  |  |  |  | SCGB2A2 | 11.5049 |  |  |
|  |  |  |  |  |  |  |  | DSG1 | 11.56079 |  |  |
|  |  |  |  |  |  |  |  | KRT2 | 11.77029 |  |  |
|  |  |  |  |  |  |  |  | DSC1 | 11.84084 |  |  |
|  |  |  |  |  |  |  |  | KRT5 | 11.90682 |  |  |
|  |  |  |  |  |  |  |  | FLG2 | 12.02491 |  |  |
|  |  |  |  |  |  |  |  | KRTDAP | 12.07071 |  |  |
|  |  |  |  |  |  |  |  | KRT1 | 12.27193 |  |  |
|  |  |  |  |  |  |  |  | DCD | 12.65814 |  |  |
|  |  |  |  |  |  |  |  | KRT14 | 13.28593 |  |  |
