# Supplementary material for: Comorbidities of Psoriasis - Exploring the Links by Network Approach
Source: PLoS One. 2016 Mar 11;11(3):e0149175. doi: 10.1371/journal.pone.0149175 (PMC4788348; doi:10.1371/journal.pone.0149175)
Supplement: S2 Table — The common biological processes between psoriasis and its comorbidities were highlighted. (DOCX) [file pone.0149175.s002.docx]

**S2 Table** : Biological process involved in each disease category. The common biological processes between psoriasis and its comorbidities are highlighted.

| **Alzheimer’s disease** | **Myocardial infraction** | **Type 2 diabetes** | **Obesity** | **Rheumatoid arthritis** | | |
| --- | --- | --- | --- | --- | --- | --- |
| **GO:0006952~**  **Defence response** | **GO:0006952~**  **Defence response** | **GO:0006952~**  **Defence response** | GO:0002252~  Immune effector process | GO:0001775~  Cell activation | **GO:0006954~**  **Inflammatory response** | GO:0019363~  Pyridine nucleotide biosynthetic process |
| **GO:0006954~**  **Inflammatory response** | **GO:0006954~**  **Inflammatory response** | **GO:0006954~**  **Inflammatory response** | GO:0002526~  Acute inflammatory response | GO:0001906~  Cell killing | **GO:0006955~**  **Immune response** | GO:0019674~  NAD metabolic process |
| **GO:0006955~**  **Immune response** | **GO:0006955~**  **Immune response** | **GO:0006955~**  **Immune response** | GO:0006935~  Chemotaxis | GO:0002237~  Response to molecule of bacterial origin | GO:0006968~  Cellular defence response | GO:0019748~  Secondary metabolic process |
| GO:0007267~  Cell-cell signaling | GO:0009611~  Response to wounding | GO:0007267~  Cell-cell signaling | **GO:0006952~**  **Defence response** | GO:0002238~  Response to molecule of fungal origin | GO:0007155~  Cell adhesion | GO:0019835~  Cytolysis |
| GO:0009611~  Response to wounding | GO:0016485~  Protein processing | GO:0009615~  Response to virus | **GO:0006954~**  **Inflammatory response** | GO:0002366~  Leukocyte activation during immune response | GO:0007204~  Elevation of cytosolic calcium ion concentration | GO:0022415~  Viral reproductive process |
|  |  | GO:0010639~  Negative regulation of organelle organization | **GO:0006955~**  **Immune response** | GO:0002429~  Immune response-activating cell surface receptor signaling pathway | GO:0007267~  Cell-cell signaling | GO:0022610~  Biological adhesion |
|  |  | GO:0031349~  Positive regulation of defence response | GO:0007610~  Behaviour | GO:0002684~  Positive regulation of immune system process | GO:0007610~  Behaviour | GO:0032496~  Response to lipopolysaccharide |
|  |  | GO:0033043~  Regulation of organelle organization | GO:0007626~  Locomotory behaviour | GO:0002752~  Cell surface pattern recognition receptor signaling pathway | GO:0007626~  Locomotory behaviour | GO:0034097~  Response to cytokine stimulus |
|  |  | GO:0042445~  Hormone metabolic process | GO:0009611~  Response to wounding | GO:0002764~  Immune response-regulating signal transduction | GO:0008219~  Cell death | GO:0042110~  T cell activation |
|  |  | GO:0042493~  Response to drug | GO:0016485~  Protein processing | GO:0002768~  Immune response-regulating cell surface receptor signaling pathway | GO:0009435~  NAD biosynthetic process | GO:0042981~  Regulation of apoptosis |
|  |  | GO:0051094~  Positive regulation of developmental process | GO:0019674~  NAD metabolic process | GO:0006766~  Vitamin metabolic process | GO:0009611~  Response to wounding | GO:0045087~  Innate immune response |
|  |  |  | GO:0042742~  Defence response to bacterium | GO:0006873~  Cellular ion homeostasis | GO:0009615~  Response to virus | GO:0046649~  Lymphocyte activation |
|  |  |  |  | GO:0006874~  Cellular calcium ion homeostasis | GO:0009617~  Response to bacterium | GO:0048584~  Positive regulation of response to stimulus |
|  |  |  |  | GO:0006875~  Cellular metal ion homeostasis | GO:0009620~  Response to fungus | GO:0050778~  Positive regulation of immune response |
|  |  |  |  | GO:0006915~  Apoptosis | GO:0010941~  Regulation of cell death | GO:0050801~  Ion homeostasis |
|  |  |  |  | GO:0006922~  Cleavage of lamin | GO:0012501~  Programmed cell death | GO:0051480~  Cytosolic calcium ion homeostasis |
|  |  |  |  | GO:0006923~  Cleavage of cytoskeletal proteins during apoptosis | GO:0016032~  Viral reproduction | GO:0055065~  Metal ion homeostasis |
|  |  |  |  | GO:0006928~  Cell motion | GO:0016265~  Death | GO:0055074~  Calcium ion homeostasis |
|  |  |  |  | GO:0006935~  Chemotaxis | GO:0019058~  Viral infectious cycle | GO:0055080~  Cation homeostasis |
|  |  |  |  | **GO:0006952~**  **Defence response** | GO:0019359~  Nicotinamide nucleotide biosynthetic process | GO:0055082~  Cellular chemical homeostasis |
