# Supplementary material for: Comorbidities of Psoriasis - Exploring the Links by Network Approach
Source: PLoS One. 2016 Mar 11;11(3):e0149175. doi: 10.1371/journal.pone.0149175 (PMC4788348; doi:10.1371/journal.pone.0149175)
Supplement: S3 Table — The common biological pathways between psoriasis and its comorbidities are highlighted. (DOCX) [file pone.0149175.s003.docx]

**S3 Table**: Biological pathways involved in each disease category. The common biological pathways between psoriasis and its comorbidities are highlighted.

| **Alzheimer’s disease** | **Myocardial infraction** | **Type 2 diabetes** | | **Obesity** | **Rheumatoid arthritis** | |
| --- | --- | --- | --- | --- | --- | --- |
| P00004~  Alzheimer disease-presenilin pathway | **P00005~**  **Angiogenesis** | P00003~  Alzheimer disease-amyloid secretase pathway | P00049~  Parkinson disease | P00003~  Alzheimer disease-amyloid secretase pathway | P00003~  Alzheimer disease-amyloid secretase pathway | P00044~  Nicotinic acetylcholine receptor signaling pathway |
| **P00005~**  **Angiogenesis** | P00006~  Apoptosis signaling pathway | P00004~  Alzheimer disease-presenilin pathway | P00052~  TGF-beta signaling pathway | P00004~  Alzheimer disease-presenilin pathway | P00004~  Alzheimer disease-presenilin pathway | P00046~  Oxidative stress response |
| P00008~  Axon guidance mediated by Slit/Robo | **P00026~**  **Heterotrimeric G-protein signaling pathway-Gi alpha and Gs alpha mediated pathway** | **P00005~**  **Angiogenesis** | P00053~  T cell activation | **P00005~ Angiogenesis** | **P00005~**  **Angiogenesis** | P00047~  PDGF signaling pathway |
| P00009~  Axon guidance mediated by netrin | **P00027~**  **Heterotrimeric G-protein signaling pathway-Gq alpha and Go alpha mediated pathway** | P00006~  Apoptosis signaling pathway | P00054~  Toll receptor signaling pathway | P00011~  Blood coagulation | P00006~  Apoptosis signaling pathway | P00049~  Parkinson disease |
| P00011~  Blood coagulation | **P00031~**  **Inflammation mediated by chemokine and cytokine signaling pathway** | P00008~  Axon guidance mediated by Slit/Robo | P00056~  VEGF signaling pathway | P00012~  Cadherin signaling pathway | P00008~  Axon guidance mediated by Slit/Robo | P00050~  Plasminogen activating cascade |
| P00012~  Cadherin signaling pathway | **P00034~**  **Integrin signalling pathway** | P00011~  Blood coagulation | **P00057~**  **Wnt signaling pathway** | P00018~  EGF receptor signaling pathway | P00009~  Axon guidance mediated by netrin | P00052~  TGF-beta signaling pathway |
| P00016~  Cytoskeletal regulation by Rho GTPase | P00035~  Interferon-gamma signaling pathway | P00012~  Cadherin signaling pathway | P00059~  p53 pathway | P00020~  FAS signaling pathway | P00011~  Blood coagulation | P00053~  T cell activation |
| P00021~  FGF signaling pathway | P00038~  JAK/STAT signaling pathway | P00016~  Cytoskeletal regulation by Rho GTPase | P00060~  Ubiquitin proteasome pathway | P00021~  FGF signaling pathway | P00012~  Cadherin signaling pathway | P00054~  Toll receptor signaling pathway |
| **P00026~**  **Heterotrimeric G-protein signaling pathway-Gi alpha and Gs alpha mediated pathway** | P00047~  PDGF signaling pathway | P00018~  EGF receptor signaling pathway | P02723~  Adenine and hypoxanthine salvage pathway | P00024~  Glycolysis | P00013~  Cell cycle | P00056~  VEGF signaling pathway |
| **P00027~**  **Heterotrimeric G-protein signaling pathway-Gq alpha and Go alpha mediated pathway** | P00054~  Toll receptor signaling pathway | P00019~  Endothelin signaling pathway | P02727~  Androgen/estrogene/progesterone biosynthesis | **P00026~ Heterotrimeric G-protein signaling pathway-Gi alpha and Gs alpha mediated pathway** | P00016~  Cytoskeletal regulation by Rho GTPase | **P00057~**  **Wnt signaling pathway** |
| **P00031~**  **Inflammation mediated by chemokine and cytokine signaling pathway** | **P00057~**  **Wnt signaling pathway** | P00021~  FGF signaling pathway | P02738~  De novo purine biosynthesis | **P00027~ Heterotrimeric G-protein signaling pathway-Gq alpha and Go alpha mediated pathway** | P00017~  DNA replication | P00059~ p53 pathway |
| **P00034~**  **Integrin signalling pathway** | **P06664~**  **Gonadotropin-releasing hormone receptor pathway** | P00024~  Glycolysis | P02739~  De novo pyrimidine deoxyribonucleotide biosynthesis | **P00031~ Inflammation mediated by chemokine and cytokine signaling pathway** | P00018~  EGF receptor signaling pathway | P00060~  Ubiquitin proteasome pathway |
| P00037~  Ionotropic glutamate receptor pathway | **P06959~**  **CCKR signaling map** | P00025~  Hedgehog signaling pathway | P02740~  De novo pyrimidine ribonucleotides biosythesis | **P00034~**  **Integrin signalling pathway** | P00019~  Endothelin signaling pathway | P02738~  De novo purine biosynthesis |
| P00039~  Metabotropic glutamate receptor group III pathway |  | **P00026~**  **Heterotrimeric G-protein signaling pathway-Gi alpha and Gs alpha mediated pathway** | P02762~  Pentose phosphate pathway | P00036~  Interleukin signaling pathway | P00020~  FAS signaling pathway | P02739~  De novo pyrimidine deoxyribonucleotide biosynthesis |
| P00042~  Muscarinic acetylcholine receptor 1 and 3 signaling pathway |  | **P00027~**  **Heterotrimeric G-protein signaling pathway-Gq alpha and Go alpha mediated pathway** | P02788~  Xanthine and guanine salvage pathway | P00037~  Ionotropic glutamate receptor pathway | P00021~  FGF signaling pathway | P02740~  De novo pyrimidine ribonucleotides biosythesis |
| P00044~  Nicotinic acetylcholine receptor signaling pathway |  | P00031~  Inflammation mediated by chemokine and cytokine signaling pathway | P04374~  5HT2 type receptor mediated signaling pathway | P00039~ Metabotropic glutamate receptor group III pathway | P00024~  Glycolysis | P02743~ Formyltetrahydroformate biosynthesis |
| P00053~  T cell activation |  | P00033~  Insulin/IGF pathway-protein kinase B signaling cascade | P04377~  Beta1 adrenergic receptor signaling pathway | P00042~  Muscarinic acetylcholine receptor 1 and 3 signaling pathway | P00025~  Hedgehog signaling pathway | P02744~  Fructose galactose metabolism |
| P00054~  Toll receptor signaling pathway |  | **P00034~**  **Integrin signalling pathway** | P04378~  Beta2 adrenergic receptor signaling pathway | P00047~  PDGF signaling pathway | **P00026~ Heterotrimeric G-protein signaling pathway-Gi alpha and Gs alpha mediated pathway** | P02769~  Purine metabolism |
| **P00057~**  **Wnt signaling pathway** |  | P00035~  Interferon-gamma signaling pathway | P04385~  Histamine H1 receptor mediated signaling pathway | P00050~ Plasminogen activating cascade | **P00027~ Heterotrimeric G-protein signaling pathway-Gq alpha and Go alpha mediated pathway** | P02774~  Salvage pyrimidine deoxyribonucleotides |
| **P06664~**  **Gonadotropin-releasing hormone receptor pathway** |  | P00036~  Interleukin signaling pathway | P04391~  Oxytocin receptor mediated signaling pathway | **P00057~**  **Wnt signaling pathway** | P00030~  Hypoxia response via HIF activation | P02775~  Salvage pyrimidine ribonucleotides |
| **P06959~**  **CCKR signaling map** |  | P00037~  Ionotropic glutamate receptor pathway | P04394~  Thyrotropin-releasing hormone receptor signaling pathway | P00059~  p53 pathway | **P00031~**  **Inflammation mediated by chemokine and cytokine signaling pathway** | P04374~  5HT2 type receptor mediated signaling pathway |
|  |  | P00039~  Metabotropic glutamate receptor group III pathway | P04395~  Vasopressin synthesis | P00060~  Ubiquitin proteasome pathway | P00033~  Insulin/IGF pathway-protein kinase B signaling cascade | P04377~  Beta1 adrenergic receptor signaling pathway |
|  |  | P00042~  Muscarinic acetylcholine receptor 1 and 3 signaling pathway | P04398~  p53 pathway feedback loops 2 | P02738~  De novo purine biosynthesis | **P00034~**  **Integrin signalling pathway** | P04378~  Beta2 adrenergic receptor signaling pathway |
|  |  | P00044~  Nicotinic acetylcholine receptor signaling pathway | **P06664~**  **Gonadotropin-releasing hormone receptor pathway** | P02739~  De novo pyrimidine deoxyribonucleotide biosynthesis | P00035~  Interferon-gamma signaling pathway | P04385~  Histamine H1 receptor mediated signaling pathway |
|  |  | P00047~  PDGF signaling pathway | **P06959~**  **CCKR signaling map** | P04374~  5HT2 type receptor mediated signaling pathway | P00036~  Interleukin signaling pathway | P04391~  Oxytocin receptor mediated signaling pathway |
|  |  |  |  | P04377~  Beta1 adrenergic receptor signaling pathway | P00037~  Ionotropic glutamate receptor pathway | P04393~  Ras Pathway |
|  |  |  |  | P04378~  Beta2 adrenergic receptor signaling pathway | P00038~  JAK/STAT signaling pathway | P04398~  p53 pathway feedback loops 2 |
|  |  |  |  | P04391~  Oxytocin receptor mediated signaling pathway | P00039~  Metabotropic glutamate receptor group III pathway | **P06664~ Gonadotropin-releasing hormone receptor pathway** |
|  |  |  |  | P04393~  Ras Pathway | P00042~  Muscarinic acetylcholine receptor 1 and 3 signaling pathway | **P06959~**  **CCKR signaling map** |
|  |  |  |  | P04394~ Thyrotropin-releasing hormone receptor signaling pathway |  |  |
|  |  |  |  | **P06664~ Gonadotropin-releasing hormone receptor pathway** |  |  |
|  |  |  |  | **P06959~ CCKR signaling map** |  |  |
